# The individual and global impact of copy-number variants on complex human traits

**DOI:** 10.1016/j.ajhg.2022.02.010

**Published:** 2022-03-02

**Authors:** Chiara Auwerx, Maarja Lepamets, Marie C. Sadler, Marion Patxot, Miloš Stojanov, David Baud, Reedik Mägi, Estonian Biobank Research Team, Tõnu Esko, Andres Metspalu, Lili Milani, Reedik Mägi, Mari Nelis, Eleonora Porcu, Alexandre Reymond, Zoltán Kutalik

**Affiliations:** 1 Center for Integrative Genomics, University of Lausanne, Lausanne 1015, Switzerland; 2 Department of Computational Biology, University of Lausanne, Lausanne 1015, Switzerland; 3 Swiss Institute of Bioinformatics, Lausanne 1015, Switzerland; 4 University Center for Primary Care and Public Health, Lausanne 1010, Switzerland; 5 Institute of Molecular and Cell Biology, University of Tartu, Tartu 51010, Estonia; 6 Estonian Genome Centre, Institute of Genomics, University of Tartu, Tartu 51010, Estonia; 7 Materno-fetal and Obstetrics Research Unit, Department Woman-Mother-Child, CHUV, Lausanne 1011, Switzerland

**Keywords:** CNV, GWAS, lifespan, mutational burden, pleiotropy, polygenicity, penetrance, structural variants, UK Biobank, variable expressivity

## Abstract

The impact of copy-number variations (CNVs) on complex human traits remains understudied. We called CNVs in 331,522 UK Biobank participants and performed genome-wide association studies (GWASs) between the copy number of CNV-proxy probes and 57 continuous traits, revealing 131 signals spanning 47 phenotypes. Our analysis recapitulated well-known associations (e.g., 1q21 and height), revealed the pleiotropy of recurrent CNVs (e.g., 26 and 16 traits for 16p11.2-BP4-BP5 and 22q11.21, respectively), and suggested gene functionalities (e.g., *MARF1* in female reproduction). Forty-eight CNV signals (38%) overlapped with single-nucleotide polymorphism (SNP)-GWASs signals for the same trait. For instance, deletion of *PDZK1*, which encodes a urate transporter scaffold protein, decreased serum urate levels, while deletion of *RHD*, which encodes the Rhesus blood group D antigen, associated with hematological traits. Other signals overlapped Mendelian disorder regions, suggesting variable expressivity and broad impact of these loci, as illustrated by signals mapping to Rotor syndrome (*SLCO1B1/3*), renal cysts and diabetes syndrome (*HNF1B*), or Charcot-Marie-Tooth (*PMP22*) loci. Total CNV burden negatively impacted 35 traits, leading to increased adiposity, liver/kidney damage, and decreased intelligence and physical capacity. Thirty traits remained burden associated after correcting for CNV-GWAS signals, pointing to a polygenic CNV architecture. The burden negatively correlated with socio-economic indicators, parental lifespan, and age (survivorship proxy), suggesting a contribution to decreased longevity. Together, our results showcase how studying CNVs can expand biological insights, emphasizing the critical role of this mutational class in shaping human traits and arguing in favor of a continuum between Mendelian and complex diseases.

## Graphical abstract

**Figure:**
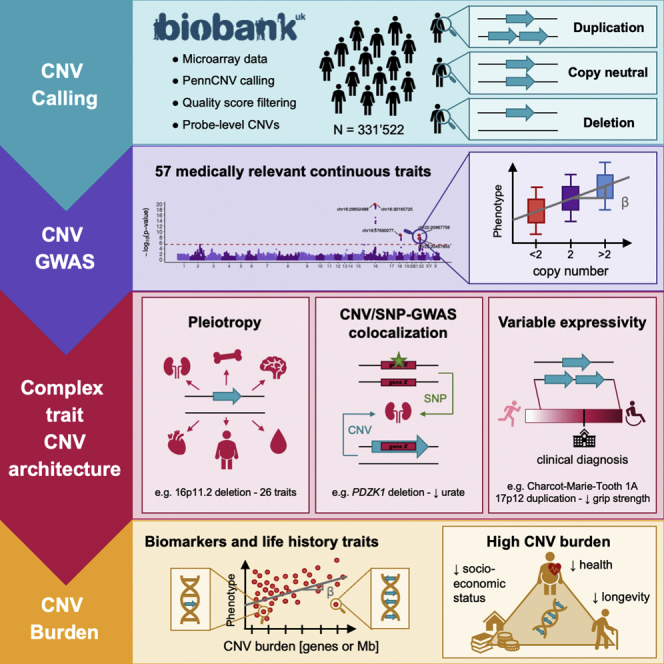

## Introduction

With the advent of genome-wide associations studies (GWASs), the polygenic architecture of complex human traits has become apparent.^1^, ^2^, ^3^ Still, single-nucleotide polymorphisms (SNPs) do not explain the totality of observed phenotypic variability—a phenomenon referred to as “missing heritability”—and one proposed explanation is the contribution of additional types of genetic variants, such as copy-number variants (CNVs).^4^

Characterized by the deletion or duplication of DNA fragments ≥ 50 bases,^5^ CNVs represent a highly diverse mutational class that, due to their possibly large size, constitute potent phenotypic modifiers that act through e.g., gene dosage sensitivity, truncation or fusion of genes, unmasking of recessive alleles, or disruption of *cis*-regulatory elements.^6^ Hence, CNVs have been acknowledged to play an important role in human diseases and were identified as the genetic etiology of 65 rare and debilitating genomic syndromes by DECIPHER^7^ (web resources). However, early GWASs failed to establish clear links between CNVs and complex traits and diseases.^8^^,^^9^ Several factors, specific to genome-wide copy-number association studies (CNV-GWASs), contributed to these negative results, such as the low frequency and variable breakpoints of CNVs in the population, as well as uncertainty and low resolution of CNV calls originating from genotyping microarrays.^10^ In recent years, methodological development, as well as the creation of large biobanks, has allowed bypassing of some of these hurdles. Focusing on a curated set of CNVs, a series of studies characterized the impact of well-established pathogenic CNVs on cognitive performance,^11^ physical measurements,^12^^,^^13^ common medical conditions,^14^^,^^15^ and blood biomarkers.^16^ Alternatively, unbiased genome-wide (GW) studies have been conducted,^17^, ^18^, ^19^, ^20^, ^21^ involving loci not covered by targeted approaches and adding to the growing body of evidence implicating CNVs in complex traits. Notably, a recent study made use of the UK Biobank (UKBB)^22^ to assess the impact of CNVs on over 3,000 traits, providing the research community with a large population-based CNV-to-phenotype resource.^18^ Using an independent CNV calling and association pipeline and focusing on a set of 57 medically relevant continuous traits, we here confirm previously established associations, uncover biological insight through in-depth analysis of particular CNV-trait pairs, and expose a nuanced role of CNVs along the rare versus common disease spectrum, suggesting that the deleterious impact of CNVs contributes to decreased longevity in the general population.

## Material and methods

### Study material

#### Cohort description

Main analyses were performed in the UKBB, a volunteer-based cohort of ∼500,000 individuals (54% females) from the general UK population for which microarray-based genotyping and extensive phenotyping data are available.^22^ Participants signed a broad informed consent form and data were accessed through an application (16389) approved by the UKBB. Replication analyses were performed in the Estonian Biobank (EstBB), a population-based cohort of ∼200,000 individuals (66% females) for which microarray-based genotyping, body measurements, blood biomarker levels, and medical diagnoses are available.^23^ Whole-genome sequencing (WGS) data were available for ∼2,500 samples. All participants signed a broad informed consent form and analyses were carried out under ethical approval 1.1-12/624 from the Estonian Committee on Bioethics and Human Research and data release N05 from the EstBB. We used the Lausanne University Hospital (CHUV) maternity cohort, consisting of 5,164 women, to assess the impact of a Rhesus negative (Rh^−^) blood group on hematological traits. Approval from the Ethics Committee of Vaud (CER-VD) was obtained for data reusage (ID 2019-00280). Comprehensive cohort description is provided in supplemental material and methods, note 1.

#### Software versions

CNVs were called with PennCNV v1.0.5^24^ with PennCNV-Affy (27/08/2009). CNVs were filtered on the basis of a quality scoring pipeline.^25^ Various genetic analyses were conducted with PLINK v1.9 and PLINK v2.0.^26^ ANNOVAR (24/10/2019)^27^ was used for genome annotation. Meta-analysis was carried out with GWAMA v2.2.2.^28^ Statistical analyses were performed with R v3.6.1 and graphs were generated with R v4.0.3.

### The CNV landscape of the UK Biobank

#### Genotype data

Data acquisition and quality control (QC) have been described.^22^ Briefly, UKBB participants were genotyped on two similar arrays (95% probe overlap): 438,427 samples (95 batches) were genotyped with the Applied Biosystems UK Biobank Axiom Array (825,927 probes) and 49,950 samples (11 batches) were genotyped with the Applied Biosystems UK BiLEVE Axiom Array by Affymetrix (807,411 probes). All results in this study are based on the human genome reference build GRCh37/hg19.

#### Sample selection

Related, gender mismatched, high missingness, non-White British ancestry, and retracted samples were excluded (*used.in.pca.calculation* = 0 and *in.white.British.ancestry.subset* = 0 in Sample-QC v2 file). To protect the analysis from somatic chromosomal aberrations, we excluded individuals with self-reported (#20001, codes: 1047, 1048, 1050, 1051, 1052, 1053, 1055, 1056, 1056; UKBB update 03/2020) and/or hospital diagnosed (#41270; International Classification of Diseases, 10^th^ Revision [ICD-10] codes mapping to “cancer of lymphatic and hematopoietic tissue’s” exclusion range in the PheCode Map 1.2 [beta], accessed 09/12/2020;^29^ UKBB update 08/2019) blood malignancy. CNV outliers were later removed (CNV calling and quality control). All reported results are for 331,522 unrelated White British UKBB participants (54% females).

#### CNV calling and quality control

Chromosomes 1–22 and pseudoautosomal regions were assumed to have a normal copy-number state (i.e., two) in all individuals, and CNVs were called with standard PennCNV settings in parallel for all 106 genotyping batches. As males are hemizygous for chromosome X, chromosome X CNVs were called separately with the inbuilt PennCNV arguments for chromosome X CNV calling (supplemental material and methods, note 2). CNVs originating from samples genotyped on plates with a mean CNV count per sample > 100 or from samples with >200 CNVs or a single CNV > 10 Mb were excluded, as these might be indicative of batch effects, genotyping errors, or extreme chromosomal abnormalities. To mitigate issues related to high false positive rates and variability in CNV break points, we used a post-PennCNV processing pipeline^17^^,^^25^ (supplemental material and methods, note 2). First, a quality score (QS) ranging from −1 (likely deletion) to 1 (likely duplication) and reflecting the probability for the CNV to be a true positive was attributed to each PennCNV call. Next, PennCNV coordinates were transformed into per-chromosome probe×sample matrices; entries reflect the QS attributed to the CNV mapping to these probes. Copy neutral probes are indicated by 0 and individuals with no CNVs were added as all-0 columns.

#### Converting CNV calls into PLINK format

QS matrices were converted to PLINK binary file sets. Probes with ≥1 high-confidence CNV, stringently defined by |QS| ≥ 0.5, were retained and encoded into three file sets to accommodate analyses according to a mirror (PLINK_CNV_), duplication-only (PLINK_DUP_), or deletion-only (PLINK_DEL_) association model (--make-bed PLINK v1.9; Table 1).

**Table 1:** PLINK encoding of CNVs

**Association model**	**Mirror**	**Duplication-only**	**Deletion-only**
**PLINK file set**	**PLINK**_**CNV**_	**PLINK**_**DUP**_	**PLINK**_**DEL**_

Deletion (QS < −0.5)	AA	00	TT
Copy neutral (−0.5 ≤ QS ≤ 0.5)	AT	AT	AT
Duplication (QS > 0.5)	TT	TT	00

Encoding of high-confidence CNVs (|QS| ≥ 0.5) from quality score (QS) matrices into three PLINK file sets.

#### CNV frequency calculation

Genotype counting was performed for the 740,434 probes stored in PLINK_CNV_ (--freqx PLINK v1.9). 41,670 array-specific probes with genotype count missingness > 5% were excluded and each probe’s CNV $\;\left(100\times \;Num_{CNV}/(Num_{CNV}\;+\;Num_{non-CNV})\right)$, duplication $\left(100\times \;Num_{dup}/(Num_{CNV}\;+\;Num_{non-CNV})\right)$, and deletion $\left(100\times \;Num_{del}/(Num_{CNV}\;+\;Num_{non-CNV})\right)$ frequencies were calculated [%], with $Num_{non-CNV}$, $Num_{dup}$, and $Num_{del}$, the number of individuals carrying 2, <2, and >2 copies of that probe, respectively, and $Num_{CNV}=\;Num_{dup}+\;Num_{del}$.

### CNV association studies in the UK Biobank

#### CNV probe selection and number of effective tests

Association studies were restricted to probes with a CNV, duplication, or deletion frequency ≥ 0.005% for the mirror, duplication-only, or deletion-only models, respectively. To group probes at the core of CNV regions while retaining variability at breakpoints, we pruned probes at r^2^ > 0.9999 in PLINK_CNV_, PLINK_DUP_, and PLINK_DEL_ (--indep-pairwise 500 250 0.9999 PLINK v2.0). Retained CNV-proxy probes remained highly correlated and the number of effective tests, $N_{eff}$, was estimated at 11,804 (supplemental material and methods, note 3),^17^^,^^30^ setting the GW threshold for significance at p ≤ 0.05/11,804 = 4.2 × 10^−6^. Accounting solely for duplications or deletions resulted in lower $N_{eff}$ estimates but the same conservative threshold was used for all models.

#### Phenotype selection

Fifty-seven continuous traits were selected (supplemental material and methods, note 3). Traits were inverse normal transformed prior correction for sex (except for sex-specific traits), age (#21003), age^2^, genotyping batch, and principal components (PCs) 1–40. Normal phenotypic ranges were retrieved and converted from Symed MediCalc (web resources).

#### Genome-wide copy-number association studies

Associations between the copy number (CN) of selected probes and normalized covariate-corrected traits were performed (--glm omit-ref no-x-sex hide-covar allow-covars PLINK v2.0). To avoid interference between the two-letter CNV encoding (Table 1) and the assumption of male chromosome X hemizygosity, we (falsely) labeled all individuals as female. For sex-specific traits, samples from the opposite sex were excluded. Three association models were applied: the mirror model (PLINK_CNV_) assessed the additive effect of each additional copy of a probe, the duplication-only model (PLINK_DUP_) assessed the impact of a duplication while disregarding deletions, and the deletion-only model (PLINK_DEL_) assessed the impact of a deletion while disregarding duplications. Given CNV encoding (Table 1), effects were homogenized to “T” by multiplying β by −1 when A1 was “A.” GW-significant associations (p ≤ 4.2 × 10^−6^; CNV probe selection and number of effective tests) were retained. The number of independent signals per traits was determined by stepwise conditional analysis (supplemental material and methods, note 3). Briefly, CNV genotype of the lead probe was regressed out from the phenotype and association studies were conducted anew until no more GW-significantly associated probes remained.

#### CNV region definition, merging, and annotation

CNV region (CNVR) boundaries were defined by the most distant probe within ± 3 Mb and r^2^ ≥ 0.5 of each independent lead probe (--show-tags–tag-kb 3000 --tag-r2 0.5 PLINK v1.9). Signals from the different models were merged when involving (1) the same trait, (2) overlapping CNVRs, and (3) directional concordance according to a mirror model. CNVR boundaries were defined as the maximal CNVR and characteristics of the most significant model were retained. CNVRs were annotated with annotate_variation.pl, with hg19 RefSeq gene names (--geneanno; 08/06/2020) and NHGRI-EBI GWAS Catalog^31^ (web resources) associations (--regionanno; 27/10/2021) via ANNOVAR. NHGRI-EBI GWAS Catalog trait synonyms considered are listed in Table S1. For each trait, focusing on autosomes, we performed a two-sided binomial test to compare SNP-GWAS signal density (from NHGRI-EBI GWAS Catalog; 27/10/2021) within CNVRs as compared to the entire genome. Number of SNP-GWAS signals falling within trait-associated CNVRs represent successes, total length of trait-associated CNVRs [bp] represent trials, and total number of SNP-GWAS signals divided by the autosomal genome length (2,881,033,286 bp; web resources) represent hypothesized density.

### Replication in the Estonian Biobank

#### Comparative analysis of CNV quality

Quality-controlled Illumina Infinium OmniExpress-24 microarray genotyping and WGS data were available for 966 overlapping and unrelated samples of the EstBB (supplemental material and methods, note 4). Microarray-based autosomal CNVs were called with PennCNV and samples with >200 CNVs were excluded. PennCNV calls were attributed a QS and filtered for |QS| ≥ 0.5, following a procedure analogous to the one described for the UKBB. We called WGS-based autosomal CNVs by using the Genome STRiP pipeline^32^ and merged adjacent CNVs (gap ≤ 20% of merged CNV length) to mimic the PennCNV protocol. For both methods, we excluded duplications and deletions smaller than 1 kb and 2 kb, respectively, and larger than 10 Mb. For each of the 709,358 genotyped probes, a cross-sample PennCNV-CNV profile was constructed, taking values of −1 (deletion), 0 (copy neutral), and 1 (duplication). Similar profiles were constructed on the basis of STRiP-CNV calls and Pearson’s coefficient of correlation and number of CNV carriers according to both methods were calculated for each genomic location. For probes with ≥1 PennCNV call but no STRiP call, all correlated probes (r ≥ 0.5, according to PennCNV profiles) within ± 250 kb were retrieved and maximal PennCNV-WGS correlation among these probes was retained. Analyses were repeated on a subset of 5,566 probes overlapping UKBB-trait-associated CNVRs.

#### Phenotype data

Analyzed traits were queried in the EstBB: height, weight, and body mass index (BMI) were collected at enrollment; age at menarche and menopause were collected by project-based questionnaires; 41 traits were retrieved from parsed notes in health registries; 11 did not have any corresponding term. Because most phenotypic measurements originate from health registries, they were gathered at different time points and by different practitioners and were only available for a limited subset of participants. In case of repeated measurement, the most recent one was retained. Traits with sample size ≥ 2,000 were selected and inverse normal transformed prior correction for sex (except for sex-specific traits), age, age^2^, genotyping batch, and PCs 1–20.

#### CNV calling and copy-number association studies

We used quality-controlled Illumina Global Screening Array (GSA) genotype data (supplemental material and methods, note 4) to call autosomal CNVs for 193,844 individuals. CNVs were attributed a QS and encoded into three PLINK binary file sets, following the procedure described for the UKBB. CNV, duplication, and deletion frequencies among the 89,516 unrelated samples remaining after QC (supplemental material and methods, note 4) were calculated for 671,035 probes and association studies were run as previously described for the UKBB. Using the most significant association model for the 131 merged UKBB signals, we selected the most significantly associated EstBB probe within the boundaries of the UKBB-defined CNVR. EstBB p values were adjusted to account for directional concordance with UKBB effects: in case of direction agreement, ${\text{p}}_{new}=\;\frac{{\text{p}}_{old}}{2}$, otherwise ${\text{p}}_{new}=\;1-\left(\frac{{\text{p}}_{old}}{2}\right)$. Sufficient genomic variability and phenotypic data were available to assess replication of 61 out of 131 signals, setting the replication threshold for significance at p ≤ 0.05/61 = 8.2 × 10^−4^. We conducted simulations to estimate the power of our replication study assuming effect sizes similar to those observed in the UKBB and CNV frequencies and sample sizes reflective of the EstBB (supplemental material and methods, note 4). For each signal, 10,000 simulations were conducted. Power was defined as the fraction of non-missing p values ≤ 8.2 × 10^−4^. Expected number of replications was estimated as the average power across assessed signals multiplied by the number of assessed signals.

### Extended phenotypic assessment

To assess patients’ disease status, ICD-10 codes were used (#41270). Self-reported high alcohol consumption (#1558) and γ-glutamyl transferase (GGT)-increasing drug usage (#20003) were evaluated as potential lifestyle confounders of the 22q11.23-GGT association. Six socio-economic factors and life history traits were additionally considered in the burden analysis. Traits were inverse normal transformed prior to correction for sex, age (#21003), age^2^, genotyping batch, and PCs 1–40, except for “age at recruitment”, which was not corrected for age and age^2^. Exact definitions are found in supplemental material and methods, note 5.

### *RHD* and hematological traits

#### Transcriptome-wide Mendelian randomization

Using univariable transcriptome-wide Mendelian randomization^33^ (TWMR), we estimated the causal effects of differential *RHD* and *RSRP1* expression on reticulocyte count, platelet count, and glycated hemoglobin (HbA1c; supplemental material and methods, note 6). Robustness was ascertained by excluding rs55794721, which had an extreme effect on both exposures and outcomes.

#### Association between Rh blood group and hematological traits

Impact of Rh^−^ blood group on platelet count, reticulocyte count, and HbA1c levels was assessed in the CHUV maternity cohort through multivariate linear regression that incorporates the covariates: age at measurement, gestational week at measurement, whether the woman was pregnant at measurement, and whether the woman had a child prior to the measurement (supplemental material and methods, note 6). One-sided p values were calculated as ${\text{p}}_{new}=\frac{{\text{p}}_{old}}{2}$ in case of directional agreement with the UKBB effect.

### CNV burden analyses in the UK Biobank

#### CNV burden calculation

An individual’s CNV burden was defined as the number of Mb or genes affected by high-confidence autosomal CNVs (|QS| ≥ 0.5). For the latter, we retained CNVs overlapping exons, splice sites, non-coding RNA, 3′UTR, and 5′UTR (CNV region definition and annotation) to assess number of disrupted genes. Duplication and deletion burdens were calculated similarly, and correlation between the six metrices was assessed with Pearson’s coefficient of correlation. We used two-sided unpaired Wilcoxon rank-sum test to assess differences in CNV burden between males and females.

#### CNV burden analysis

Linear regressions were performed between burden metrices and the same 57 normalized, covariate-corrected traits investigated by GWAS. For sex-specific traits, samples from the opposite sex were excluded. We set the significance threshold at p ≤ 0.05/63 = 7.9 × 10^−4^ to account for six additional life history traits (supplemental material and methods, note 5). Linear regressions were computed between non-normalized, covariate-corrected “mother’s and father’s age at death” and the burden to get effects on the years/[Mb or gene] scale. We meta-analyzed results with GWAMA to assess impact on parental lifespan.

#### Burden analysis correction for modifier CNVRs

To assess the impact of the CNV burden on a trait, we collected CNVRs associating with that trait under the mirror model into a sample×CNVRmatrix G. G Takes a value of −1 or 1 if the sample carries a CNVR-overlapping (≥1 bp) deletion or duplication, respectively, and 0 otherwise. G was regressed out of the trait and burdens were adjusted by subtracting the number of Mb or genes affected by CNVR-overlapping CNVs before performing associations anew. For the duplication and deletion burdens, CNVRs found through the duplication-only and deletion-only models, respectively, were considered and CNVR-overlapping deletions and duplications, respectively, were set to 0 in G.

#### Fraction of inherited CNVs

Rate of CNV inheritance was estimated by examining the fraction of shared CNVs among siblings pairs defined by kinship coefficient 0.2–0.3 and fraction of SNPs with identity by state at 0 ≥ 0.0012.^22^ We retained 16,179 pairs with one individual among samples selected for the main CNV-GWASs (Sample selection). Shared CNVs were defined as high-confidence duplications (QS ≥ 0.5) or deletions (QS ≤ −0.5) on the same chromosome with ≥25 kb overlap. For each pair, we calculated the fraction of CNVs the individual in the main analysis shared with his/her sibling (number of shared CNVs/total number of CNVs in that individual) and averaged the results over all pairs to obtain the mean fraction of shared CNVs. As a control, the analysis was repeated by pairing the 16,179 individuals from the main analysis with random individuals sampled without replacement from the main pool of individuals.

## Results

### The CNV landscape of the UK Biobank

We used PennCNV^24^ to call autosomal, pseudoautosomal, and chromosome X CNVs in 332,935 unrelated White British UKBB participants with no reported blood malignancy. Calls were processed by a pipeline that excluded 1,413 CNV outlier samples and attributed a probabilistic QS to each CNV.^25^ Out of 1,329,290 identified CNVs, 176,870 high-confidence CNVs with |QS| ≥ 0.5 were retained for follow-up analyses (Figure S1A). As the fraction of homozygous CNVs (CN 0 or 4) was negligeable (1.1%; Figure S1B), we define deletions and duplications as having a CN smaller or larger than two, respectively, for the remainder of this study. Duplication length varied between 366 bp and the upper boundary, set at 10 Mb (17–3,968 probes), with a median of 297 kb (133 probes), and deletion length between 217 bp and 10 Mb (8–4,017 probes), with a median of 137 kb (60 probes) (Figures S1C and S1D). Overall, 129,263 (39%) participants carried at least one high-confidence CNV and 34,804 (10%) carried more than one (Figure S1E). In samples with ≥1 CNV, the total length of affected bases ranged between 217 bp and 14.2 Mb, with a median of 292 kb (Figure S1F). Analyzing the global CNV burden of the cohort, 70% was caused by duplications, which were both more numerous (54%) and 213 kb longer, on average, than deletions (Figures S1B–S1D). No differences in CNV burden, measured as the number of Mb or genes affected by CNVs, was detected across sexes (two-sided, unpaired Wilcoxon rank-sum test: p_*Mb*_ = 0.793; p_*Genes*_ = 0.748). This contrasts with the excess of deleterious CNVs reported in females with neuro-psychiatric/developmental disorders,^34^, ^35^, ^36^, ^37^ suggesting that this observation is trait dependent.

**Figure 1:**
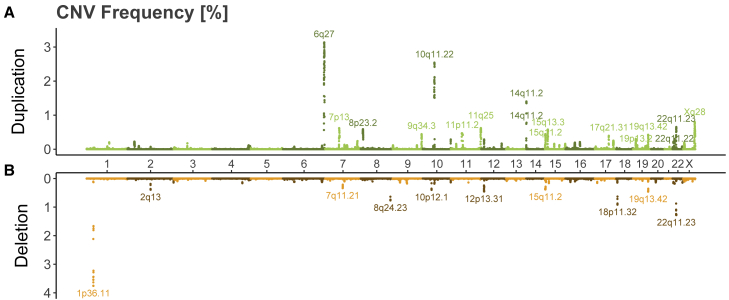
CNV frequency landscape in the UK Biobank (A and B) Miami plot of high-confidence probe-level duplication (A) and deletion (B) frequencies [%] in the UKBB. Consecutive probes with identical duplication and deletion frequencies were clustered so that each dot represents one probe group. Loci with duplication frequency ≥ 0.3% or deletion frequency ≥ 0.2% are labeled with cytogenic bands.

To bypass issues related to inter-individual variability in recurrent CNV break points, we transformed CNV calls to the probe level for frequency calculation.^17^ A large fraction of the genome was subjected to CNVs as 662,247 probes (82%) were found in a CN-altered state in at least one participant, even if 81% of these had a CNV frequency ≤ 0.005% (n ≤ 16). The fraction of never-deleted probes (43%) was 1.7× higher than the fraction of never-duplicated probes (26%), and with some notable exceptions, deletion frequencies tended to be lower than duplication frequencies (Figure 1). For most loci with high CNV frequency, duplication and deletion frequencies did not mirror each other (Figure 1). Overall, these results are in line with the common paradigm that CNVs are individually rare but collectively common.^18^^,^^38^^,^^39^

### The pleiotropic impact of recurrent CNVs

**Figure 2:**
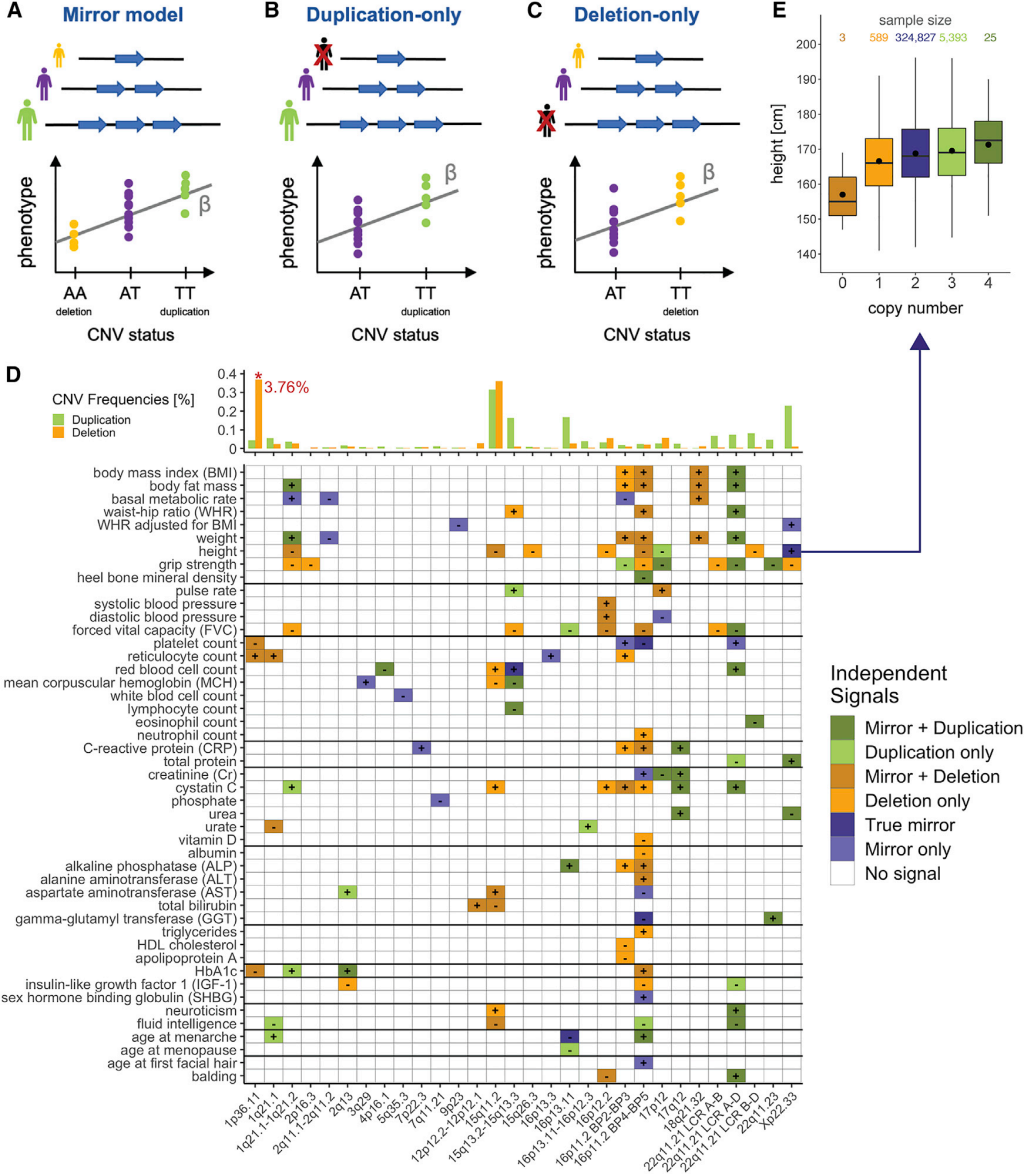
CNV-GWAS roadmap of the UK Biobank (A–C) CNV-GWAS association models with PLINK encoding: the mirror model assumes equal-sized but opposite-direction effect of deletion and duplication and estimates the impact of each additional copy (A); the duplication-only model disregards deletion carriers and estimates the effect of duplications (B); the deletion-only model disregards duplication carriers and estimates the effect of deletions (C). (D) Independent genome-wide significant associations (p ≤ 0.05/11,804 = 4.2 × 10^−6^) between CNV regions (x axis; as cytogenic bands) and traits (y axis). Color tiles represent the model(s) through which the association was detected—dark green, mirror and duplication-only; light green, duplication-only; dark orange, mirror and deletion-only; light orange, deletion-only; dark purple, mirror, duplication-only, and deletion-only; light purple, mirror; white: none—and signs show directionality, so that the duplication (greens), deletion (oranges), or copy number (purples) of a CNV region associated with a phenotypic increase (+) or decrease (−). 16p11.2 (16p11.2 BP2-BP3 and 16p11.2 BP4-BP5) and 22q11.21 recurrent CNVs (LCR B at chr22: 20,400,000) are assessed separately. For each CNV region, average duplication (green) and deletion (orange) frequencies [%] of the lead probe (according to the most significant model) are depicted at the top. Deletion frequency of 1p36.11 was truncated from 3.76%. (E) Boxplot representing height in individuals with CNVs overlapping the Xp22.33 pseudoautosomal region (chrX: 285,850–1,720,422). Sample size is reported for each copy-number category at the top; boxes show the first (Q1), second (median, thick line), and third (Q3) quartiles; lower and upper whiskers show the most extreme value within Q1 minus and Q3 plus 1.5× the interquartile range, respectively; dots show the mean; outliers are not shown.

To assess the phenotypic impact of the UKBB CNV landscape, we selected 57 medically relevant phenotypes—including anthropometric traits, cardio-pulmonary assessments, hematological measurements, blood biomarkers, neuronal functions, and sex-specific attributes—with presumed high heritability (Table S1; Figure S2A). GWASs were performed between the CN of pruned (r^2^ > 0.9999) CNV-proxy probes with a CNV, duplication, and deletion frequency ≥ 0.005% and aforementioned traits according to a mirror (28,257 probes; Figure 2A), duplication-only (14,070 probes; Figure 2B), and deletion-only (9,936 probes; Figure 2C) association model, respectively. As the number of statistical tests is much lower than for classical SNP-GWASs and retained probes remain highly correlated due to the recurrent nature and large size of assessed CNVs, we calculated the number of effective (i.e., independent) tests, setting the GW threshold for significance at p ≤ 0.05/11,804 = 4.2 × 10^−6^ (material and methods). Stepwise conditional analysis narrowed signals down to 86, 50, and 68 GW-significant associations for the mirror, duplication-only, and deletion-only models, respectively, of which 45, 22, and 32 reached the conventional SNP-GWAS threshold of p ≤ 5 × 10^−8^. These signals were combined into 131 independent associations spanning 47 phenotypes (Figure 2D; Table S2; 62 signals across 32 phenotypes at p ≤ 5 × 10^−8^). Following previous works,^17^^,^^18^^,^^21^ we omitted accounting for the number of assessed traits, but even with a stringent experiment-wide threshold for significance (p ≤ 0.05/(11,804 × 57) = 7.4 × 10^−8^), 68 out of 131 (52%) CNV-GWAS signals remained significantly associated. All summary statistics are made available (data and code availability).

Among signals identified through the mirror model, 63 (73%) replicated with either type-specific model, often reflecting the most common CNV type (Figure 2D, top). Five (6%) signals replicated with both type-specific models, providing examples of “true mirror” effects (i.e., opposite impact of duplications and deletions), such as the association between height and the CN of a Xp22.33 pseudoautosomal CNVR (chrX: 285,850–1,720,422; β_mirror_ = 2.33 cm; p = 7.2 × 10^−36^; Figure 2E) encompassing the short-stature homeobox gene *SHOX* (MIM: 312865). This association aligns with the short stature observed in individuals suffering from Turner syndrome (i.e., females with partial or complete loss of one chromosome X) and *SHOX* deficiency disorders (Leri-Weill dyschondrosteosis [MIM: 127300]; Langer mesomelic dysplasia [MIM: 249700]; idiopathic short stature [X-linked] [MIM: 300582]).^40^^,^^41^ Less established is the impact of increased CN of *SHOX* and/or its regulatory regions,^42^ which we found to be associated with tall stature. CN and deletion of overlapping CNVRs further associated with waist-to-hip ratio (WHR) adjusted for BMI (chrX: 514,930–618,611; β_mirror_ = 0.12 SD; p = 2.3 × 10^−6^) and hand grip strength (chrX: 762,346–2,219,659; β_del_ = −4.73 kg; p = 3.7 × 10^−7^), respectively. While skeletal muscle hypertrophy has been reported in patients with Leri-Weill dyschondrosteosis,^43^ we hypothesize that the reduced grip strength in deletion carriers might result from the Madelung deformity characterizing the disorder, which is known to cause wrist pain and decreased grip strength,^44^ and/or the correlation between grip strength and height (Figure S2A). Unlike mirror effects, partially overlapping signals between decreased forced vital capacity or grip strength and the 22q11.21 low copy repeat (LCR) A-B (chr22: 19,024,651–20,311,646; deletion-only) and 22q11.21 LCR A-D (chr22: 19,024,651–21,407,523; mirror and duplication-only) hinted at U-shaped effects (i.e., deletion and duplication shift the phenotype in the same direction) (MIM: 188400 and 192430), demonstrating the existence of different mechanisms of gene dosage (Figure 2D).

Most signals involved large recurrent CNVRs (mean = 901 kb; median = 612 kb) and we confirm multiple well-established associations, such as the negative impact of the 1q21.1–1q21.2 deletion (MIM: 612474) on height^45^, ^46^, ^47^ (chr1: 146,478,785–147,832,715; β_del_ = −6.67 cm; p = 2.5 × 10^−21^), the negative correlation between BMI and the CN of 16p11.2 BP4-BP5 (MIM: 611913 and 614671) (chr16: 29,596,230–30,208,637; β_del_ = 6.11 kg/m^2^; p = 3.6 × 10^−29^)^48^, ^49^, ^50^ and 16p11.2 BP2-BP3 (MIM: 613444) (chr16: 28,818,541–29,043,450; β_del_ = 4.25 kg/m^2^; p = 5.3 × 10^−8^),^49^^,^^51^^,^^52^ or the more recently reported positive association between 16p11.2 BP4-BP5’s CN and age at menarche (chr16: 29,596,230–30,208,637; β_mirror_ = 1.16 years; p = 1.2 × 10^−10^).^53^ In addition, our results revealed the broad pleiotropic impact of these loci: 26, 16, and 12 traits associated with the 16p11.2 BP4-BP5, 22q11.21, or 16p11.2 BP2-BP3 regions, respectively. Some of these previously poorly described associations might help shed light on the molecular mechanisms linking involved loci to phenotypes, as exemplified with the association between the 16p11.2 BP4-BP5 deletion (chr16: 29,596,230–30,208,637) and reduced levels of insulin-like growth factor 1 (IGF-1; β_del_ = −3.26 nmol/L; p = 2.9 × 10^−7^). In children, diseases characterized by low levels of IGF-1 (e.g., IGF-1 deficiency [MIM: 608747], Laron syndrome [MIM: 262500], or growth hormone [GH] deficiencies [MIM: 262400, 612781, 173100, 307200, 618157, and 615925]) typically result in short stature (proxied by height), while symptoms of adult GH deficiency include increased adipose mass (proxied by BMI, body fat mass, weight, and WHR), decreased muscle mass and strength (proxied by grip strength), altered lipid profile (proxied by triglycerides), and insulin resistance (proxied by HbA1c),^54^ all of which are affected in a directionally concordant fashion by the 16p11.2 BP4-BP5 deletion. Conversely, some regions only associated with a single trait, e.g., the CN of a 3q29 region (chr3: 195,725,157–196,035,229) associated with increased mean corpuscular hemoglobin (MCH; β_mirror_ = 1.92 pg; p = 1.1 × 10^−9^), whose levels indirectly reflect iron load in erythrocytes.^55^ The CNVR harbors the transferrin receptor gene, *TFRC* (MIM: 190010), which is involved in cellular iron uptake and was shown to associate with MCH through SNP-GWAS.^56^ Together, these results emphasize the potent role of CNVs as phenotypic modifiers.

### Replication in the Estonian Biobank

We next assessed our ability to detect CNVs and sought to replicate identified signals in an independent cohort, the EstBB.^23^ Taking advantage of 966 unrelated samples with both microarray-based (PennCNV) and WGS-based (STRiP) CNV calls, we calculated the correlation between the CNV profiles obtained with these two methods for 709,358 quality-controlled, autosomal probes (Figure S3A). Due to small sample size, most probes (630,819 probes; 89%) were monomorphic. Among the 20,963 probes detected in a CNV state in at least one sample by both methods, 71% (14,976 probes; 2.1% of all probes) showed high (r ≥ 0.75) agreement in calling profiles. We detected 39,847 (5.6%) apparent false positives (i.e., probes only detected in a CNV state by PennCNV). Forty percent of these were in linkage disequilibrium (± 250 kb and r ≥ 0.5) with probes showing high microarray-WGS concordance (Figure S3B), suggesting that they are true positives mislabeled as false positives due to fragmentation of STRiP CNV calls. We also observed 17,717 (2.5%) false negatives (i.e., probes only detected in a CNV state by STRiP). Size distribution—both in number of base pairs (Figure S3C) and probes (Figure S3D)—of consecutive stretches of false negative probes was smaller than for the other assessed categories, confirming the poor ability to detect small CNVs with microarray data.^10^ If false negatives hinder discovery, they do not affect validity of detected associations. We next repeated the analysis on 5,566 probes overlapping UKBB trait-associated CNVRs (Figure S3E) and observed (1) an increased fraction of highly correlated probes (1,431 probes; 71% → 85%), (2) an increased fraction of apparently mislabeled false positives in linkage disequilibrium with highly correlated probes (1,061 probes; 40% → 92%; Figure S3F), and (3) a decreased proportion of false negatives among non-monomorphic probes (215 probes; 23% → 7%), indicating good sensitivity and specificity to detect CNVs at trait-associated genomic loci.

**Figure 3:**
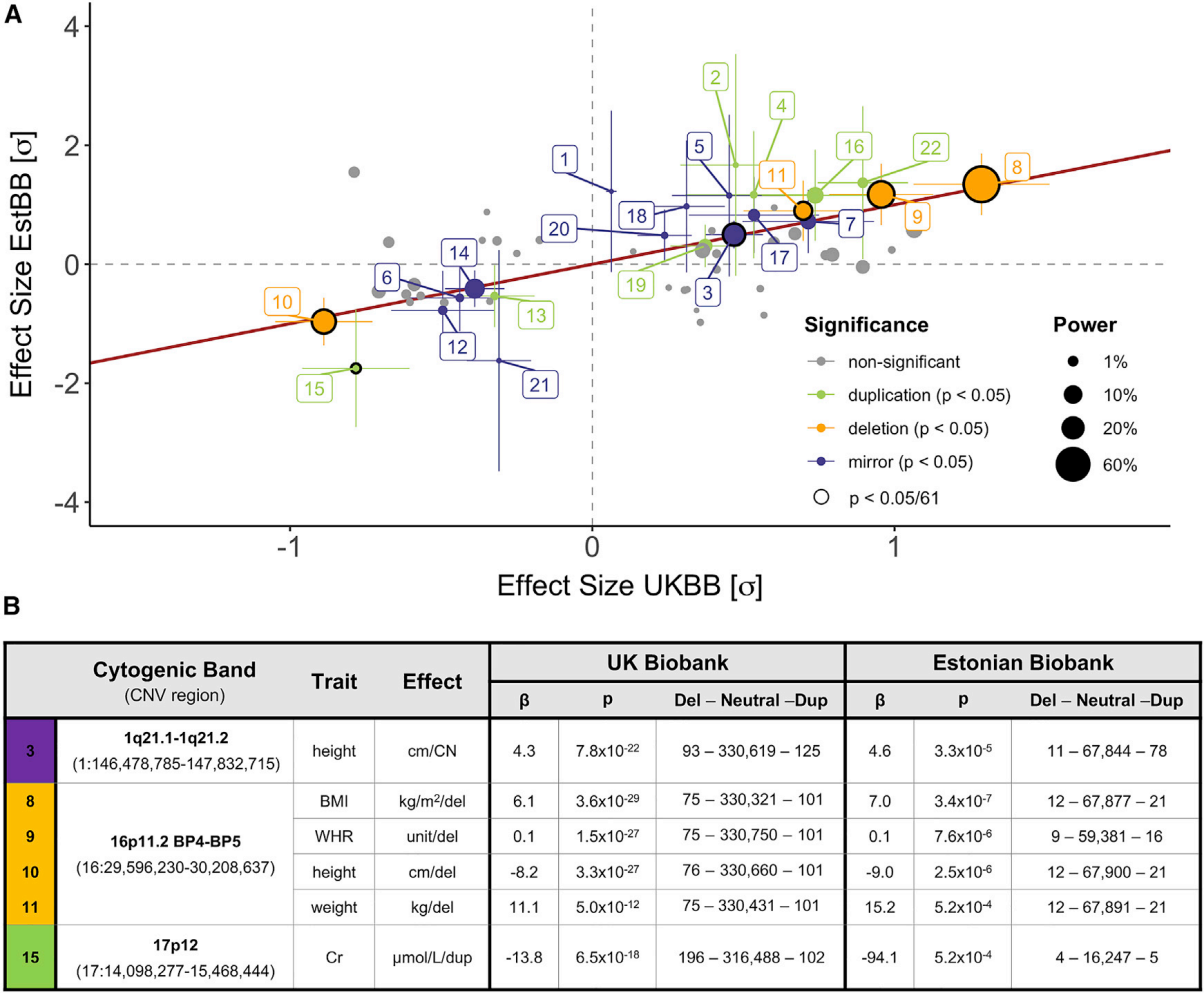
Replication of CNV-GWAS signals in the Estonian Biobank (A) Estonian Biobank (EstBB; y axis) versus UK Biobank (UKBB; x axis) standardized effect sizes. The identity line is in red; size reflects power at α = 0.05/61; non-significant signals (p > 0.05) are in gray; nominally significant signals (p ≤ 0.05) with 95% confidence intervals are colored according to replication models: mirror (purple), duplication-only (green), or deletion-only (orange); multiple-testing correction surviving signals (p ≤ 8.2 × 10^−4^) are circled in black and listed in (B) with the first column’s color corresponding to the association model and numbers matching labels in (A). (B) Effect sizes (β; unit in the effect column) and p values (p) for the UKBB and EstBB GWAS, along with the number of individuals with available phenotypic data carrying a deletion, no CNV, or a duplication overlapping the CNV region. Labels indicate: (1) platelet count—1p36.11 (chr1: 25,599,041–25,648,747); (2) glycated hemoglobin (HbA1c)—1q21.1–1q21.2 (chr1: 146,478,785–147,832,715); (3) height—1q21.1–1q21.2 (chr1: 146,478,785–147,832,715); (4) age at menarche—1q21.1 (chr1: 145,368,664–145,738,611); (5) platelet count—16p11.2 BP2-BP3 (chr16: 28,818,541–29,043,450); (6) weight—16p11.2 BP2-BP3 (chr16: 28,818,541–29,043,450); (7) age at menarche—16p11.2 BP4-BP5 (chr16: 29,596,230–30,208,637); (8) body mass index (BMI)—16p11.2 BP4-BP5 (chr16: 29,596,230–30,208,637); (9) waist-to-hip ratio (WHR)—16p11.2 BP4-BP5 (chr16: 29,596,230–30,208,637); (10) height—16p11.2 BP4-BP5 (chr16: 29,596,230–30,208,637); (11) weight—16p11.2 BP4-BP5 (chr16: 29,596,230–30,208,637); (12) alanine aminotransferase (ALT)—16p11.2 BP4-BP5 (chr16: 29,624,931–30,208,637); (13) age at menopause—16p13.11 (chr16: 15,151,451–16,308,285); (14) age at menarche—16p13.11 (chr16: 15,120,501–16,308,285); (15) serum creatinine (SCr)—17p12 (chr17: 14,098,277–15,468,444); (16) SCr—17q12 (chr17: 34,797,651–36,249,489); (17) C-reactive protein (CRP)—17q12 (chr17: 34,797,651–36,249,489); (18) platelet count—22q11.21 LCR A-D (chr22: 19,024,651–21,174,444); (19) BMI—22q11.21 LCR A-D (chr22: 19,024,651–21,463,515); (20) weight—22q11.21 LCR A-D (chr22: 19,024,651–21,463,545); (21) eosinophil count—22q11.21 LCR B-D (chr22: 20,457,855–21,463,545); (22) γ-glutamyl transferase (GGT)—22q11.23 (chr22: 23,688,345–24,990,213).

To replicate association signals, microarray-based CNV data were available for 89,516 unrelated individuals. Phenotypic measurements, originating from national health registries, were only available for a limited subset of participants, ranging from ∼60,000 for anthropometric measurements, to <1,000 for specialized biomarkers (Table S1). Restricting ourselves to autosomal signals with sample size ≥2,000 and ≥1 CNV carrier, data were available for 61 (47%) CNVR-trait pairs (Table S2; Figure 3A). Six signals replicated with Bonferroni correction for multiple testing (p ≤ 0.05/61 = 8.2 × 10^−4^; Figure 3B) and we observed 7.2× more nominally significant signals than expected by chance (22 signals; two-sided binomial test: p = 7.8 × 10^−14^; Figure S3G). Effect size estimates followed closely the ones detected in the UKBB (Figure 3). Given the low sample sizes, we conducted simulations to assess the power of the replication study. Assuming effect sizes matching those observed in the UKBB, the average replication power was estimated at 5.5% (α = 0.05/61; Figure S3H). This corresponds to an expected number of replicated signals of 3.4, slightly below the six observed, and argues in favor of the robustness of the original UKBB CNV-GWAS findings.

### CNVs as modifiers of complex traits

To assess whether CNV-GWAS signals mapped to regions previously identified by SNP-GWASs for the same trait, we annotated CNVRs with associations reported by the NHGRI-EBI GWAS Catalog.^31^ From the 126 autosomal CNV associations considered, 48 (38%) harbored a SNP signal for the same trait (Table S2). A similar fraction (31%) of CNV-GWAS signals with 4.2 × 10^−6^ ≥ p ≥ 5 × 10^−8^ is supported by SNP-GWAS signal, backing the reliability of intermediate-significant associations. We further tested whether SNP-GWAS signal distribution was denser within trait-associated CNVRs, as compared to the rest of the genome. While this was the case for nine traits (two-sided binomial test: p ≤ 0.05/56 = 8.9 × 10^−4^; Table S3), enrichment did not seem to depend on the type of trait, total number of SNP-GWAS signals (Figure S2B), or length of trait-associated CNVRs (Figure S2B, insert). Nevertheless, colocalization of SNP and CNV signals reinforces confidence that involved loci play a role in shaping associated traits, as illustrated with four examples.

The first example relates to a 1.7 Mb 2q13 CNV (chr2: 111,398,266–113,115,598). Deletion of the region associated with decreased IGF-1 (β_del_ = −5.67 nmol/L; p = 6.3 × 10^−10^), an important regulator of glucose and insulin metabolism,^57^ and duplication associated with increased HbA1c (β_dup_ = 3.47 mmol/mol; p = 1.4 × 10^−7^). The interval encompassed an IGF-1-associated intronic *ACOXL* SNP^20^ upstream of *BCL2L11* (MIM: 603827) and two HbA1c-associated SNPs^20^^,^^58^ downstream of *BCL2L11*. These SNP signals were reported in 2021, indicating that with increased statistical power, signal colocalization will increase. Both traits have not been thoroughly assessed in carriers of the recurrent reciprocal 2q13 CNV, who present with neuro-developmental/psychiatric disorders, dysmorphisms, congenital heart disorder, hypotonia, seizures, micro-/macrocephaly, and microphallus at variable penetrance and expressivity;^59^, ^60^, ^61^, ^62^, ^63^ the two latter features are reminiscent of growth defects potentially mediated by dysregulation of the GH/IGF-1/insulin axis. Multiple genes overlapping the CNVR play a role in cell cycle (*BUB1* [MIM: 602452], *ANAPC1* [MIM: 608473]), cell survival (*MERTK* [MIM: 604705]), and apoptosis (*BCL2L11*) regulation; *BCL2L11* is negatively regulated by IGF-1.^64^ Our data support the variable penetrance and expressivity of this CNV—not listed as a DECIPHER CNV syndrome—and prompts follow-up studies to confirm and refine understanding of the genetic mechanisms linking the locus to the associated phenotypes.

**Figure 4:**
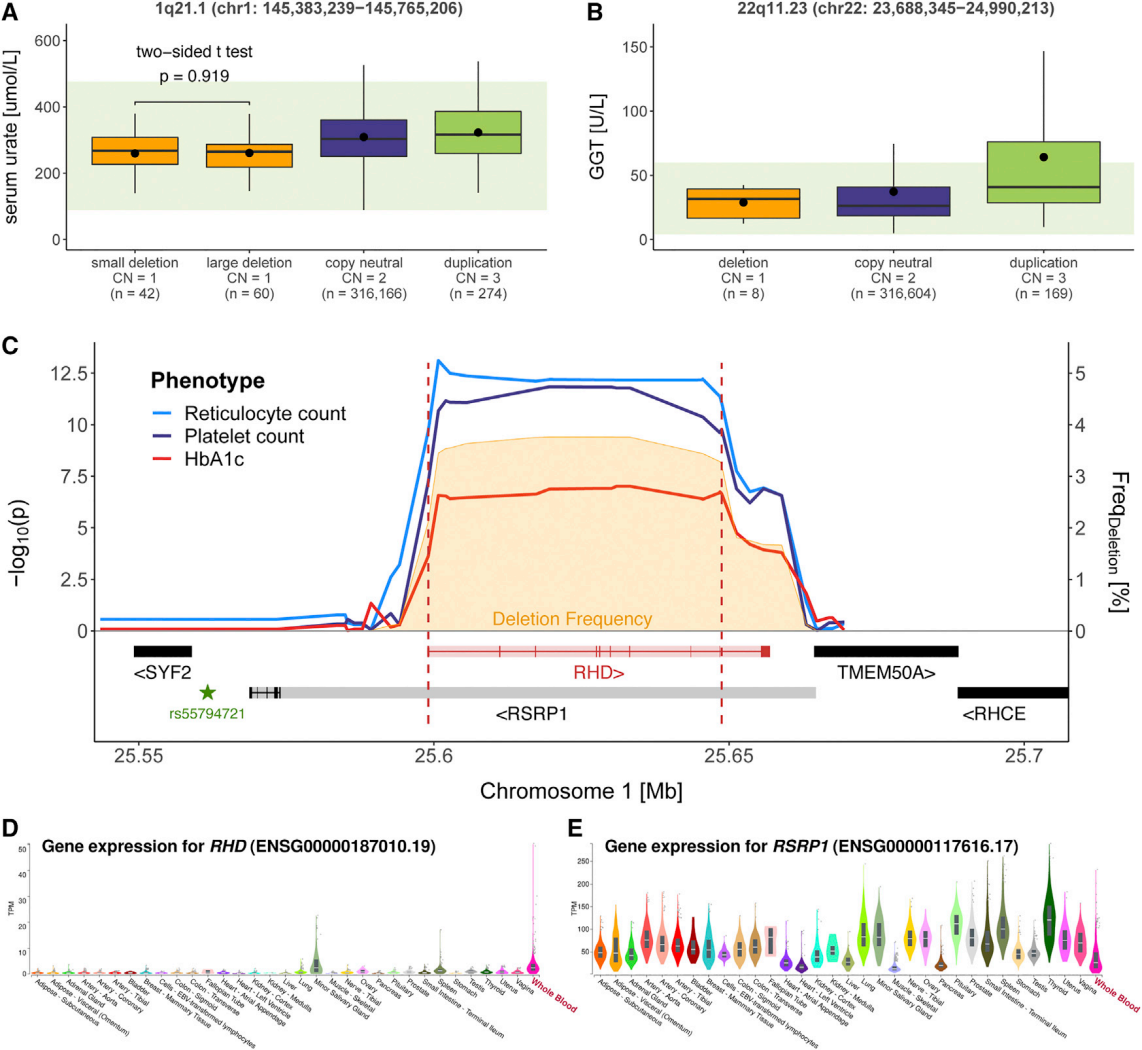
CNV-GWAS associations at SNP-GWAS loci (A and B) Boxplots representing levels of (A) serum urate in individuals with a 1q21.1 (chr1: 145,383,239–145,765,206) overlapping small (start ≥ 145.6 Mb) or large (start < 145.6 Mb) deletion, copy-neutrality, or duplication and (B) γ-glutamyl transferase (GGT) in individuals with a 22q11.23 (chr22: 23,688,345–24,990,213) overlapping deletion, copy-neutrality, or duplication. Copy number (CN) and sample size (n) are reported for each category; boxes show the first (Q1), second (median, thick line), and third (Q3) quartiles; lower and upper whiskers show the most extreme value within Q1 minus and Q3 plus 1.5× the interquartile range, respectively; dots show the mean; outliers are not shown; light green backgrounds show normal clinical range for serum urate: 89–476 mmol/L (A) and GGT: 4–6 U/L (B). p value of a two-sided t test comparing serum urate levels of small and large 1q21.1 deletion carriers is shown. (C) Association plot for the 1p36.11 deletion (chr1: 25,599,041–25,648,747). Red dashed lines delimit the deletion-only CNV region; left y axis shows the negative logarithm of association p value for reticulocyte count (blue), platelet count (purple), and glycated hemoglobin (HbA1c; red); right y axis shows deletion frequency [%] (orange); encompassed genes are schematically represented at the bottom; retained exons for the most strongly expressed isoform in whole blood are shown for *RHD* (ENST00000328664) and *RSRP1* (ENST00000243189), and shaded color represents the full gene sequence; star indicates the *RHD* and *RSRP1* expression quantitative locus rs55794721. (D and E) GTEx v8 gene expression in 33 tissues for *RHD* (D) and *RSRP1* (E). Brain, cervix, esophagus, and skin are not shown for visibility. Whole blood is shown with a red label.

The second example links the 382 kb 1q21.1 deletion (MIM: 274000) to decreased serum urate levels (chr1: 145,383,239–145,765,206; β_del_ = −48.32 μmol/L; p = 5.8 × 10^−13^; Figure 4A). The rearranged interval encompasses 15 genes (Figure S4), including *PDZK1* (MIM: 603831), which encodes a urate transporter scaffold protein^65^ and was associated with serum urate levels by SNP-GWASs.^66^, ^67^, ^68^, ^69^ Recently, *in vitro* experiments elucidated the mechanism through which the urate-increasing T allele of rs1967017 leads to increased *PDZK1* expression,^70^ while the *PDZK1* protein-truncating variant rs191362962 was found to associate with decreased serum urate,^20^ both suggesting that decreased *PDZK1* expression—an expected outcome of *PDZK1* deletion—decreases serum urate levels. Dividing deletion carriers into groups harboring a full (start < 145.6 Mb) or a partial (start ≥ 145.6 Mb) deletion revealed that the small deletion, encompassing *PDZK1* and three other genes (Figure S4), was sufficient to alter serum urate levels (two-sided t test: p = 0.92; Figure 4A).

The third example involves a 1.3 Mb long 22q11.23 duplication and increased GGT (chr22: 23,688,345–24,990,213; β_dup_ = 37.2 U/L; p = 9.3 × 10^−32^; Figure 4B). The region harbors several independent GGT SNP-GWAS signals^20^^,^^71^, ^72^, ^73^, ^74^, ^75^, ^76^ (MIM: 612365) and five genes involved in glutathione metabolism (KEGG pathway hsa00480), including *GGT1* (MIM: 612346) and *GGT5* (MIM: 137168) (Figure S5A), suggesting that an additional copy of these genes associates with increased levels of the encoded protein. As multiple factors can elevate GGT levels,^77^ we used binomial tests to verify that the 180 duplication carriers were not enriched for GGT-altering drug usage (p = 0.55), high alcohol consumption (p = 0.85), heart failure (p = 0.23), or cancer (p = 1) and other diseases (p = 0.64) of the liver, gallbladder, and bile ducts, as compared to control individuals. Visualization of GGT levels in individuals with two or three copies of the CNVR showed that the 22q11.23 duplication increased serum GGT independently of and additively to other GGT-increasing factors (Figures S5B–S5F).

Finally, we focused on the most frequent CNV in our cohort (frequency = 3.76%; Figure 1A), the 50 kb 1p36.11 deletion (chr1: 25,599,041–25,648,747), which encompasses *RHD* (Rhesus [Rh] blood group D antigen [MIM: 111680]) and *RSRP1* and associated with increased reticulocyte count (β_del_ = 2.7 × 10^9^ cells/L; p = 7.8 × 10^−14^), decreased platelet count (β_del_ = −3.7 × 10^9^ cells/L; p = 1.4 × 10^−12^), and decreased HbA1c (β_del_ = −0.3 mmol/mol; p = 9.3 × 10^−8^) (Figure 4C). Overlap with SNP-GWAS signals for various hematological traits^78^^,^^79^ combined with subsequent replication of the reticulocyte count association based on whole-exome sequencing CNV calls^80^ prompted the investigation of the expression of these genes in whole blood. Tissue-specific transcriptomic data from the GTEx project v8^81^ (web resources) revealed that *RHD*, a protein whose presence/absence on erythrocyte cell membranes is critical in determining an individual’s Rh blood group,^82^ was almost exclusively expressed in whole blood (Figure 4D), whereas *RSRP1* was ubiquitously expressed, with lower expression in whole blood (Figure 4E). Selecting *RHD*’s (ENST00000328664) and *RSRP1*’s (ENST00000243189; Figure S6A) most highly expressed isoforms in whole blood, we mapped exons to the association plot, showing that *RSRP1*’s isoform does not overlap the CNVR, in contrast to *RHD*’s, which is fully encompassed by it (Figure 4C). We next used transcriptome-wide Mendelian randomization^33^ (TWMR; Table S4) to establish a directionally concordant causal link between *RHD* expression and reticulocyte count (α_TWMR_ = −0.013, p = 1.6 × 10^−4^; Figure S6B), platelet count (α_TWMR_ = 0.031, p = 2.3 × 10^−9^; Figure S6C), and HbA1c levels (α_TWMR_ = 0.017, p = 3.5 × 10^−7^; Figure S6D). *RSRP1* TWMR resulted in directionally concordant and significant effects, but the gene had suboptimal number of instruments (three) for robust causal inference (Figures S6E–S6G). Furthermore, both genes’ signals were driven by a strong upstream expression quantitative locus (rs55794721; Figures S6B–S6G). Strengthening the causal role of *RHD*’s CN, lack or strongly reduced expression of all Rh antigens, a rare condition named Rh deficiency or Rh_null_ syndrome [MIM: 617970 and 268150], is associated with increased erythrocyte osmotic fragility, resulting in hemolytic anemia.^83^ Hemolytic anemia is characterized by increased reticulocyte count^84^ and can falsely lower HbA1c levels because of decreased erythrocyte lifespan,^85^ putting forward the hypothesis that heterozygous deletion of *RHD* leads to subclinical phenotypes akin to hemolytic anemia. To gauge the generalizability of these results, we looked for similar trends in individuals with Rh^−^ blood type, which can be caused by various polymorphisms.^82^ Because Rhesus groups were unavailable for the UKBB, we turned to a maternity cohort from the Lausanne University Hospital. Despite low samples sizes, concordant trends of increased reticulocyte count (β_*Rh−*_ = 1.07°/oo; p_*one-sided*_ = 0.134; n = 741) and decreased platelet count (β_*Rh−*_ = −2.8 × 10^9^ cells/L; p_*one-sided*_ = 0.126; n = 5,034) and HbA1c levels (β_*Rh−*_ = −0.22%; p_*one-sided*_ = 0.050; n = 418) were observed in Rh^−^ women (Table S5). Of note, reticulocyte and platelet counts have been reported to increase and decrease, respectively, along pregnancy,^86^ and despite correcting for pregnancy status and gestational weeks, interaction between Rh^−^ blood group and pregnancy cannot be excluded. Impact of Rh blood type on hematological traits awaits validation but these examples illustrate how studying CNVs at SNP-GWAS loci can pinpoint causal genes and shared genetic mechanisms.

### CNVs at Mendelian disorder loci

**Figure 5:**
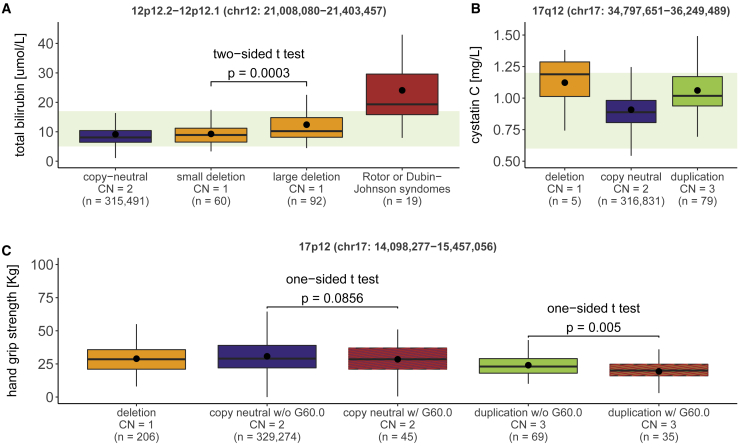
CNV-GWAS associations at Mendelian disorder loci (A–C) Boxplots showing total bilirubin levels in copy-neutral individuals, small (start ≥ 21.1 Mb) or large (start < 21.1 Mb) 12p12.2-p12.1 (chr12: 21,008,080–21,403,457) overlapping deletion carriers, and Rotor or Dubin-Johnson syndrome-affected individuals (ICD-10 E80.6) (A), cystatin C levels in individuals with a 17q12 (chr17: 34,797,651–36,249,489) overlapping deletion, copy-neutrality, or duplication (B), and hand grip strength in individuals with a 17p12 (chr17: 14,098,277–15,457,056) overlapping deletion, copy-neutrality, or duplication, split according to the presence (w/) or absence (w/o) of a neuropathy (ICD-10 G60.0; red stripes) (C). Copy number (CN) and sample size (n) are reported for each category; boxes show the first (Q1), second (median, thick line), and third (Q3) quartiles; lower and upper whiskers show the most extreme value within Q1 minus and Q3 plus 1.5× the interquartile range, respectively; dots show the mean; outliers are not shown; light green backgrounds show normal clinical range for total bilirubin: 5–17 mmol/L (A) and cystatin C: 0.6–1.2 mg/L (B). p value of a two-sided t test comparing total bilirubin levels of small and large 12p12.2-p12.1 deletion carriers is shown. p values of one-sided t tests comparing hand grip strength of copy neutral and 17p12 duplication carriers with or without a neuropathy diagnosis are shown.

Despite the lower-than-average disease burden of UKBB participants,^87^ several associations comprised loci involved in Mendelian disorders. The heterozygous 395 kb 12p12.2-p12.1 deletion, which associated with a non-pathological increase in total bilirubin (chr12: 21,008,080–21,403,457; β_del_ = 3.1 μmol/L, p = 2.2 × 10^−13^; Figure 5A) and harbors SNP-GWAS signals for bilirubin levels,^20^^,^^88^, ^89^, ^90^, ^91^, ^92^ overlaps the Rotor syndrome locus (MIM: 237450), an extremely rare disorder whose main clinical manifestation is hyperbilirubinemia. Rotor syndrome^93^ is caused by the homozygous disruption of *SLCO1B1* (MIM: 604843) and *SLCO1B3* (MIM: 605495) (Figure S7), which encode for the hepatic transporters OATP1B1 and OATP1B3, respectively, involved in the uptake of various drugs and metabolic compounds, including bilirubin.^94^ Concordantly, UKBB participants diagnosed with Rotor syndrome or the related and more common Dubin-Johnson syndrome (MIM: 237500) presented above-normal levels of total bilirubin (Figure 5A). Interestingly, individuals carrying a partial deletion that only affects *SLCO1B1* (start ≥ 21.1 Mb; Figure S7) exhibited significantly milder increase in total bilirubin (two-sided t test: p = 3.1 × 10^−4^; Figure 5A), illustrating how mutations pathogenic in a digenic recessive framework can contribute to subtle changes in disease-associated phenotypes when present in an isolated heterozygous state.

A second example links the 1.5 Mb long 17q12 duplication (MIM: 614526) (chr17: 34,797,651–36,249,489) and increased levels of kidney damage biomarkers, including cystatin C (β_dup_ = 0.15 mg/L, p = 4.2 × 10^−17^; Figure 5B), serum creatinine (SCr; β_dup_ = 13.0 μmol/L, p = 2.7 × 10^−16^; Figure S8A), and serum urea (β_dup_ = 0.93 mmol/L, p = 9.1 × 10^−10^; Figure S8B), as well as the inflammation biomarker C-reactive protein (CRP; β_mirror_ = 2.3 mg/L, p = 1.1 × 10^−6^; Figure S8C). Deletion of this interval (Figure S8D), as well as point mutations in overlapping *HNF1B* (MIM: 189907), cause the highly pathogenic and penetrant autosomal dominant renal cysts and diabetes syndrome (RCAD [MIM: 137920 and 614527]). RCAD is characterized by heterogenous structural and/or functional renal defects, neuro-developmental/psychiatric disorders, and maturity-onset diabetes of the young (see GeneReviews by Mitchel et al. in web resources). Because of the small number of deletion carriers (n = 6, regardless of phenotypic data availability), the deletion’s effect was not assessed by CNV-GWASs, but elevated levels of cystatin C (Figure 5B), SCr (Figure S8A), and urea (Figure S8B) in these individuals align with RCAD’s clinical description. Conversely, penetrance of the reciprocal duplication remains debated and only ∼20% of diagnosed patients report renal abnormalities (see GeneReviews by Mefford in web resources). In line with a lower pathogenicity, we detected 16× more duplication than deletion carriers. Still, these individuals showed strong alterations in kidney biomarkers (Figure 5B; Figure S8), suggesting tight gene dosage control on *HNF1B*.

Third, we zoomed in on the 1.4 Mb long 17p12 duplication (Figure S9A) known as the main etiology of Charcot-Marie-Tooth (CMT) type 1A (MIM: 118220), a demyelinating neuropathy of the peripheral nervous system characterized by progressive muscle wasting.^95^ Correspondingly, duplication carriers showed decreased hand grip strength (chr17: 14,098,277–15,457,056; β_dup_ = −9.8 kg, p = 4.1 × 10^−39^; Figure 5C) and lower SCr (chr17: 14,098,277–15,468,444; β_dup_ = −13.8 μmol/L, p = 6.5 × 10^−18^; Figure S9B; EstBB: β_dup_ = −94.1 μmol/L, p = 5.2 × 10^−4^; Figure 3), indicating decreased muscle mass.^96^ We next assessed the proportion of duplication carriers (regardless of phenotypic data availability) diagnosed with CMT or related hereditary motor and sensory neuropathies and detected 48 and 38 diagnoses among the 331,206 copy-neutral individuals and 107 duplication carriers, respectively. While there is a clear enrichment for CMT diagnoses among duplication carriers (Fisher’s exact test: odds ratio = 3,668, p < 2.2 × 10^−16^), only 36% of duplication carriers were clinically identified. To test whether these individuals presented with more extreme clinical manifestations, we compared grip strength and SCr levels in duplication carriers with or without a neuropathy diagnosis. The former group exhibited lower grip strength (one-sided t test: p = 0.005; Figure 5C) but no difference was detected in SCr levels (one-sided t test: p = 0.384; Figure S9B). Importantly, there was no age difference between diagnosed (mean = 55.5 years) and undiagnosed (mean = 56.2 years) duplication carriers (two-sided t test: p = 0.650), indicating that results do not reflect biases regarding age of disease onset.

These examples show that well-established pathogenic CNVs can modulate disease-associated phenotypes in the general population without necessarily causing clinically diagnosable disorders, supporting a model of variable expressivity for the involved loci.^97^, ^98^, ^99^, ^100^

### CNV-GWAS signals suggest gene functionalities

**Figure 6:**
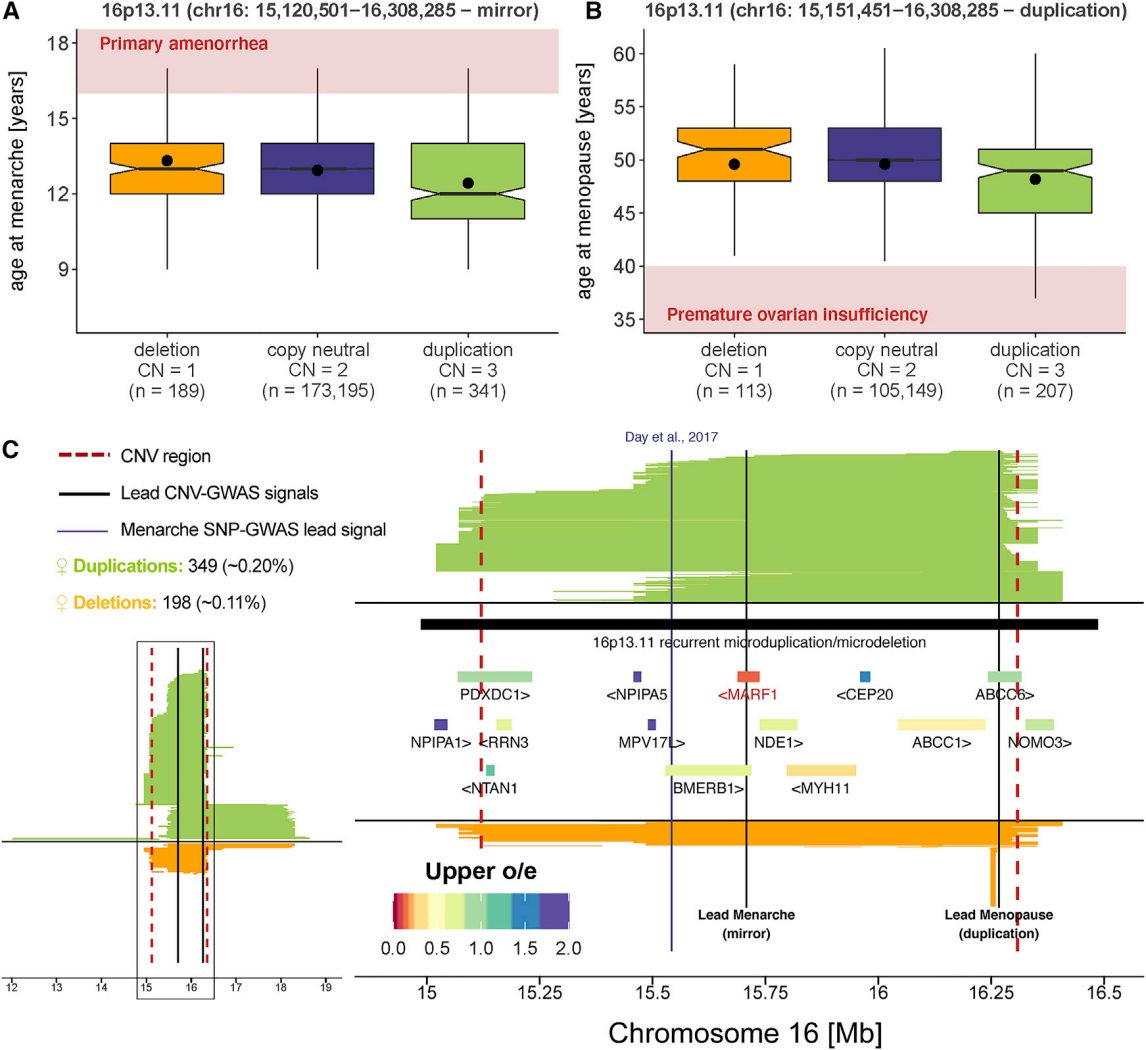
*MARF1* as a putative gene involved in human female reproduction (A and B) Boxplots representing age at menarche (A) and menopause (B) in individuals with a 16p13.11 (A, chr16: 15,120,501–16,308,285; B, chr16: 15,151,451–16,308,285) overlapping deletion, copy-neutrality, or duplication. Copy number (CN) and sample size (n) are reported for each category; dots show the mean; boxes show the first (Q1), second (median, thick line), and third (Q3) quartiles; lower and upper whiskers show the most extreme value within Q1 minus and Q3 plus 1.5× the interquartile range, respectively; notches represent median ± 1.58 × IQR/√n; outliers are not shown; light red backgrounds indicate pathogenic values corresponding to primary amenorrhea (age at menarche > 16 years) (A)^109^ and premature ovarian insufficiency (age at menopause < 40 years), respectively (B).^110^ (C) Mapping of CNVs overlapping the 16p13.11 CNV region (chr16: 15,120,501–16,308,285). Number and frequency of duplications and deletions are at the top left; left plot shows all overlapping CNVs; right plot focuses on the associated CNV region delineated with red dashed lines; duplications are in green, deletions in orange; black lines indicate the lead signal for age at menarche (mirror) and menopause (duplication-only); purple line indicates age at menarche-associated SNP;^101^ overlapping recurrent DECIPHER CNV is shown in black and protein-coding genes are colored according to the upper bound of the confidence interval for the observed/expected (o/e) mutation ratio in gnomAD.

CNV-GWAS signals can corroborate or generate hypotheses regarding the function of encompassed genes, as shown with the association between the CN of a 1.2 Mb 16p13.11 interval and female reproductive traits. Specifically, duplication of the region correlated with decreased age at menarche (chr16: 15,120,501–16,308,285; β_dup_ = −0.6 years, p = 2.0 × 10^−10^) and menopause (chr16: 15,151,451–16,308,285; β_dup_ = −1.8 years, p = 1.7 × 10^−6^), whereas its deletion correlated with increased age at menarche (chr16: 15,120,501–16,308,285; β_del_ = 1.1 years, p = 3.6 × 10^−7^), suggesting a shift in reproductive timing associated with the region’s CN (Figures 6A and 6B) that aligns with a low, albeit positive, genetic correlation between the two traits (Neale Lab UKBB genetic correlation; web resources). Duplication effect on age at menarche (β_dup_ = −0.6 years, p = 1.8 × 10^−2^) and menopause (β_dup_ = −2.6 years, p = 4.5 × 10^−2^) were confirmed with nominal significance in the EstBB (Figure 3A) and a SNP-GWAS signal for age at menarche (rs153793) colocalized with the CNVR^101^ (Figure 6C). Literature supports the role of *MARF1* (MIM: 614593) in this association. First, *MARF1* (observed/expected ratio [o/e] = 0.05 [0.03–0.12]; probability of loss-of-function intolerance [pLI] = 1) and *MYH11* (o/e = 0.22 [0.16–0.30]; pLI = 0.77; [MIM: 160745]) are the only encompassed genes under evolutionary constraint according to gnomAD^102^ (upper bound of o/e <0.35; Figure 6C; Table S6; web resources). Second, *MARF1* was shown to play an essential role in murine oogenesis by fostering successful completion of meiosis and cytoplasmic maturation and protecting germline genomic integrity.^103^ The gene’s function is supported by studies in fly^104^ and goat,^105^ as well as two human case reports of females with *MARF1* mutations and reproduction phenotypes.^106^^,^^107^ The female-specific role of *MARF1*^103^, ^104^, ^105^, ^106^, ^107^, ^108^ aligns with the absence of association with our proxies for male sexual maturation (i.e., age at first facial hair and balding). Although further investigations are warranted to characterize the function of *MARF1* in human female reproduction and assess the contribution of nearby genes and regulatory elements, it illustrates how CNV-GWASs can be leveraged to generate plausible hypotheses regarding gene functionalities.

### The deleterious impact of a high CNV burden

**Figure 7:**
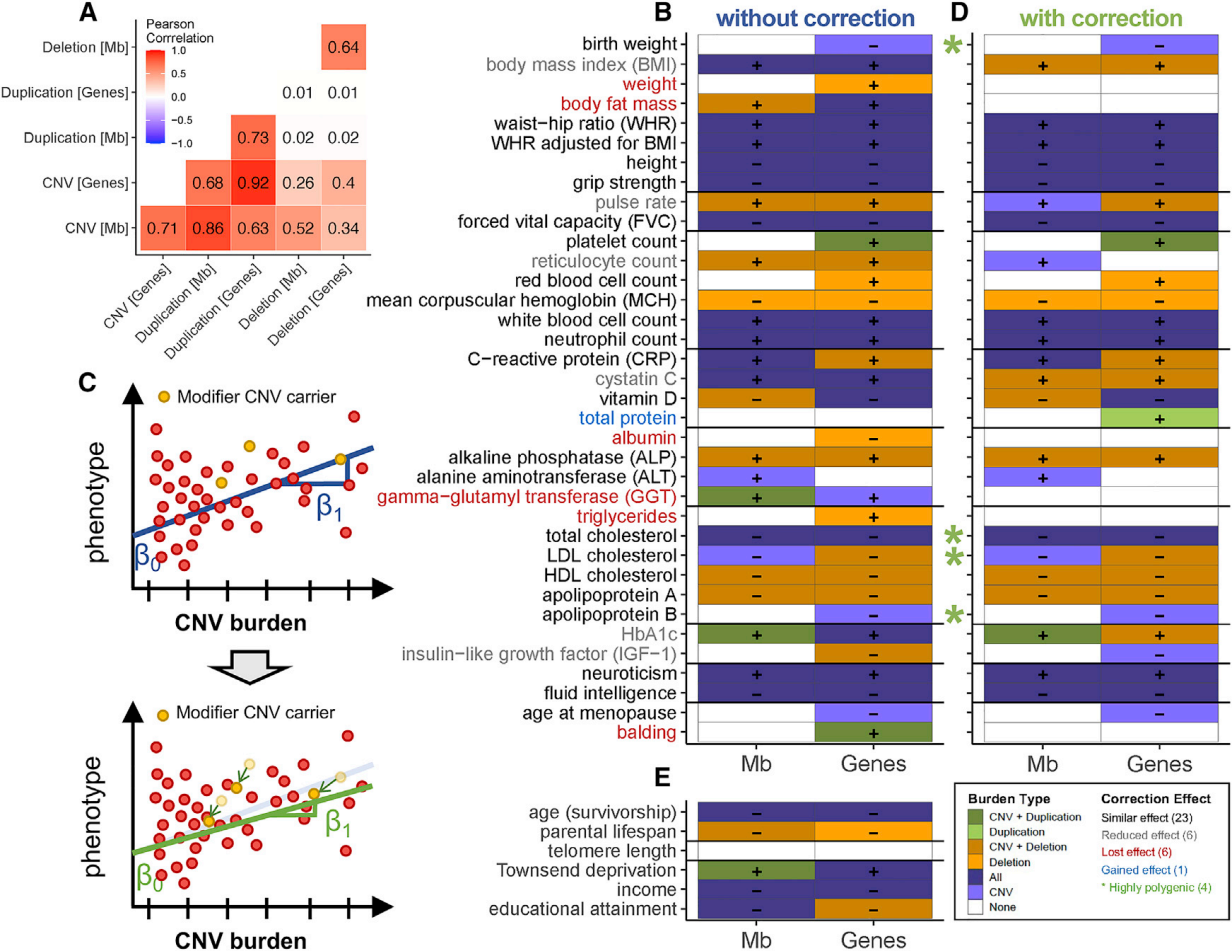
The negative impact of the CNV burden on complex traits (A) Pearson correlation across six burden metrices. (B) Significant associations (p ≤ 0.05/63 = 7.9 × 10^−4^) between the CNV burden, expressed as the number of Mb or genes affected by CNVs (x axis), and traits assessed through CNV-GWASs (y axis). Color represents the type of burden—dark green, CNV and duplication-only; light green, duplication-only; dark orange, CNV and deletion-only; light orange, deletion-only; dark purple, CNV, duplication-only, and deletion-only; light purple, CNV; white, none—found to increase (+) or decrease (−) the considered phenotype. (C) Schematic representation of the correction for modifier CNVs. Top: individuals carrying a CNV overlapping a CNV-GWAS region were identified (i.e., modifier CNV carrier; yellow). Bottom: Phenotype and burden were corrected (green arrows) and a new linear regression was fitted. (D) Significant associations (p ≤ 0.05/63 = 7.9 × 10^−4^) between the CNV burden after correction for modifier CNVs. Phenotype label color indicates whether the number of associated metrices between the CNV burden and the trait was fully lost (0 associations; red), decreased (gray), identical (black), or increased (blue) after the correction. Green stars mark highly polygenic traits associating with the CNV burden without having any significant CNV-GWAS signals. (E) Significant associations (p ≤ 0.05/63 = 7.9 × 10^−4^) between the CNV burden and life history traits (y axis). (D and E) follow the legend in (B).

Moving beyond single CNVs, the impact of an individual’s total CNV burden on complex traits was estimated. Each participant’s autosomal CNV, duplication, and deletion burden was calculated in number of affected Mb or genes. Both Mb and gene burden metrices correlated well (ρ: 0.71–0.74) and while we observed high correlations (ρ*:* 0.40–0.92) between the CNV and duplication/deletion burdens, the two latter were uncorrelated (Figure 7A). From the 57 traits analyzed by CNV-GWASs, 35 (61%) significantly associated with at least one burden metric (p ≤ 0.05/63 = 7.9 × 10^−4^, material and methods), showcasing negative health consequences such as increased levels of adiposity, liver/kidney damage biomarkers, leukocytes, glycemic values, or anxiety and decreased global physical capacity or intelligence (Figure 7B; Table S7). Harmful phenotypic consequences were often best captured by the number of deleted genes, in line with a higher sensitivity to decreased (i.e., haploinsufficiency) rather than increased (i.e., triplosensitivity) gene dosage.^111^

We then corrected each individual’s phenotype and burden for the presence of trait-associated CNVs and performed the burden analysis anew to ensure that signals were not solely driven by significantly trait-associated CNVs (Figure 7C; Table S7). Whereas the association was lost for albumin, balding, body fat mass, GGT, triglycerides, and weight, indicating a mono- or oligogenic CNV architecture, 30 traits remained associated. Among these, birth weight, total cholesterol, low-density lipoprotein (LDL) cholesterol, and apolipoprotein B (ApoB) were significantly associated with the burden (Figure 7D) but lacked CNVR associations (Figure 2D). This indicates that, as established for SNPs,^1^, ^2^, ^3^ the CNV architecture underlying most complex traits is polygenic, suggesting the presence of additional associations that we currently lack the power to detect.

The CNV burden extended its impact to global aspects of an individual’s life, as illustrated by the negative correlation with several socio-economic factors, including decreased educational attainment (EA; β_burden_ = −0.07 years/Mb, p = 4.4 × 10^−11^) and income (β_burden_ = −1,593 £/year/Mb, p = 2.9 × 10^−60^), and increased Townsend deprivation index (β_burden_ = 0.04 SD/Mb, p = 3.6 × 10^−7^) (Figure 7E; Table S8). While we did not observe any effect of the CNV burden on age- and sex-corrected telomere length, the trait specifically associated with the *BRCA1* cancer locus^112^ (MIM: 113705) (chr17: 41,197,733–41,258,551, β_dup_ = 0.45 SD, p = 1.9 × 10^−8^), paralleling findings that long telomere-associated SNPs also associate with increased cancer risk.^113^ Because of the low number of deceased UKBB participants, we used proxies to assess the impact of the CNV burden on lifespan; we observed a negative association between an individual’s CNV burden and both parental lifespan (β_burden_ = −0.21 years/Mb, p = 1.4 × 10^−5^) and age (survivorship proxy; β_burden_ = −0.18 years/Mb, p = 1.1 × 10^−7^), suggesting that the deleterious impact of CNVs contributes to decreased longevity (Figure 7E; Table S8). Given this, we questioned whether the CNV burden was transmitted at a Mendelian rate. Taking advantage of the presence of a UKBB sibling for 16,179 individuals assessed in our previous analyses, we calculated that the average fraction of shared CNVs among siblings was 27%. Whereas substantially higher than for random pairs (0.7%), it only represents 54% of the expected fraction of shared additive genetic variance among siblings (50%).^114^ Together, these results describe the broadly deleterious impact of CNVs on a wide range of complex traits in the general population and suggests that most traits are influenced by a polygenic CNV architecture.

## Discussion

By coupling CNV calls to the phenotypic data available in the UKBB, we generated a roadmap of clinically relevant CNV-trait associations that allowed us to gain deeper insights into specific biological pathways and put forward general patterns describing the role of CNVs in shaping complex human traits in the general population.

Our UKBB CNV landscape matched previous reports,^18^ and while some of the 131 CNV-GWAS signals overlapped known associations,^12^^,^^17^^,^^18^^,^^20^ our analyses shed light on others that have not been studied extensively. Combined use of three association models revealed general patterns through which CNVs modulate phenotypes, and while geared toward the discovery of mirror effects, we also witnessed U-shape effects, illustrating different mechanisms through which altered dosage influences phenotypes. We further provide evidence for a broad and nuanced role of CNVs in shaping complex traits, as both common (frequency ≥ 1%) and rare (frequency < 1%) CNVs mapping to regions involved by SNP-GWAS contribute to phenotypic variability in the general population, and rare CNVs have larger effects sizes than common ones. Other signals mapped to regions involved in Mendelian disorders. Studying pathogenic CNVs in the general population, as opposed to clinical cohorts selected on the basis of phenotypic criteria or family history, makes it possible to re-assess their frequency, penetrance, expressivity, pleiotropy, and inheritance pattern. Matching the increasing awareness around variable penetrance and expressivity,^97^, ^98^, ^99^, ^100^^,^^115^^,^^116^ we show that pathogenic dominant CNVs can impact disease-associated traits without causing clinically diagnosable disorders, whereas recessive CNVs can impact disease-related biomarkers at the heterozygous state. Together, these results provide a more complex and nuanced—but also broader—understanding of the phenotypic impact of CNVs at odds with the classical dichotomy between common complex diseases and rare Mendelian disorders.

Confirming the deleterious influence of a high CNV load on anthropometric traits^17^^,^^117^^,^^118^ and EA^11^^,^^119^^,^^120^ in a non-clinical cohort, we extended this observation to over 30 global health biomarkers. We show how the CNV burden—limited to large and rare CNVs detectable by microarrays—shapes intermediate molecular phenotypes that predate or are consequences of disease processes in a population-based cohort, consistent with its known contribution to a wide range of disorders.^121^, ^122^, ^123^, ^124^, ^125^ Our data further show that the CNV load negatively impacts socio-economic factors and longevity proxies. The lower CNV burden observed in individuals with advanced age matches the depletion of life-shortening alleles in older UKBB participants,^126^ suggesting improved health/decreased mortality in individuals with a low CNV load. Parental lifespan negatively correlated with the CNV burden. While lower than expected, a substantial fraction of CNVs (27%) was shared among siblings and thus inherited from either parent. As inclusion of haplotype sharing information in CNV calling mainly increases the detection of small (<10 kb) but not that of large CNVs,^21^ we hypothesize that large events recurrently appear *de novo* on multiple backgrounds and are rapidly eliminated from the population through transmission bias or from the cohort through ascertainment bias (i.e., increased participation of healthier siblings) because of their deleteriousness. Our analysis of CNV call quality in the EstBB suggests marginal contribution of false CNV calls but confounders–such as CNV length, which affects both detection capacity and pathogenicity–prevent the assessment of these factors separately. Nevertheless, the lower-than-expected CNV inheritance allow speculating that an even stronger association with lifespan would be obtained providing access to parental CNV genotypes. If further studies are required to confirm the life-shortening effect of a high CNV load, our data clearly illustrate the deleterious impact of CNVs on an individual’s global health.

Both CNV-GWASs and burden analyses results improve the understanding of the CNV architecture underlying studied traits. Many CNV-GWAS loci involve rare but recurrent CNVRs. Due to the difficulty of gathering large cohorts of carriers, complete phenotypic characterization of these loci is still missing and limited to easily assessed anthropometric traits or severely debilitating neuro-developmental/psychiatric disorders. Our results provide a map of the pleiotropic consequences of these CNVRs on over 50 medically relevant traits. Some traits are not typically assessed/reported in patient cohorts and targeted study of their distribution among cases might refine diagnostic criteria and help clinicians identify and follow-up on patients with mild and/or atypical presentation. Mechanistically, most assessed CNVRs are large, potentially harboring several causal genes. One of the next challenges will be to narrow down causal region(s) in pleiotropic multi-genic CNVRs to untangle primary from secondary associated traits, as some, such as obesity, are known to causally alter multiple biomarkers.^127^, ^128^, ^129^ The substantial overlap between CNV- and SNP-GWAS signals speaks for the presence of shared genetic mechanisms, so that both mutational classes can be exploited synergistically to pinpoint causal genes and elucidate their biological function. In parallel, we observed a high degree of CNV-polygenicity, as 30 out of 35 traits remained associated with the CNV burden after correction for modifier CNVRs. For six traits, CNV-GWAS signals captured the bulk of phenotypic variability caused by CNVs, while ApoB, birth weight, LDL cholesterol, and total cholesterol were solely associated with the CNV burden. This indicates a polygenic CNV architecture that might arise from rare high impact CNVs that were not assessed by CNV-GWASs (frequency ≤ 0.005%) and/or more frequent CNVs with mild effects; indeed, most high frequency CNVRs do not overlap CNV-GWAS signals (Figures 1 and 2D). Among these, decreased birth weight, which associated with a high CNV load, has been linked to increased risk for metabolic syndrome, obesity, and various other diseases in adulthood,^130^^,^^131^ opening the question as to whether some of the deleterious effects of the CNV burden are rooted in early development. Strikingly, the three other traits are plasma lipids with few CNV-GWAS signals. Speaking for their high polygenicity, a GWAS on 35 blood biomarkers in the UKBB found an average of 87 versus 478 associations per trait for non-lipid compared to lipid traits.^20^ Collectively, these results illustrate a more complex than expected contribution of CNVs in shaping the genetic architecture of complex human traits.

It is important to keep in mind limitations of the current study. First, CNVs were called on the basis of microarray data with PennCNV. In addition to high false positive rates associated to array-based CNV calls, this renders the study blind to variants in regions not covered by the array, limits resolution—both in length and exact break point location—and hinders the detection of high copy-number states (CN ≥ 4) and deviations thereof. To mitigate these issues, we stringently filtered CNVs and transformed calls to the probe level,^17^^,^^25^ which at risk of missing true associations guarantees the identification of trustworthy CNV-trait pairs. Few cohorts have sufficiently large genetic and phenotypic coverage to replicate UKBB findings at adequate power, so that we relied on literature evidence to gauge the validity of our results, highlighting the need for large-sized biobanks for studying (rare) CNVs. Future release of large sequencing datasets combined to progress in CNV detection tools could resolve these issues and lead to novel discoveries.^21^^,^^80^^,^^132^^,^^133^ Second, despite substantial evidence of CNV- and SNP-GWAS signal colocalization, we did not perform robust enrichment analyses, as the non-random genomic distribution and complex nature of CNVs renders simulating the null scenario beyond the scope of this paper. Signal colocalization is likely to be underestimated, as manual literature searches revealed overlaps missed by our annotation pipeline (e.g., 16p13.11 age at menarche signal^101^) and we obtained a 7% increase in signal colocalization by using GWAS Catalog annotation 6 months apart (31% April 2021 → 38% October 2021). Third, our study is limited to individuals of White British ancestry. As CNV frequencies vary across populations,^5^^,^^134^, ^135^, ^136^ assessing diverse ancestral groups is likely to unravel new associations, even though smaller sample sizes represent a limiting factor. Finally, the UKBB suffers from a “healthy cohort” bias.^87^ Focusing on the impact of CNVs in healthy populations, we used this bias to our advantage through the inclusion of CNV carriers with sub-clinical phenotypes, providing lower bounds for effect size estimates.^99^^,^^100^^,^^137^ However, this means that the cohort is depleted for severely affected cases and extremely rare (frequency ≤ 0.005%) but highly pathogenic CNVs were not tested for associations. Extending the analysis to low frequency/high impact CNVRs would allow for better distinguishing of mechanisms of action—with the remaining caveat that effects will be underestimated because of selection bias—and will be the focus of future work.

In conclusion, our study provides a map of high-confidence CNV-trait associations. While we explored some of the reported signals, collective efforts will be required to validate and interpret these discoveries and we hope that this resource will be useful for researchers and clinicians aiming at improving the characterization of recurrent CNVs. Our study revealed the nuanced role of CNVs along the rare versus common disease spectrum, their shared mechanisms with SNPs, as well as a widespread polygenic CNV architecture, consolidating the growing body of evidence implicating CNVs in the shaping of complex human traits.

## Consortia

The members of the Estonian Biobank Research Team are Tõnu Esko, Andres Metspalu, Lili Milani, Reedik Mägi, and Mari Nelis.

## Data and code availability

Code is available on GitHub at https://github.com/cauwerx/CNV_GWAS_continuous_traits. Probe-level CNV frequencies (UKBB and EstBB) and CNV-GWAS summary statistics (UKBB) are deposited on Mendeley Data at https://dx.doi.org/10.17632/z54dc3b6jz.1. The accession numbers for UKBB CNV-GWASs summary statistics reported in this paper are GWAS Catalog: GCST90027274–GCST90027444.

## Web resources

DECIPHER CNV Syndromes list, https://www.deciphergenomics.org/disorders/syndromes/list
GeneReviews, Mefford, H. (2021). 17q12 Recurrent Duplication, https://www.ncbi.nlm.nih.gov/books/NBK344340/
GeneReviews, Mitchel, M.W., Moreno-De-Luca, D., Myers, S.M., Levy, R.V., Turner, S., Ledbetter, D.H., and Martin, C.L. (2020). 17q12 Recurrent Deletion Syndrome, https://www.ncbi.nlm.nih.gov/books/NBK401562/
Genome Reference Consortium GRCh37, https://www.ncbi.nlm.nih.gov/grc/human/data
gnomAD (v2.1.1), https://gnomad.broadinstitute.org/
GTEx project (v8) portal, https://gtexportal.org/home/
Neale Lab UKBB genetic correlation, https://ukbb-rg.hail.is/
NHGRI-EBI GWAS Catalog, https://www.ebi.ac.uk/gwas/
OMIM, https://www.omim.org/
ScyMed MediCalc, http://www.scymed.com/en/smnxfd/smnxfdad.htm

## Supplemental information

Data S1. Tables S1–S8

Document S2. Article plus supplemental information
