# Supplementary material for: The individual and global impact of copy-number variants on complex human traits
Source: Am J Hum Genet. 2022 Mar 2;109(4):647–68. doi: 10.1016/j.ajhg.2022.02.010 (PMC9069145; doi:10.1016/j.ajhg.2022.02.010)
Supplement: Document S1. Figures S1–S9 and supplemental material and methods [file mmc1.pdf]

The American Journal of Human Genetics, Volume *109*

Supplemental information

The individual and global impact of copy-number variants on complex human traits

Chiara Auwerx, Maarja Lepamets, Marie C. Sadler, Marion Patxot, Miloš Stojanov, David Baud, Reedik Mägi, Estonian Biobank Research Team, Eleonora Porcu, Alexandre Reymond, and Zoltán Kutalik

SUPPLEMENTAL FIGURES

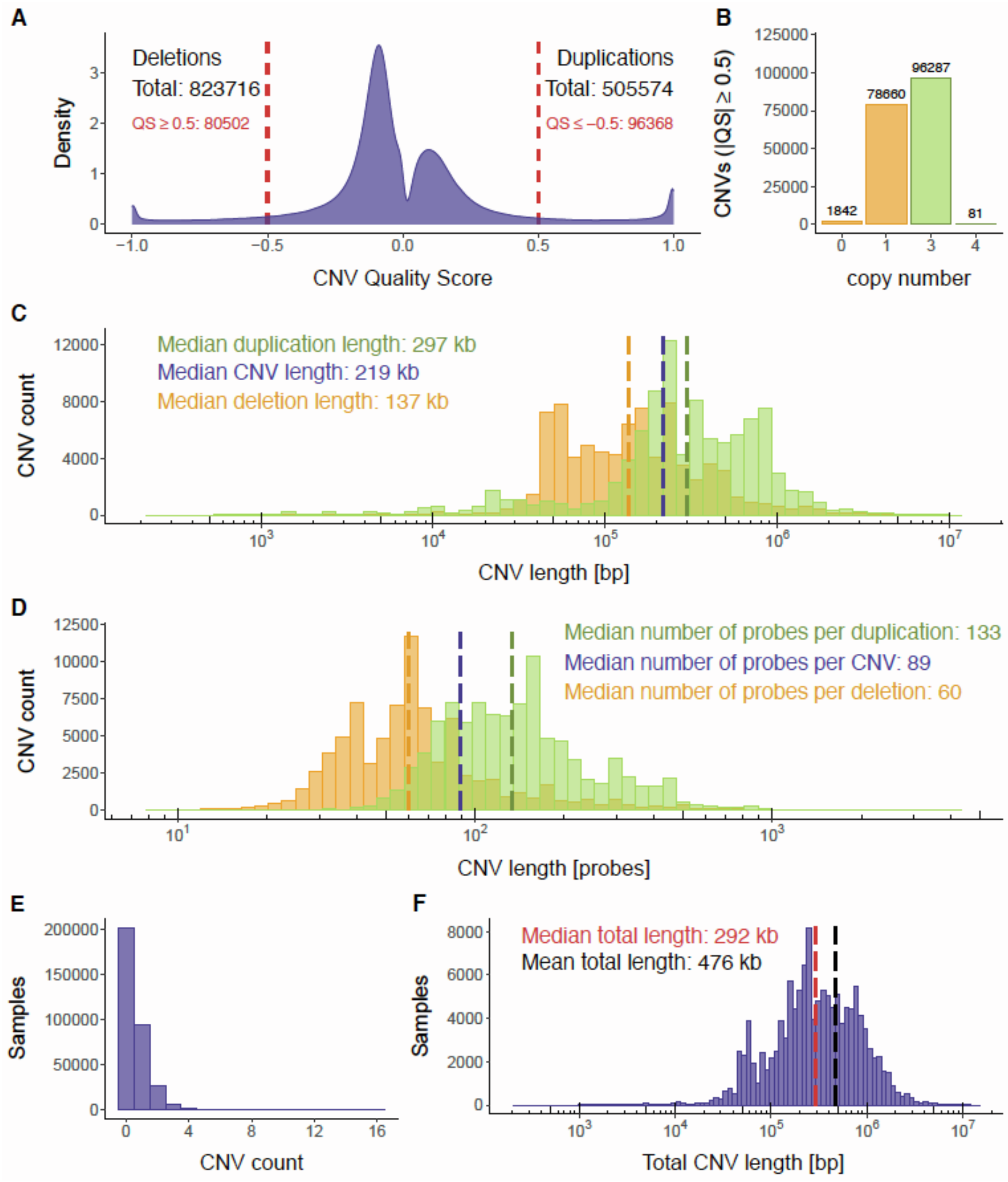

Figure S1. Distribution of high confidence CNVs in the UK Biobank

(A) Density plot of quality scores (QSs) for the 1’329’290 called CNVs. High confidence duplications (QS ≥ 0.5) and deletions (QS ≤ -0.5), indicated per the red dashed lines, were retained for downstream analyses. (B) Distribution of the copy number state of high confidence CNVs. Deletions (copy number 0 or 1) are in orange, duplications (copy number 3 or 4) are in green. Number of CNVs in each category is indicated on top of the bars. Distribution of high confidence duplications (green) and deletions (orange) length in base pairs (C) and number of probes (D) on a logarithmic scale. Dashed lines show the median duplication (green), CNV (purple), and deletion (orange) length. (E) Distribution of high confidence CNV counts per individual. (F) Distribution of the total amount of bases affected by high confidence CNVs per individual on a logarithmic scale; red and black dashed lines show the median and mean number of bases affected by CNVs among individuals with at least 1 CNV, respectively.

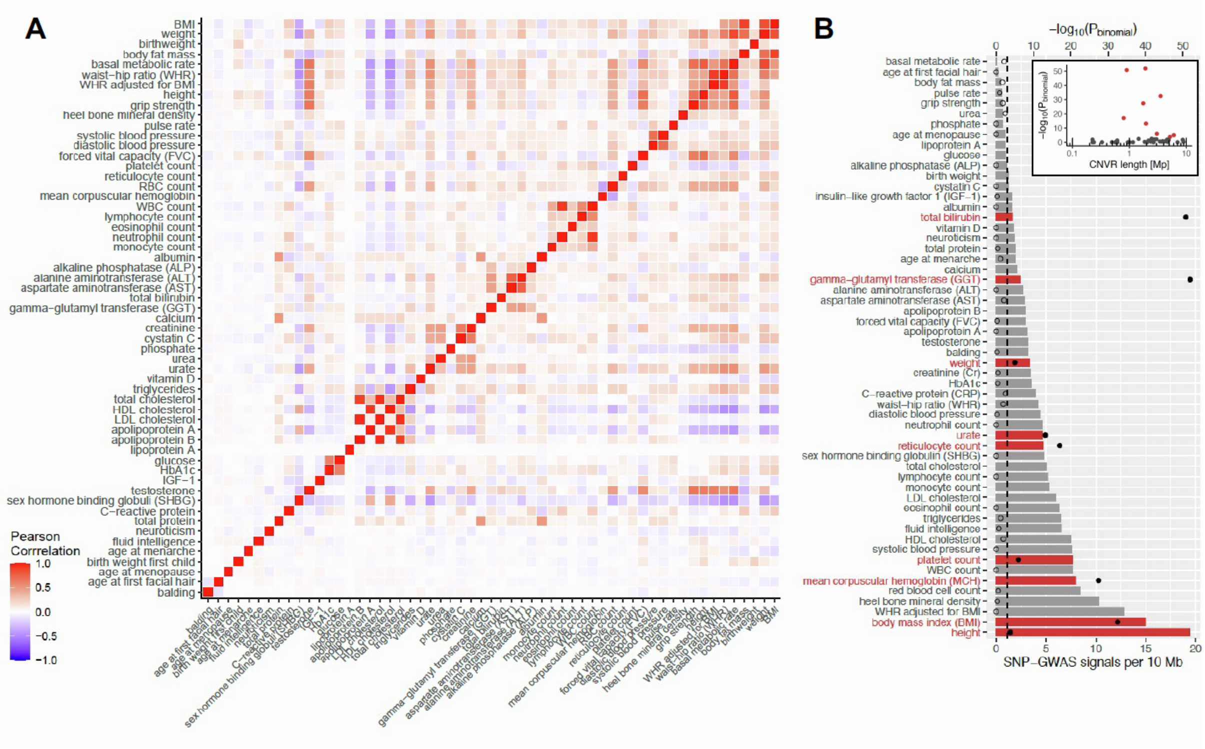

Figure S2. Genome-wide association studies of complex traits

(A) Pearson correlation across the 57 continuous traits assessed by CNV-GWAS. (B) Number of autosomal NHGRI-EBI GWAS Catalog (Web Resources)[1] SNP-GWAS signals per 10 Mb of autosomal genome (bottom x-axis; as horizontal bars) for 56 of the assessed traits and their synonyms (y-axis; Table S1). The top x-axis indicates the negative logarithm of the *p*-value (as a dot) for the binomial test that assesses the SNP-GWAS signal distribution within CNV regions, as compared to the entire autosomal genome. Non-significant traits are in grey with an empty dot; significant traits ($p \leq 0.05/56$; above the black dashed line) are in red with full dots. The inset shows the negative logarithm of the binomial test's *p*-value against the total length of trait-associated CNVRs in Mb on a logarithmic scale for each trait. Significant traits ($p \leq 0.05/56$) are in red, non-significant ones in grey.

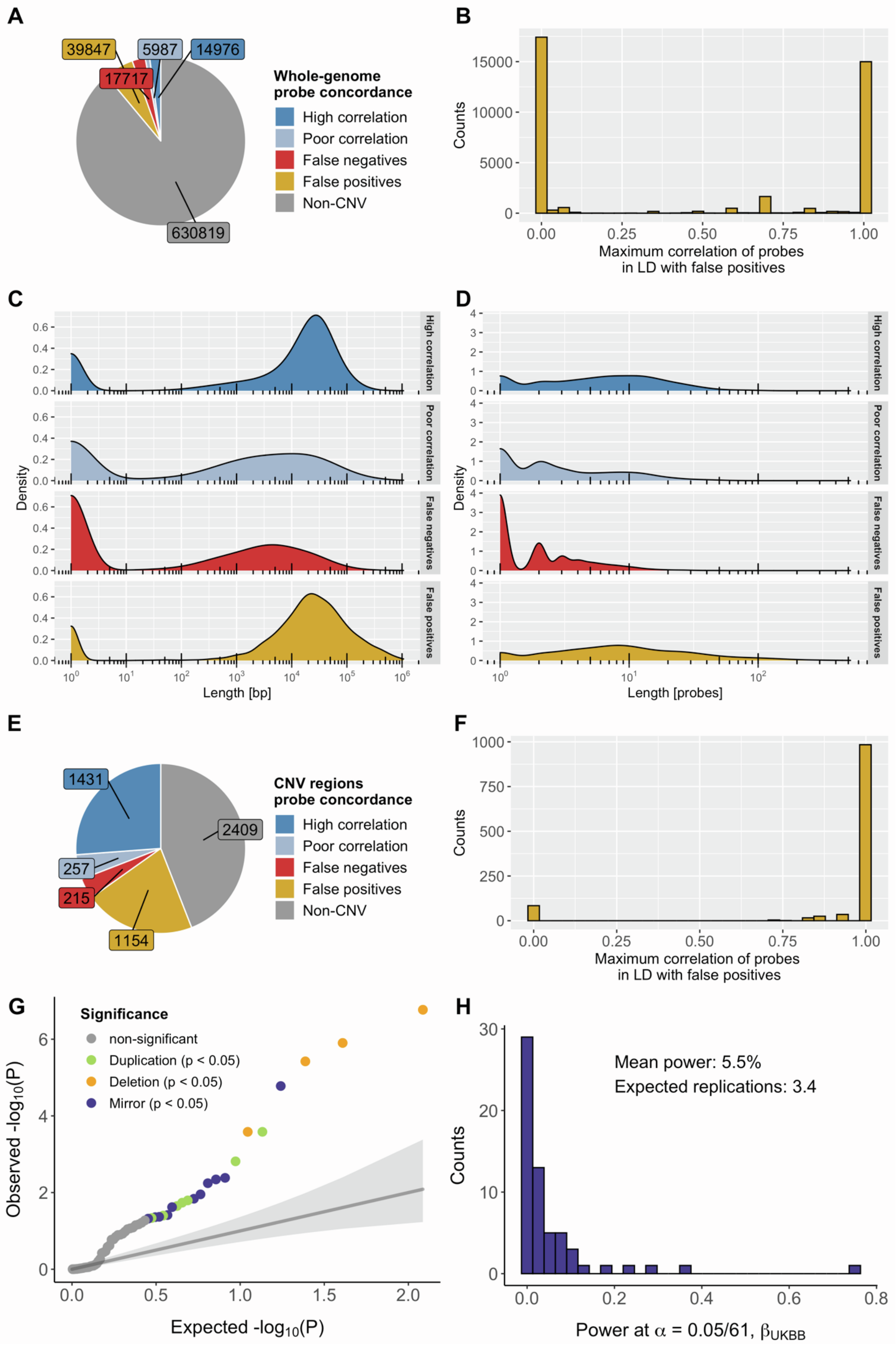

Auwerx et al., 2022

Figure S3. Replication in the Estonian Biobank

(A) Concordance between PennCNV (microarray) and STRiP (whole-genome sequencing) CNV calls for 709’358 quality-controlled, autosomal probes from the OmniExpress-24 genotyping array in the Estonian Biobank (EstBB). Probes with high ($r \geq 0.75$) and low ($r < 0.75$) correlation between PennCNV and STRiP CNV profiles are in dark and light blue, respectively; false negatives (*i.e.* probes only detected in a CNV state by STRiP) are in red; false positives (*i.e.* probes only detected in a CNV state by PennCNV) are in gold; monomorphic probes are in grey. (B) Distribution of the maximal PennCNV-STRiP correlation among probes in linkage disequilibrium (± 250 kb and $r \geq 0.5$) with false positive probes from (A). Size distribution in base pairs (C) and probes (D) of consecutive stretches of probes mapping to non-monomorphic categories in (A). (E) EstBB concordance between PennCNV and STRiP CNV calls at 5’566 probes overlapping UK Biobank trait-associated CNV regions; identical color scheme to (A). (F) Distribution of the maximal PennCNV-STRiP correlation among probes in linkage disequilibrium (± 250 kb and $r \geq 0.5$) with false positive probes from (E). (G) Expected versus observed negative logarithm of *p*-values for the 61 CNV-trait pairs assessed in the EstBB. Directionally concordant and nominally significant ($p \leq 0.05$) signals replicated with the duplication-only, deletion-only, or mirror association model are in green, orange, or purple, respectively. Non-significant signals ($p > 0.05$) are in grey. Shade represents the 95% confidence interval around expected values. (H) Distribution of replication power at $\alpha = 0.05/61 = 8.2x10^{-4}$, assuming similar effect sizes to the one observed in the UKBB, for the 61 signals assessed in (G). The mean power is 5.5%, corresponding to an expected number of multiple testing correction surviving replications of 0.055*61 = 3.4.

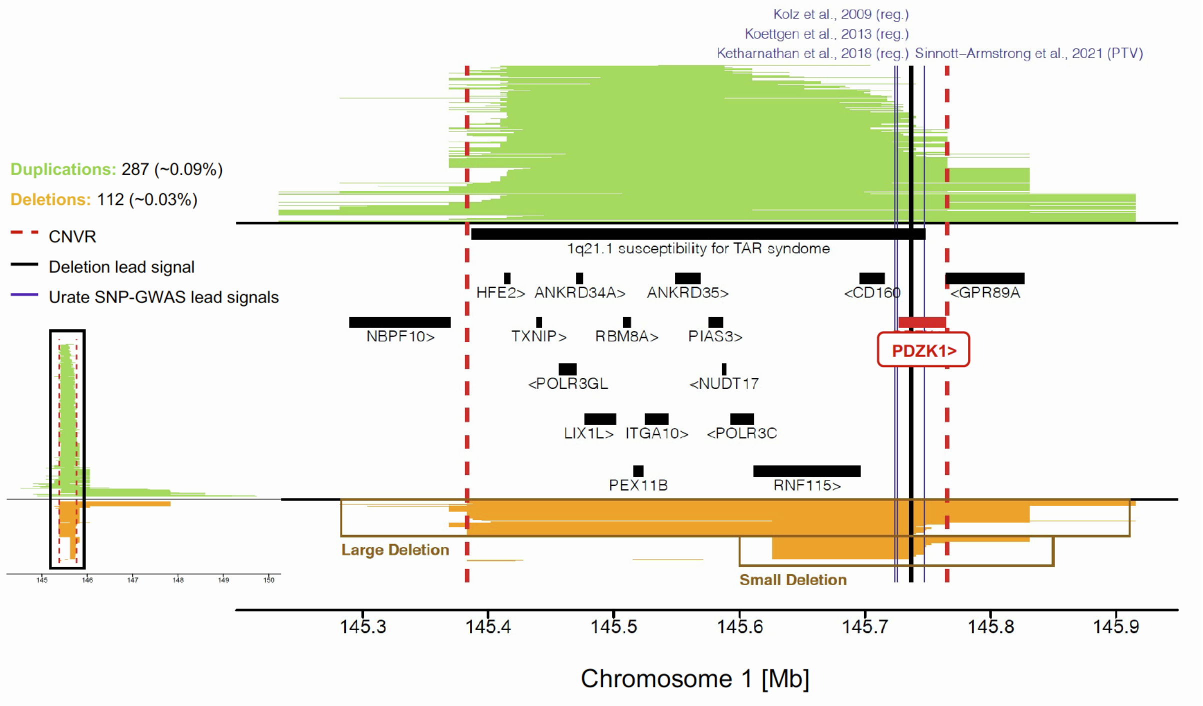

Figure S4. 1q21.1 deletion and decreased serum urate levels

Mapping of CNVs overlapping the 1q21.1 (chr1:145’383’239-145’765’206) deletion region associated with decreased serum urate levels. Number and frequency of duplications and deletions are at the top left; left plot shows all overlapping CNVs; right plot focuses on the central CNV region represented by red dashed lines. Duplications are in green, deletions in orange; black line indicates the lead signal for serum urate (deletion-only); purple lines indicate serum urate-associated SNPs[2–5] (reg. = regulatory variant; PTV = protein-truncating variant). DECIPHER recurrent CNV and overlapping protein-coding genes are in black, except for *PDZK1* in red. Brown boxes separate large (start < 145.6 Mb) versus small (start ≥ 145.6 Mb) deletion carriers.

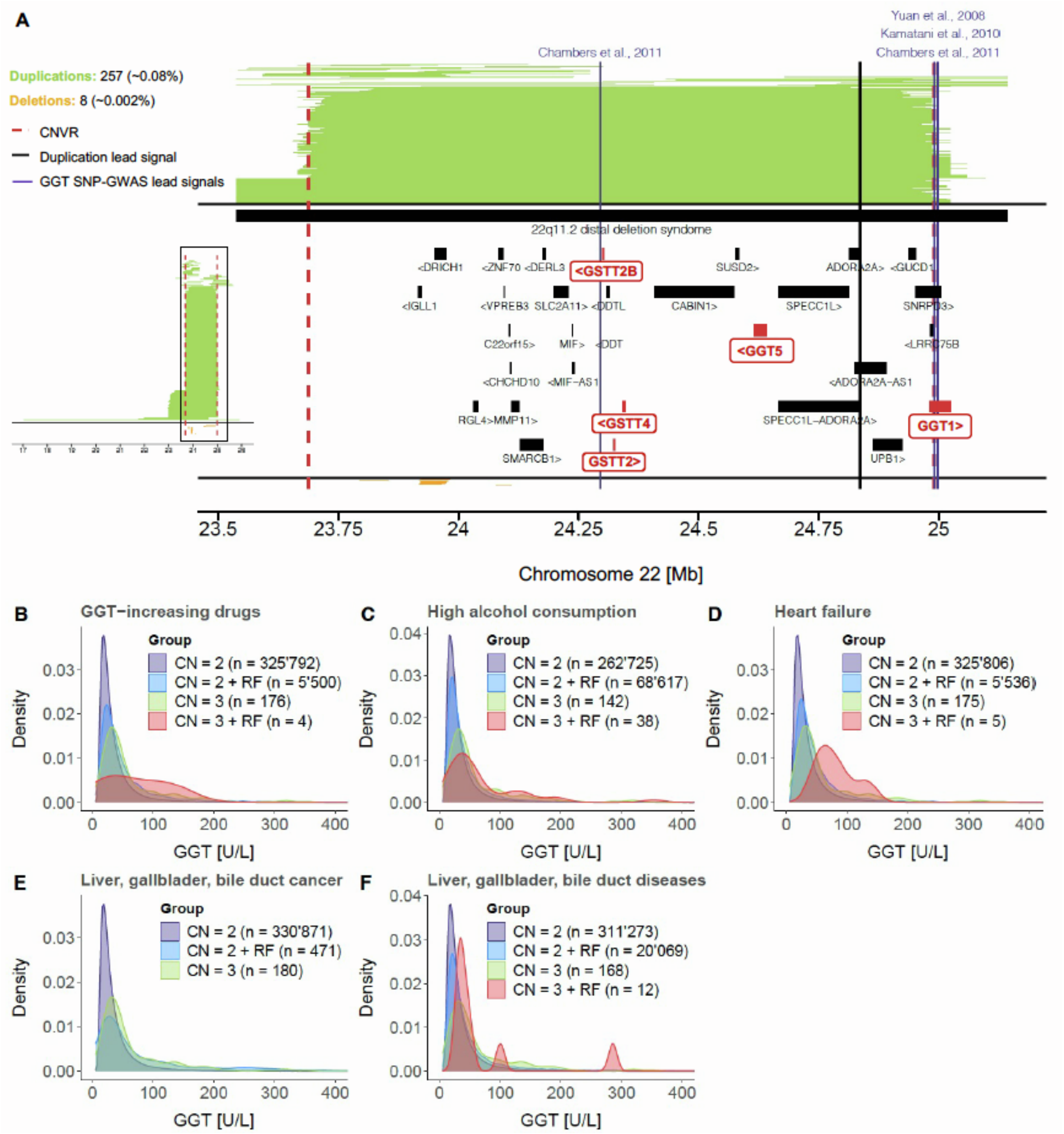

Figure S5. 22q11.23 duplication and increased serum γ-glutamyl transferase levels

(A) Mapping of CNVs overlapping the 22q11.23 (chr22:23'688'345-24'990'213) duplication associated with increased γ-glutamyl transferase (GGT) levels. Number and frequency of duplications and deletions are at the top left; left plot shows all overlapping CNVs; right plot focuses on the central CNV region represented by red dashed lines. Duplications are in green, deletions in orange; black line indicates the lead signal for GGT (duplication-only); purple lines indicate GGT-associated SNPs[6–8]. DECIPHER recurrent CNV (truncated) and overlapping protein-coding genes are in black, except for genes involved in glutathione metabolism in red. (B-F) Density plots showing the distribution of GGT levels in copy-neutral (CN = 2) and 22q11.23 (chr22:23'688'345-24'990'213) overlapping duplication carriers (CN = 3) with or without various risk factors (RF) for increased GGT: (B) GGT-increasing drugs, (C) high alcohol consumption, (D) heart failure, and (E) cancer or (F) other diseases of the liver, gallbladder, or bile ducts. The sample size (n) for each category is indicated. Plots were truncated to 400 U/L (maximum value: 1167 U/L).

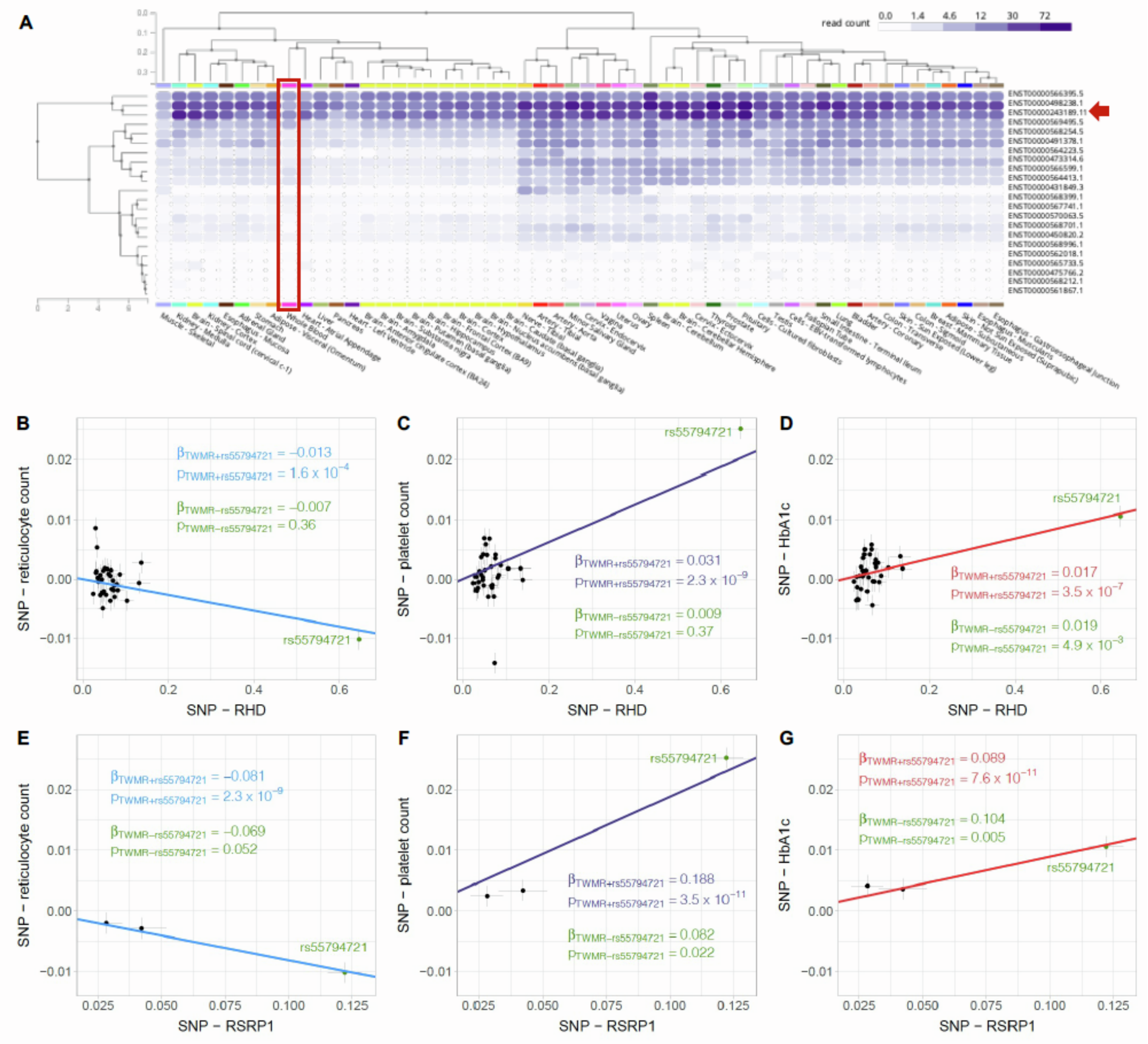

Figure S6. 1p36.11 deletion and altered hematological traits

(A) GTEx v8 (Web Resources)[9] isoform expression for *RSRP1* in 54 tissues. Whole blood is circled in red. The red arrow points at the isoform with the highest expression in whole blood, ENST00000243189. (B-G) SNP-exposure (x-axis) versus SNP-outcome (y-axis) plots for transcriptome-wide Mendelian randomization (TWMR) analyses estimating the causal effect of (B) *RHD* → reticulocyte count, (C) *RHD* → platelet count, (D) *RHD* → glycated hemoglobin (HbA1c), (E) *RSRP1* → reticulocyte count, (F) *RSRP1* → platelet count, and (G) *RSRP1* → HbA1c. Grey horizontal and vertical lines represent the standard errors around the SNP-exposure and SNP-outcome estimates, respectively. Causal effect estimates (β) and associated *p*-values are reported for each analysis. The outlier SNP rs55794721 is in green. Causal effects were re-estimated omitting rs55794721 and are reported in green.

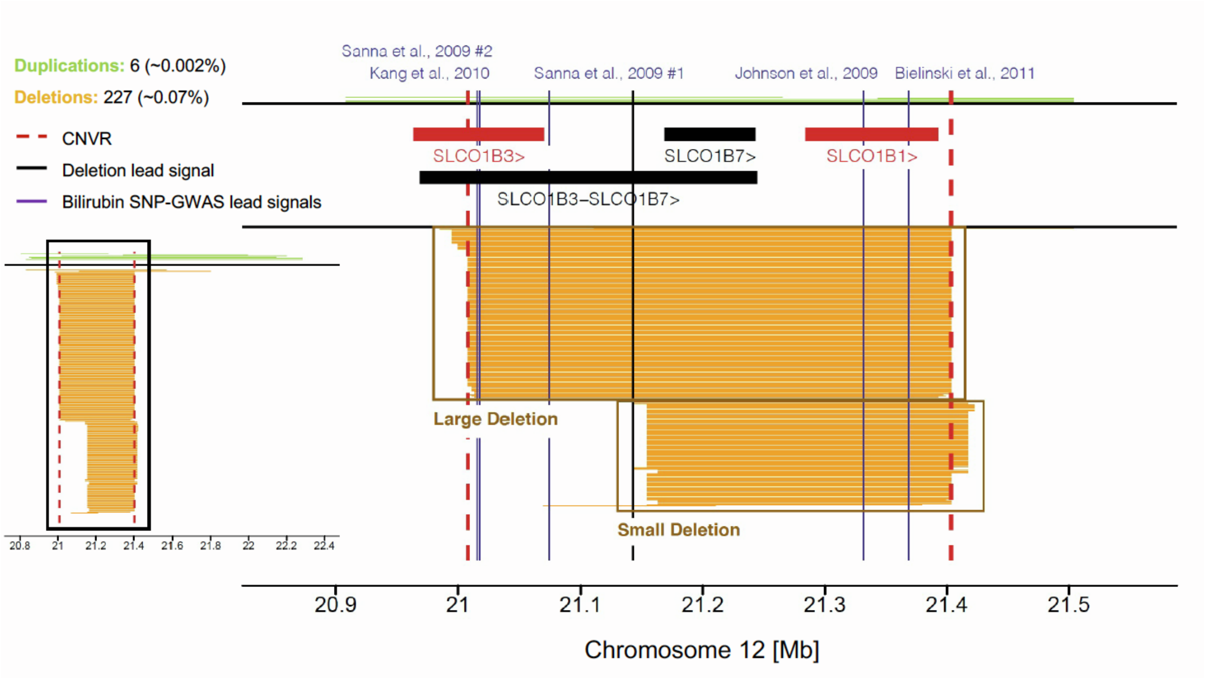

Figure S7. 12p12.2-p12.1 deletion and increased total bilirubin levels

Mapping of CNVs overlapping the 12p12.2-12p12.1 (chr12:21'008'080-21'403'457) deletion associated with increased total bilirubin levels. Number and frequency of duplications and deletions are at the top left; left plot shows all overlapping CNVs; right plot focuses on the central CNV region represented by red dashed lines. Duplications are in green, deletions in orange; black line indicates the lead signal for total bilirubin (deletion-only); purple lines indicate serum bilirubin-associated SNPs[10–13]; overlapping protein-coding genes are shown in black, except for Rotor syndrome-associated genes, *SLCO1B1* and *SLCO1B3*, shown in red. Brown boxes separate large (start < 21.1 Mb) versus small (start ≥ 21.1 Mb) deletion carriers.

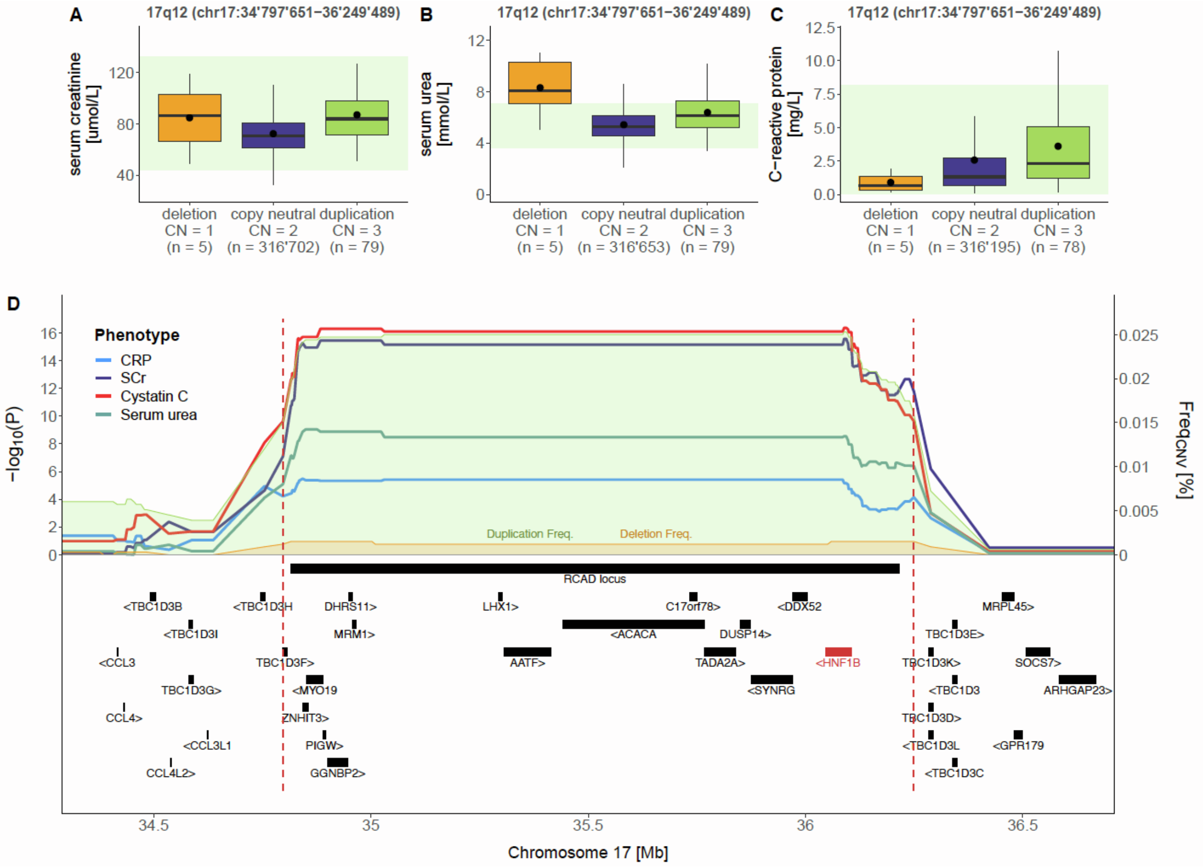

Figure S8. 17q12 CNVs and renal phenotypes

Boxplots representing levels of (A) serum creatinine (SCr), (B) serum urea, and (C) C-reactive protein (CRP) in individuals with a 17q12 (chr17:34’797’651-36’249’489) overlapping deletion, copy-neutrality, or duplication. Copy number (CN) and sample size (n) are reported for each category; dots show the mean; outliers are not shown; green bands show normal clinical range for (A) SCr: 44.2-132.6 µmol/L, (B) serum urea: 3.6-7.1 mg/L, and (C) CRP: 0.07-8.2 mg/L. (D) Association plot for the 17q12 (chr17:34’797’651-36’249’489) CNV region. Red dashed lines delimit the duplication-only associated CNV region; left y-axis shows the negative logarithm of association *p*-value for CRP (blue), SCr (purple), cystatin C (red), and serum urea (turquoise); right y-axis shows CNV frequency [%], with duplication frequency in green and deletion frequency in orange; overlapping DECIPHER recurrent CNV and genes are in black, except for the putative causal gene, *HNF1B*, in red.

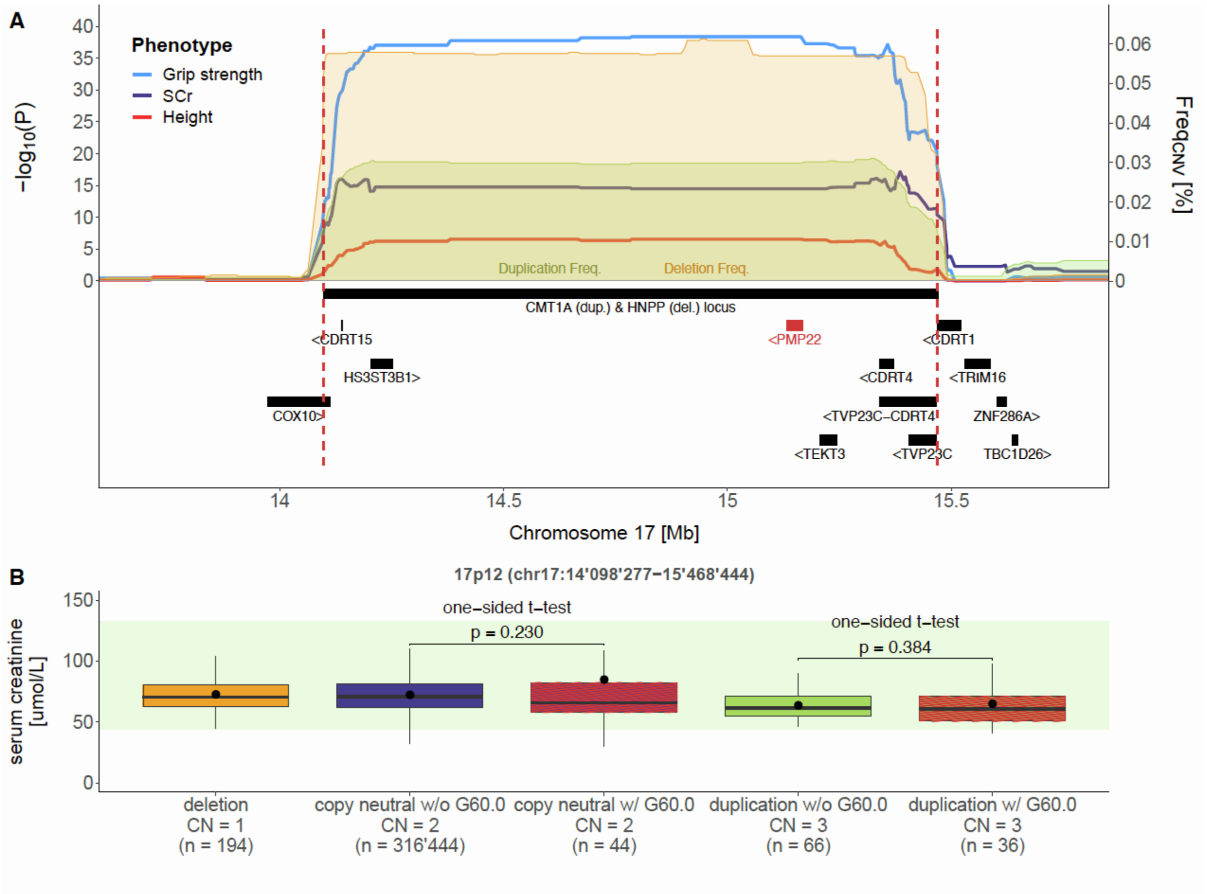

Figure S9. 17p12 duplication and muscle phenotypes

(A) Association plot for the 17p12 (chr17:14'098'277-15'468'444) overlapping CNV region. Red dashed lines delimit the duplication-only associated CNV region; left y-axis represents the negative logarithm of association *p*-values for hand grip strength (blue), serum creatinine (SCr; purple), and height C (red); right y-axis represents CNV frequency [%], with duplication frequency in green and deletion frequency in orange; overlapping DECIPHER recurrent CNV and genes are in black, except for the putative causal gene, *PMP22*, in red. (B) Boxplots representing SCr levels of individuals with a 17p12 (chr17:14'098'277-15'468'444) overlapping deletion, copy-neutrality, or duplication, the two latter being split according to the presence (w/) or absence (w/o) of a hereditary motor or sensory neuropathy diagnosis (ICD-10 G60.0; red stripes). Copy number (CN) and sample size (n) are reported for each category; dots show the mean; outliers are not shown; green bands show normal clinical range for SCr: 44.2-132.6 µmol/L.

SUPPLEMENTAL MATERIAL AND METHODS

Note 1: Cohort descriptions

The UK Biobank cohort: The UK Biobank (UKBB) is a volunteer-based cohort of ~500’000 individuals (54% females) from the general UK population[14]. Individuals were aged 40-69 years at recruitment and underwent microarray-based genotyping and extensive phenotyping, which is constantly extended and includes physical measurements, blood biomarker analyses, socio-demographic and health-related questionaries, as well as linkage to medical health records. Participants signed a broad informed consent form and data was accessed through the application number 16389.

The Estonian Biobank cohort: The Estonian Biobank (EstBB) is a population-based cohort encompassing ~20% of Estonia’s adult population (~200’000 individuals; 66% females; Web Resources)[15]. Individuals underwent microarray-based genotyping at the Core Genotyping Lab of the Institute of Genomics, University of Tartu, and a subset of ~2’500 samples underwent whole-genome sequencing (WGS). General data, including body measurements, were collected at recruitment. Project-based questionnaires were sent later and filled on a voluntary basis. Health records are updated through linkage with the national Health Insurance Fond and other relevant databases, providing sporadic access to blood biomarker measurements and medical diagnoses. All participants signed a broad informed consent form and analyses were carried out under ethical approval 1.1-12/624 from the Estonian Committee on Bioethics and Human Research and data release N05 from the EstBB.

The CHUV maternity cohort: The Lausanne University Hospital (CHUV) maternity cohort was designed as a serological surveillance study of maternal toxoplasmosis infections and approval from the Ethics Committee of Vaud (CER-VD) was obtained for data reusage under the project ID 2019-00280 to investigate maternal and fetal outcomes. Rhesus (Rh) blood

groups were serologically determined for 5'164 women. Reticulocyte count, platelet count, glycated hemoglobin (HbA1c) levels, intrapartum reports, and International Classification of Diseases, 10th Revision (ICD-10) codes were sporadically collected between 2009-2014.

Note 2: CNV calling and quality score pipeline

CNV calling: Autosomal and pseudoautosomal copy number variant (CNV) calling was performed in parallel for the 106 genotyping batches using PennCNV[16]. Individual intensity files were generated from the B allele frequency (BAF) and Log R Ratio (LRR) files available on the UKBB portal. Missing values (-1) were set to NA. Batch-specific population frequency of the B allele (PFB) files were generated. Probes with missing PFB were removed in a batch-specific way. The hidden Markov Model file for Affymetrix genome-wide 6.0 array was downloaded as part of the PennCNV-Affy package and used without training. The GC model file was generated following instructions of `cal_gc_snp.pl`, using gc5Base downloaded from the UCSC Genome Browser (03/2020; Web Resources). Above-described files were used to call CNVs with confidence score using `detect_cnv.pl` with genomic wave adjustment. Adjacent CNVs (gap ≤ 20% of merged CNV length) were merged with `clean_cnv.pl`. Chromosome X CNVs were called separately with the PennCNV inbuilt arguments `-chrx` and `-sexfile` when running `detect_cnv.pl`. Copy-neutral losses of heterozygosity resulting from male chromosome X hemizygosity were excluded, and adjacent CNVs were merged.

Quality score pipeline: Hurdles linked to CNV analysis include high false positive rates and variability in break points. To mitigate these issues, a post-PennCNV processing pipeline was used to attribute a quality score (QS) to each CNV and transform calls to the probe level[17,18]. QSs range from -1 to 1 and reflect the probability of a CNV to be a true positive (-1 = likely deletion; 1 = likely duplication; ~0 = low confidence CNVs). Briefly, PennCNV `filter_cnv.pl` was run separately on autosomal/pseudoautosomal and chromosome X

CNVs (with `-chrx`), resulting in two sample-level quality control (QC) files. These were combined by adding the number of chromosome X CNVs to the autosomal/pseudoautosomal sample-level QC file. A single file containing all called CNVs, as well as associated CNV- and sample-level QC metadata, was generated and used to attribute a QS to each called CNV. Next, linear PennCNV coordinates were transformed into per-chromosome $probe\ x\ sample$ matrices, with entries reflecting the QS attributed to the CNV mapping to these probes. Copy neutral probes are indicated by 0 and individuals with no CNVs were added as all-0 columns.

Note 3: CNV association studies in the UKBB

Number of effective tests: As retained CNV-proxy probes remain highly correlated – much more so than SNPs would be due to classical linkage disequilibrium patterns – the number of effective tests, N_{eff}, was determined[18,19]. Per-chromosome $probe\ x\ sample$ genotype matrices G were generated, with genotypes taking values of -1 (deletion), 0 (copy-neutral), and 1 (duplication). Chromosome-wise N_{eff} were defined as the number of eigenvalues required to explain 99.5% of the variance in G and were summed up, resulting in a genome-wide (GW) N_{eff}. N_{eff} was estimated at 11'804, setting the GW threshold for significance at $p \leq 0.05/11'804 = 4.2 \times 10^{-6}$.

Phenotype selection: Fifty-seven continuous traits were selected based on data availability and presumed high heritability. Fifty-four were defined as the mean of measured instances. Three were composite traits: Grip strength, as the mean of *hand grip strength left* and *right* (#46, #47); waist-to-hip ratio (WHR), as the ratio between *waist* and *hip circumference* (#48, #49); WHR adjusted for body mass index (WHRadjBMI), by correcting WHR for BMI. Two were male-specific (*relative age of first facial hair* (#2375); *hair/balding pattern* (#2395)) and three were female-specific (*age when periods started (menarche)* (#2714); *age at menopause (last menstrual period)* (#3581); *birth weight of first child* (#2744)). Entries “do not know”, “only had twins”, “prefer not to answer” were set as missing.

Stepwise conditional analysis: Stepwise conditional analysis was performed on CNV-GWAS results to determine the number of independent signals per trait. For traits with ≥ 1 GW-significant signal, CNV information was extracted for the lead probe, with genotypes taking values of -1 (deletion), 0 (neutral), and 1 (duplication) for the mirror model, and setting deletions or duplications to NA when considering the duplication-only or deletion-only models, respectively. Lead CNV probe effect was regressed out of the phenotype and association studies were conducted anew. These steps were repeated until no GW-associated probes were identified.

Note 4: Replication in the Estonian Biobank

Comparative analysis of CNV quality

CNV detection in OmniExpress-24 genotype data: About 7'750 EstBB participants were genotyped with Illumina Infinium OmniExpress-24 genotyping array. Samples with genotype call rate < 98%, Hardy-Weinberg equilibrium test *p*-value < $1x10^{-4}$, or mismatched sex based on chromosome X heterozygosity were excluded. Intensity files (LRR and BAF) were created with Illumina GenomeStudio v2.0.4. A PFB file was generated from all samples. Only autosomal probes were carried over to the CNV detection step (709'358 probes), which was performed with PennCNV[16] analogously to what has been described for the UKBB. Samples with > 200 CNVs or a total length of CNV calls > 10 Mb were excluded. CNVs were attributed a QS, as previously described for the UKBB, and CNVs with |QS| ≥ 0.5 were retained. To harmonize data with WGS-based CNV calls, duplications < 2 kb and deletions < 1 kb were excluded.

CNV detection in WGS data: WGS data (30x) was available for ~2'500 EstBB samples. WGS-based autosomal CNVs were called in 5 batches using the Genome STRiP pipeline[20]. Eleven samples with $calls/sample > median + 3 * median\ absolute\ deviation$ were

removed. The union of the discovered sites was genotyped with Genome STRiP SVGenotyper module in all batches separately and merged. Duplicate calls were removed using standard Genome STRiP duplicate removal settings (overlap > 50% and duplicate score > 0). Low-quality CNVs and CNVs with call rate < 90% were excluded. Duplications < 2 kb and deletions < 1 kb were excluded. To harmonize data with microarray-based CNV calls, CNVs > 10 Mb were excluded and adjacent CNVs were merged (gap ≤ 20% of merged CNV length).

CNV calling and copy number association studies

Global Screening Array (GSA) genotype data: Twelve batches containing 202’282 EstBB participants were genotyped with Illumina GSAv1.0, GSAv2.0, GSAv2.0_ESTChip, and GSAv3.0_ESTChip2. Samples with genotype call rate < 98%, Hardy-Weinberg equilibrium test *p*-value < $1x10^{-4}$, or mismatched sex based on chromosome X heterozygosity were excluded and one of each duplicated sample was retained. Genotypes were re-clustered by manual realignment of cluster location. Intensity files (LRR and BAF) were created with Illumina GenomeStudio v2.0.4. A PFB file was generated from 1’000 randomly selected samples from batch 1. Only autosomal probes overlapping all GSA versions (excluding custom ESTChip probes) were carried over to the CNV detection step (671’035 probes; 242’091 probes overlap with UKBB).

Sample quality control: Samples originating from 2 batches with outlier genotyping intensity parameters, as well as genotyping plates with > 3 samples with either > 200 called CNV or a total length of CNV calls > 10 Mb were excluded. Individual samples meeting these criteria were removed. Among related pairs (KING kinship coefficient > 0.0884), the sample with most available phenotypes was retained.

Power analysis: Simulations were conducted to estimate the power of our replication study. We defined $\beta_{i,j}$ as the standardized effect of probe i on trait j observed in the UKBB, $q_{i,dup}$ and

$q_{i,del}$ the duplication and deletion frequencies of probe i in the EstBB, respectively, and N_j the sample size for trait j in the EstBB. When considering mirror signals, CNVs were simulated for N_j samples as:

$$CNV = \begin{cases} -1, & z \leq q_{i,del} \\ 0, & q_{i,del} < z < 1 - q_{i,dup} \\ 1, & z \geq 1 - q_{i,dup} \end{cases}$$

With $z \sim U(0,1)$. For duplication-only or deletion-only signals, duplications or deletions were simulated for N_j samples as $CNV \sim Bernoulli(q_{i,dup})$ or $CNV \sim Bernoulli(q_{i,del})$, respectively. Normally distributed error terms ε were simulated according to $\varepsilon \sim N(0, \sigma^2)$ for N_j samples. For mirror signals, the noise variance σ^2 was defined as $\sigma^2 = \sigma_j^2 - var(CNV) * \beta_{i,j}^2$, with σ_j^2 the observed standardized variance for trait j in the EstBB equaling 1. For duplication-only and deletion-only signals, σ^2 was defined as $\sigma^2 = \sigma_j^2 - q_{i,dup} * (1 - q_{i,dup}) * \beta_{i,j}^2$ and $\sigma^2 = \sigma_j^2 - q_{i,del} * (1 - q_{i,del}) * \beta_{i,j}^2$, respectively. Phenotypes Y were simulated for N_j samples as $Y = CNV * \beta_{i,j} + \varepsilon$. When simulated data contained ≥ 1 CNV carrier, the *p*-value for the estimated effect size from the linear regression $Y \sim CNV$ was computed and retained. Otherwise, the *p*-value was set as missing. For each signal, 10'000 simulations were conducted, and power was defined as the fraction of non-missing *p*-values ≤ 8.2×10^{-4}.

Note 5: Extended phenotypic assessment

Disease diagnoses: To assess patients' disease status, ICD-10 diagnoses were used (#41270):

- γ-glutamyl transferase (GGT)-altering diseases: *heart failure* (I50), *malignant neoplasm of liver and intrahepatic bile ducts* (C22), gallbladder (C23), and *other unspecified parts of biliary tract* (C24), as well as other *diseases of the liver* (K70-K77) and the *gallbladder, biliary tract, or pancreas* (K80-K87) were considered.

- Rotor syndrome: Rotor syndrome [MIM: 237450] is classified with Dubin-Johnson syndrome [MIM: 237500] under *other disorders of bilirubin metabolism* (E80.6).
- Charcot-Marie-Tooth type 1A: CMT1A [MIM:118220] is classified as *hereditary motor or sensory neuropathy* (G60.0), a diagnosis encompassing all forms of CMT and related neuropathies.

Lifestyle:

- Self-reported high alcohol consumption (#1558): *daily or almost daily*.
- GGT-increasing drug usage[21] (#20003): 2038459704 (carbamazepine), 1140865426 (cimetidine), 1140909708 (furosemide), 1140869848 (methotrexate), 1140910706 (phenobarbital), 2038460076 (phenytoin).

Socio-economic factors:

- *Townsend deprivation index at recruitment* (#189).
- *Average total household income before tax* (#738): averaged over measured instances.
- *Age completed full time education* (#845): proxy for educational attainment (EA); averaged over measured instances.

Life history traits:

- *Age at recruitment* (#21022)
- *Mother's* (#3526) and *father's* (#1807) *age at death:* averaged over measured instances; meta-analyzed as parental lifespan.
- *Relative leucocyte telomere length adjusted for the influence of technical parameters* (#22191): averaged over measured instances.

For socio-economic factors and life history traits, entries matching "do not know" or "prefer not to answer" were set as missing. Traits were inverse normal transformed prior correction for sex, age (#21003), age^2, genotyping batch, and principal components (PCs) 1-40, except for *age at recruitment*, which was not corrected for age and age^2.

Note 6: *RHD* and hematological traits

Transcriptome-wide Mendelian Randomization: Using univariable transcriptome-wide Mendelian randomization[22] (TWMR), the causal effect of differential *RHD* and *RSRP1* expression on reticulocyte count, platelet count, and HbA1c was estimated based on independent ($r^2 < 0.01$) genetic variants. Expression quantitative trait loci (eQTLs) were obtained from the eQTLGen consortium and included *cis*-eQTLs (FDR < 0.05, 2-cohort filter) for ~16,900 transcripts[23]. GWAS effect sizes originate from the Neale Lab UKBB summary statistics (Web Resources). Exposure and outcome datasets were harmonized, standardized effect size estimates were obtained by dividing z-scores by the square root of the sample size, and palindromic variants and variants with allele frequency difference > 5% between the two datasets were removed.

Association between Rhesus blood group and hematological traits: Impact of Rh$^-$ blood group on platelet count, reticulocyte count, and HbA1c was assessed in the CHUV maternity cohort through multivariate linear regression that incorporates the covariates: age at measurement, gestational week at measurement, whether the woman was pregnant at measurement (57.5% for reticulocyte count, 35.6% for platelet count, 23.4% for HbA1c), and whether the women had a child prior to the measurement (78.9% for reticulocyte count, 72.7% for platelet count, 96.7% for HbA1c). For women with multiple measurements, one was randomly selected, giving preference to measurements taken outside of pregnancy and excluding measurements taken during a pregnancy that resulted in stillbirth or multiple births. For measurements taken during pregnancy, gestational week at measurement was calculated

from date and gestational age at delivery. When gestational age at delivery was missing (52.9%), mean gestational age at delivery of the cohort (39.13 weeks) was used. For measurements outside of pregnancy, gestational week at measurement was coded as 0. When age at measurement was missing (12.1% for reticulocyte count, 19.1% for platelet count, 22.2% for HbA1c), data was imputed with multivariate imputation by chain equations including covariates. Ten complete imputed sets were analyzed and estimates were combined with `pool()` (R package 'mice' v3.13.0)[24]. One-sided *p*-value were calculated as $p_{new} = \frac{p_{old}}{2}$ in case of directional agreement with the effect observed in the UKBB.

SUPPLEMENTAL WEB RESOURCES

- Estonian Biobank, https://genomics.ut.ee/en/research/estonian-biobank
- Neale Lab UKBB summary statistics, http://www.nealelab.is/uk-biobank/
- NHGRI-EBI GWAS Catalog, https://www.ebi.ac.uk/gwas/
- UCSC genome annotation (hg19) database, https://hgdownload.cse.ucsc.edu/goldenPath/hg19/database/

SUPPLEMENTAL REFERENCES

1. Buniello, A., Macarthur, J.A.L., Cerezo, M., Harris, L.W., Hayhurst, J., Malangone, C., McMahon, A., Morales, J., Mountjoy, E., Sollis, E., et al. (2019). The NHGRI-EBI GWAS Catalog of published genome-wide association studies, targeted arrays and summary statistics 2019. Nucleic Acids Res. *47*, D1005–D1012.
2. Kolz, M., Johnson, T., Sanna, S., Teumer, A., Vitart, V., Perola, M., Mangino, M., Albrecht, E., Wallace, C., Farrall, M., et al. (2009). Meta-analysis of 28,141 individuals identifies common variants within five new loci that influence uric acid concentrations. PLoS Genet. *5*, e1000504.
3. Köttgen, A., Albrecht, E., Teumer, A., Vitart, V., Krumsiek, J., Hundertmark, C., Pistis, G., Ruggiero, D., O'Seaghdha, C.M., Haller, T., et al. (2013). Genome-wide association analyses identify 18 new loci associated with serum urate concentrations. Nat. Genet. *45*, 145–154.
4. Ketharnathan, S., Leask, M., Boocock, J., Phipps-Green, A.J., Antony, J., O'Sullivan, J.M., Merriman, T.R., and Horsfield, J.A. (2018). A non-coding genetic variant maximally associated with serum urate levels is functionally linked to HNF4A-dependent PDZK1 expression. Hum. Mol. Genet. *27*, 3964–3973.
5. Sinnott-Armstrong, N., Tanigawa, Y., Amar, D., Mars, N., Benner, C., Aguirre, M., Venkataraman, G.R., Wainberg, M., Ollila, H.M., Kiiskinen, T., et al. (2021). Genetics of 35 blood and urine biomarkers in the UK Biobank. Nat. Genet. *53*, 185–194.
6. Yuan, X., Waterworth, D., Perry, J.R.B., Lim, N., Song, K., Chambers, J.C., Zhang, W., Vollenweider, P., Stirnadel, H., Johnson, T., et al. (2008). Population-Based Genome-wide Association Studies Reveal Six Loci Influencing Plasma Levels of Liver Enzymes. Am. J. Hum. Genet. *83*, 520–528.
7. Chambers, J.C., Zhang, W., Sehmi, J.S., Li, X., Wass, M.N., Van der Harst, P., Holm, H., Sanna, S., Kavousi, M., Baumeister, S.E., et al. (2011). Genome-wide association study identifies loci influencing concentrations of liver enzymes in plasma. Nat. Genet. *43*, 1131–1138.
8. Kamatani, Y., Matsuda, K., Okada, Y., Kubo, M., Hosono, N., Daigo, Y., Nakamura, Y., and Kamatani, N. (2010). Genome-wide association study of hematological and biochemical traits in a Japanese population. Nat. Genet. *42*, 210–215.
9. Aguet, F., Barbeira, A., Bonazzola, R., Brown, A., Castel, S., Jo, B., Kasela, S., Kim-Hellmuth, S., Liang, Y., Oliva, M., et al. (2020). The GTEx Consortium atlas of genetic regulatory effects across human tissues. Science (80-.). *369*, 1318–1330.
10. Johnson, A.D., Kavousi, M., Smith, A. V., Chen, M.H., Dehghan, A., Aspelund, T., Lin, J.P., van Duijn, C.M., Harris, T.B., Cupples, L.A., et al. (2009). Genome-wide association meta-analysis for total serum bilirubin levels. Hum. Mol. Genet. *18*, 2700–2710.
11. Sanna, S., Busonero, F., Maschio, A., McArdle, P.F., Usala, G., Dei, M., Lai, S., Mulas, A., Piras, M.G., Perseu, L., et al.

(2009). Common variants in the SLCO1B3 locus are associated with bilirubin levels and unconjugated hyperbilirubinemia. Hum. Mol. Genet. *18*, 2711–2718.
12. Kang, T.W., Kim, H.J., Ju, H., Kim, J.H., Jeon, Y.J., Lee, H.C., Kim, K.K., Kim, J.W., Lee, S., Kim, J.Y., et al. (2010). Genome-wide association of serum bilirubin levels in Korean population. Hum. Mol. Genet. *19*, 3672–3678.
13. Bielinski, S.J., Chai, H.S., Pathak, J., Talwalkar, J.A., Limburg, P.J., Gullerud, R.E., Sicotte, H., Klee, E.W., Ross, J.L., Kocher, J.P.A., et al. (2011). Mayo genome consortia: A genotype-phenotype resource for genome-wide association studies with an application to the analysis of circulating bilirubin levels. Mayo Clin. Proc. *86*, 606–614.
14. Bycroft, C., Freeman, C., Petkova, D., Band, G., Elliott, L.T., Sharp, K., Motyer, A., Vukcevic, D., Delaneau, O., O'Connell, J., et al. (2018). The UK Biobank resource with deep phenotyping and genomic data. Nature *562*, 203–209.
15. Leitsalu, L., Haller, T., Esko, T., Tammesoo, M.L., Alavere, H., Snieder, H., Perola, M., Ng, P.C., Mägi, R., Milani, L., et al. (2015). Cohort profile: Estonian biobank of the Estonian genome center, university of Tartu. Int. J. Epidemiol. *44*, 1137–1147.
16. Wang, K., Li, M., Hadley, D., Liu, R., Glessner, J., Grant, S.F.A., Hakonarson, H., and Bucan, M. (2007). PennCNV: An integrated hidden Markov model designed for high-resolution copy number variation detection in whole-genome SNP genotyping data. Genome Res. *17*, 1665–1674.
17. Macé, A., Tuke, M.A., Beckmann, J.S., Lin, L., Jacquemont, S., Weedon, M.N., Reymond, A., and Kutalik, Z. (2016). New quality measure for SNP array based CNV detection. Bioinformatics *32*, 3298–3305.
18. Macé, A., Tuke, M.A., Deelen, P., Kristiansson, K., Mattsson, H., Nõukas, M., Sapkota, Y., Schick, U., Porcu, E., Rüeger, S., et al. (2017). CNV-association meta-analysis in 191,161 European adults reveals new loci associated with anthropometric traits. Nat. Commun. *8*, 1–11.
19. Gao, X., Starmer, J., and Martin, E.R. (2008). A multiple testing correction method for genetic association studies using correlated single nucleotide polymorphisms. Genet. Epidemiol. *32*, 361–369.
20. Handsaker, R.E., Van Doren, V., Berman, J.R., Genovese, G., Kashin, S., Boettger, L.M., and Mccarroll, S.A. (2015). Large multiallelic copy number variations in humans. Nat. Genet. *47*, 296–303.
21. Dufour, D.R., Lott, J.A., Nolte, F.S., Gretch, D.R., Koff, R.S., and Seeff, L.B. (2000). Diagnosis and monitoring of hepatic injury. II. Recommendations for use of laboratory tests in screening, diagnosis, and monitoring. Clin. Chem. *46*, 2050–2068.
22. Porcu, E., Rüeger, S., Lepik, K., Agbessi, M., Ahsan, H., Alves, I., Andiappan, A., Arindrarto, W., Awadalla, P., Battle, A., et al. (2019). Mendelian randomization integrating GWAS and eQTL data reveals genetic determinants of complex and clinical traits. Nat. Commun. *10*, 1–12.
23. Võsa, U., Claringbould, A., Westra, H.J., Bonder, M.J., Deelen, P., Zeng, B., Kirsten, H., Saha, A., Kreuzhuber, R., Yazar, S., et al. (2021). Large-scale cis- and trans-eQTL analyses identify thousands of genetic loci and polygenic scores that regulate blood gene expression. Nat. Genet. *53*, 1300–1310.
24. van Buuren, S., and Groothuis-Oudshoorn, K. (2011). mice: Multivariate imputation by chained equations in R. J. Stat. Softw. *45*, 1–67.
