# Supplementary material for: The individual and global impact of copy-number variants on complex human traits
Source: Am J Hum Genet. 2022 Mar 2;109(4):647–68. doi: 10.1016/j.ajhg.2022.02.010 (PMC9069145; doi:10.1016/j.ajhg.2022.02.010)
Supplement: Document S2. Article plus supplemental information [file mmc3.pdf]

ARTICLE

The individual and global impact of copy-number variants on complex human traits

Graphical abstract

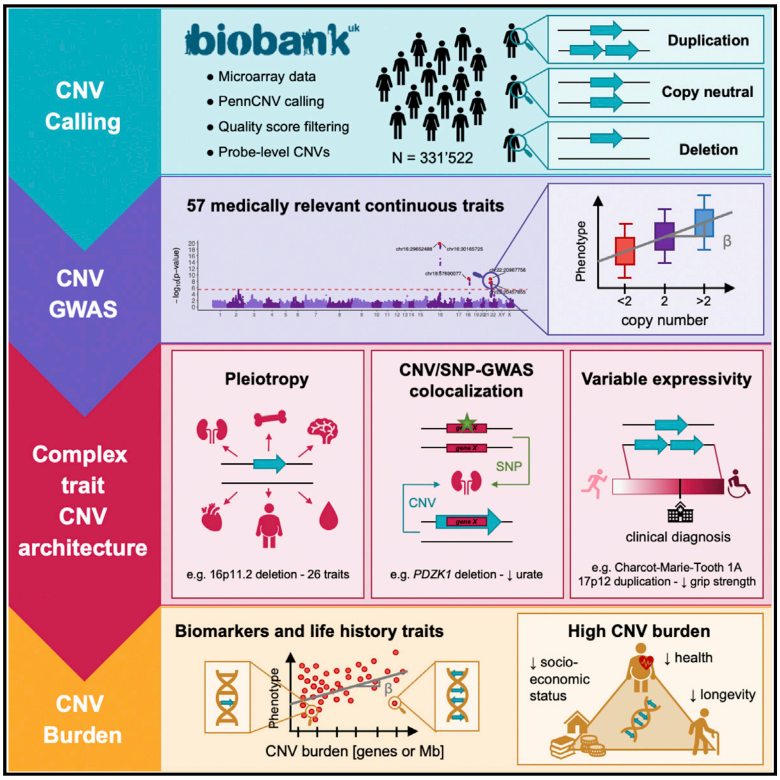

Authors

Chiara Auwerx, Maarja Lepamets, Marie C. Sadler, ..., Eleonora Porcu, Alexandre Reymond, Zoltán Kutalik

Correspondence

alexandre.reymond@unil.ch (A.R.),
zoltan.kutalik@unil.ch (Z.K.)

Auwerx et al., 2022, The American Journal of Human Genetics *109*, 647–668
April 7, 2022 © 2022 The Author(s).
https://doi.org/10.1016/j.ajhg.2022.02.010

The individual and global impact of copy-number variants on complex human traits

Chiara Auwerx,[1,2,3,4] Maarja Lepamets,[5,6] Marie C. Sadler,[3,4] Marion Patxot,[2] Miloš Stojanov,[7] David Baud,[7] Reedik Mägi,[6] Estonian Biobank Research Team,[6] Eleonora Porcu,[1,3,4] Alexandre Reymond,[1,*] and Zoltán Kutalik[2,3,4,8,*]

Summary

The impact of copy-number variations (CNVs) on complex human traits remains understudied. We called CNVs in 331,522 UK Biobank participants and performed genome-wide association studies (GWASs) between the copy number of CNV-proxy probes and 57 continuous traits, revealing 131 signals spanning 47 phenotypes. Our analysis recapitulated well-known associations (e.g., 1q21 and height), revealed the pleiotropy of recurrent CNVs (e.g., 26 and 16 traits for 16p11.2-BP4-BP5 and 22q11.21, respectively), and suggested gene functionalities (e.g., *MARF1* in female reproduction). Forty-eight CNV signals (38%) overlapped with single-nucleotide polymorphism (SNP)-GWASs signals for the same trait. For instance, deletion of *PDZK1*, which encodes a urate transporter scaffold protein, decreased serum urate levels, while deletion of *RHD*, which encodes the Rhesus blood group D antigen, associated with hematological traits. Other signals overlapped Mendelian disorder regions, suggesting variable expressivity and broad impact of these loci, as illustrated by signals mapping to Rotor syndrome (*SLCO1B1/3*), renal cysts and diabetes syndrome (*HNF1B*), or Charcot-Marie-Tooth (*PMP22*) loci. Total CNV burden negatively impacted 35 traits, leading to increased adiposity, liver/kidney damage, and decreased intelligence and physical capacity. Thirty traits remained burden associated after correcting for CNV-GWAS signals, pointing to a polygenic CNV architecture. The burden negatively correlated with socio-economic indicators, parental lifespan, and age (survivorship proxy), suggesting a contribution to decreased longevity. Together, our results showcase how studying CNVs can expand biological insights, emphasizing the critical role of this mutational class in shaping human traits and arguing in favor of a continuum between Mendelian and complex diseases.

Introduction

With the advent of genome-wide associations studies (GWASs), the polygenic architecture of complex human traits has become apparent.[1–3] Still, single-nucleotide polymorphisms (SNPs) do not explain the totality of observed phenotypic variability—a phenomenon referred to as "missing heritability"—and one proposed explanation is the contribution of additional types of genetic variants, such as copy-number variants (CNVs).[4]

Characterized by the deletion or duplication of DNA fragments ≥ 50 bases,[5] CNVs represent a highly diverse mutational class that, due to their possibly large size, constitute potent phenotypic modifiers that act through e.g., gene dosage sensitivity, truncation or fusion of genes, unmasking of recessive alleles, or disruption of *cis*-regulatory elements.[6] Hence, CNVs have been acknowledged to play an important role in human diseases and were identified as the genetic etiology of 65 rare and debilitating genomic syndromes by DECIPHER[7] (web resources). However, early GWASs failed to establish clear links between CNVs and complex traits and diseases.[8,9] Several factors, specific to genome-wide copy-number association studies (CNV-GWASs), contributed to these negative results, such as the low frequency and variable breakpoints of CNVs in the population, as well as uncertainty and low resolution of CNV calls originating from genotyping microarrays.[10] In recent years, methodological development, as well as the creation of large biobanks, has allowed bypassing of some of these hurdles. Focusing on a curated set of CNVs, a series of studies characterized the impact of well-established pathogenic CNVs on cognitive performance,[11] physical measurements,[12,13] common medical conditions,[14,15] and blood biomarkers.[16] Alternatively, unbiased genome-wide (GW) studies have been conducted,[17–21] involving loci not covered by targeted approaches and adding to the growing body of evidence implicating CNVs in complex traits. Notably, a recent study made use of the UK Biobank (UKBB)[22] to assess the impact of CNVs on over 3,000 traits, providing the research community with a large population-based CNV-to-phenotype resource.[18] Using an independent CNV calling and association pipeline and focusing on a set of 57 medically relevant continuous traits, we here confirm previously established associations, uncover biological insight through in-depth analysis of particular CNV-trait pairs, and expose a nuanced role of CNVs along

[1]Center for Integrative Genomics, University of Lausanne, Lausanne 1015, Switzerland; [2]Department of Computational Biology, University of Lausanne, Lausanne 1015, Switzerland; [3]Swiss Institute of Bioinformatics, Lausanne 1015, Switzerland; [4]University Center for Primary Care and Public Health, Lausanne 1010, Switzerland; [5]Institute of Molecular and Cell Biology, University of Tartu, Tartu 51010, Estonia; [6]Estonian Genome Centre, Institute of Genomics, University of Tartu, Tartu 51010, Estonia; [7]Materno-fetal and Obstetrics Research Unit, Department Woman-Mother-Child, CHUV, Lausanne 1011, Switzerland
[8]Twitter: @zkutalik
*Correspondence: alexandre.reymond@unil.ch (A.R.), zoltan.kutalik@unil.ch (Z.K.)
https://doi.org/10.1016/j.ajhg.2022.02.010.
© 2022 The Author(s). This is an open access article under the CC BY license (http://creativecommons.org/licenses/by/4.0/).

the rare versus common disease spectrum, suggesting that the deleterious impact of CNVs contributes to decreased longevity in the general population.

Material and methods

Study material

Cohort description

Main analyses were performed in the UKBB, a volunteer-based cohort of ~500,000 individuals (54% females) from the general UK population for which microarray-based genotyping and extensive phenotyping data are available.[22] Participants signed a broad informed consent form and data were accessed through an application (16389) approved by the UKBB. Replication analyses were performed in the Estonian Biobank (EstBB), a population-based cohort of ~200,000 individuals (66% females) for which microarray-based genotyping, body measurements, blood biomarker levels, and medical diagnoses are available.[23] Whole-genome sequencing (WGS) data were available for ~2,500 samples. All participants signed a broad informed consent form and analyses were carried out under ethical approval 1.1-12/624 from the Estonian Committee on Bioethics and Human Research and data release N05 from the EstBB. We used the Lausanne University Hospital (CHUV) maternity cohort, consisting of 5,164 women, to assess the impact of a Rhesus negative (Rh^-) blood group on hematological traits. Approval from the Ethics Committee of Vaud (CER-VD) was obtained for data reusage (ID 2019-00280). Comprehensive cohort description is provided in supplemental material and methods, note 1.

Software versions

CNVs were called with PennCNV v1.0.5[24] with PennCNV-Affy (27/08/2009). CNVs were filtered on the basis of a quality scoring pipeline.[25] Various genetic analyses were conducted with PLINK v1.9 and PLINK v2.0.[26] ANNOVAR (24/10/2019)[27] was used for genome annotation. Meta-analysis was carried out with GWAMA v2.2.2.[28] Statistical analyses were performed with R v3.6.1 and graphs were generated with R v4.0.3.

The CNV landscape of the UK Biobank

Genotype data

Data acquisition and quality control (QC) have been described.[22] Briefly, UKBB participants were genotyped on two similar arrays (95% probe overlap): 438,427 samples (95 batches) were genotyped with the Applied Biosystems UK Biobank Axiom Array (825,927 probes) and 49,950 samples (11 batches) were genotyped with the Applied Biosystems UK BiLEVE Axiom Array by Affymetrix (807,411 probes). All results in this study are based on the human genome reference build GRCh37/hg19.

Sample selection

Related, gender mismatched, high missingness, non-White British ancestry, and retracted samples were excluded (*used.in.pca.calculation* = 0 and *in.white.British.ancestry.subset* = 0 in Sample-QC v2 file). To protect the analysis from somatic chromosomal aberrations, we excluded individuals with self-reported (#20001, codes: 1047, 1048, 1050, 1051, 1052, 1053, 1055, 1056, 1056; UKBB update 03/2020) and/or hospital diagnosed (#41270; International Classification of Diseases, 10th Revision [ICD-10] codes mapping to "cancer of lymphatic and hematopoietic tissue's" exclusion range in the PheCode Map 1.2 [beta], accessed 09/12/2020;[29] UKBB update 08/2019) blood malignancy. CNV outliers were later removed (CNV calling and quality control). All reported results are for 331,522 unrelated White British UKBB participants (54% females).

CNV calling and quality control

Chromosomes 1–22 and pseudoautosomal regions were assumed to have a normal copy-number state (i.e., two) in all individuals, and CNVs were called with standard PennCNV settings in parallel for all 106 genotyping batches. As males are hemizygous for chromosome X, chromosome X CNVs were called separately with the inbuilt PennCNV arguments for chromosome X CNV calling (supplemental material and methods, note 2). CNVs originating from samples genotyped on plates with a mean CNV count per sample > 100 or from samples with >200 CNVs or a single CNV > 10 Mb were excluded, as these might be indicative of batch effects, genotyping errors, or extreme chromosomal abnormalities. To mitigate issues related to high false positive rates and variability in CNV break points, we used a post-PennCNV processing pipeline[17,25] (supplemental material and methods, note 2). First, a quality score (QS) ranging from −1 (likely deletion) to 1 (likely duplication) and reflecting the probability for the CNV to be a true positive was attributed to each PennCNV call. Next, PennCNV coordinates were transformed into per-chromosome *probe* × *sample* matrices; entries reflect the QS attributed to the CNV mapping to these probes. Copy neutral probes are indicated by 0 and individuals with no CNVs were added as all-0 columns.

Converting CNV calls into PLINK format

QS matrices were converted to PLINK binary file sets. Probes with ≥1 high-confidence CNV, stringently defined by $|QS| \geq 0.5$, were retained and encoded into three file sets to accommodate analyses according to a mirror ($PLINK_{CNV}$), duplication-only ($PLINK_{DUP}$), or deletion-only ($PLINK_{DEL}$) association model (--make-bed PLINK v1.9; Table 1).

CNV frequency calculation

Genotype counting was performed for the 740,434 probes stored in $PLINK_{CNV}$ (--freqx PLINK v1.9). 41,670 array-specific probes with genotype count missingness > 5% were excluded and each probe's CNV $(100\times Num_{CNV}/(Num_{CNV} + Num_{non-CNV}))$, duplication $(100\times Num_{dup}/(Num_{CNV} + Num_{non-CNV}))$, and deletion $(100\times Num_{del}/(Num_{CNV} + Num_{non-CNV}))$ frequencies were calculated [%], with $Num_{non-CNV}$, Num_{dup}, and Num_{del}, the number of individuals carrying 2, <2, and >2 copies of that probe, respectively, and $Num_{CNV} = Num_{dup} + Num_{del}$.

CNV association studies in the UK Biobank

CNV probe selection and number of effective tests

Association studies were restricted to probes with a CNV, duplication, or deletion frequency ≥ 0.005% for the mirror, duplication-only, or deletion-only models, respectively. To group probes at the core of CNV regions while retaining variability at breakpoints, we pruned probes at $r^2 > 0.9999$ in $PLINK_{CNV}$, $PLINK_{DUP}$, and $PLINK_{DEL}$ (--indep-pairwise 500 250 0.9999 PLINK v2.0). Retained CNV-proxy probes remained highly correlated and the number of effective tests, N_{eff}, was estimated at 11,804 (supplemental material and methods, note 3),[17,30] setting the GW threshold for significance at $p \leq 0.05/11{,}804 = 4.2 \times 10^{-6}$. Accounting solely for duplications or deletions resulted in lower N_{eff} estimates but the same conservative threshold was used for all models.

Phenotype selection

Fifty-seven continuous traits were selected (supplemental material and methods, note 3). Traits were inverse normal transformed prior correction for sex (except for sex-specific traits), age (#21003), age^2, genotyping batch, and principal components (PCs) 1–40. Normal phenotypic ranges were retrieved and converted from Symed MediCalc (web resources).

Table 1. PLINK encoding of CNVs

Association model	Mirror	Duplication-only	Deletion-only
**PLINK file set**	**$PLINK_{CNV}$**	**$PLINK_{DUP}$**	**$PLINK_{DEL}$**
Deletion ($QS < -0.5$)	AA	00	TT
Copy neutral ($-0.5 \leq QS \leq 0.5$)	AT	AT	AT
Duplication ($QS > 0.5$)	TT	TT	00

Encoding of high-confidence CNVs ($|QS| \geq 0.5$) from quality score (QS) matrices into three PLINK file sets.

Genome-wide copy-number association studies

Associations between the copy number (CN) of selected probes and normalized covariate-corrected traits were performed (--glm omit-ref no-x-sex hide-covar allow-covars PLINK v2.0). To avoid interference between the two-letter CNV encoding (Table 1) and the assumption of male chromosome X hemizygosity, we (falsely) labeled all individuals as female. For sex-specific traits, samples from the opposite sex were excluded. Three association models were applied: the mirror model ($PLINK_{CNV}$) assessed the additive effect of each additional copy of a probe, the duplication-only model ($PLINK_{DUP}$) assessed the impact of a duplication while disregarding deletions, and the deletion-only model ($PLINK_{DEL}$) assessed the impact of a deletion while disregarding duplications. Given CNV encoding (Table 1), effects were homogenized to "T" by multiplying β by −1 when A1 was "A." GW-significant associations ($p \leq 4.2 \times 10^{-6}$; CNV probe selection and number of effective tests) were retained. The number of independent signals per traits was determined by stepwise conditional analysis (supplemental material and methods, note 3). Briefly, CNV genotype of the lead probe was regressed out from the phenotype and association studies were conducted anew until no more GW-significantly associated probes remained.

CNV region definition, merging, and annotation

CNV region (CNVR) boundaries were defined by the most distant probe within ± 3 Mb and $r^2 \geq 0.5$ of each independent lead probe (--show-tags–tag-kb 3000 --tag-r2 0.5 PLINK v1.9). Signals from the different models were merged when involving (1) the same trait, (2) overlapping CNVRs, and (3) directional concordance according to a mirror model. CNVR boundaries were defined as the maximal CNVR and characteristics of the most significant model were retained. CNVRs were annotated with annotate_variation.pl, with hg19 RefSeq gene names (--geneanno; 08/06/2020) and NHGRI-EBI GWAS Catalog[31] (web resources) associations (--regionanno; 27/10/2021) via ANNOVAR. NHGRI-EBI GWAS Catalog trait synonyms considered are listed in Table S1. For each trait, focusing on autosomes, we performed a two-sided binomial test to compare SNP-GWAS signal density (from NHGRI-EBI GWAS Catalog; 27/10/2021) within CNVRs as compared to the entire genome. Number of SNP-GWAS signals falling within trait-associated CNVRs represent successes, total length of trait-associated CNVRs [bp] represent trials, and total number of SNP-GWAS signals divided by the autosomal genome length (2,881,033,286 bp; web resources) represent hypothesized density.

Replication in the Estonian Biobank

Comparative analysis of CNV quality

Quality-controlled Illumina Infinium OmniExpress-24 microarray genotyping and WGS data were available for 966 overlapping and unrelated samples of the EstBB (supplemental material and methods, note 4). Microarray-based autosomal CNVs were called with PennCNV and samples with >200 CNVs were excluded. PennCNV calls were attributed a QS and filtered for $|QS| \geq 0.5$, following a procedure analogous to the one described for the UKBB. We called WGS-based autosomal CNVs by using the Genome STRiP pipeline[32] and merged adjacent CNVs (gap ≤ 20% of merged CNV length) to mimic the PennCNV protocol. For both methods, we excluded duplications and deletions smaller than 1 kb and 2 kb, respectively, and larger than 10 Mb. For each of the 709,358 genotyped probes, a cross-sample PennCNV-CNV profile was constructed, taking values of −1 (deletion), 0 (copy neutral), and 1 (duplication). Similar profiles were constructed on the basis of STRiP-CNV calls and Pearson's coefficient of correlation and number of CNV carriers according to both methods were calculated for each genomic location. For probes with ≥1 PennCNV call but no STRiP call, all correlated probes ($r \geq 0.5$, according to PennCNV profiles) within ± 250 kb were retrieved and maximal PennCNV-WGS correlation among these probes was retained. Analyses were repeated on a subset of 5,566 probes overlapping UKBB-trait-associated CNVRs.

Phenotype data

Analyzed traits were queried in the EstBB: height, weight, and body mass index (BMI) were collected at enrollment; age at menarche and menopause were collected by project-based questionnaires; 41 traits were retrieved from parsed notes in health registries; 11 did not have any corresponding term. Because most phenotypic measurements originate from health registries, they were gathered at different time points and by different practitioners and were only available for a limited subset of participants. In case of repeated measurement, the most recent one was retained. Traits with sample size ≥ 2,000 were selected and inverse normal transformed prior correction for sex (except for sex-specific traits), age, age^2, genotyping batch, and PCs 1–20.

CNV calling and copy-number association studies

We used quality-controlled Illumina Global Screening Array (GSA) genotype data (supplemental material and methods, note 4) to call autosomal CNVs for 193,844 individuals. CNVs were attributed a QS and encoded into three PLINK binary file sets, following the procedure described for the UKBB. CNV, duplication, and deletion frequencies among the 89,516 unrelated samples remaining after QC (supplemental material and methods, note 4) were calculated for 671,035 probes and association studies were run as previously described for the UKBB. Using the most significant association model for the 131 merged UKBB signals, we selected the most significantly associated EstBB probe within the boundaries of the UKBB-defined CNVR. EstBB p values were adjusted to account for directional concordance with UKBB effects: in case of direction agreement, $p_{new} = \frac{p_{old}}{2}$, otherwise $p_{new} = 1 - \left(\frac{p_{old}}{2}\right)$. Sufficient genomic variability and phenotypic data were available to assess replication of 61 out of 131 signals, setting the replication threshold for significance at $p \leq 0.05/61 = 8.2 \times 10^{-4}$. We conducted simulations to estimate the power of our replication study

assuming effect sizes similar to those observed in the UKBB and CNV frequencies and sample sizes reflective of the EstBB (supplemental material and methods, note 4). For each signal, 10,000 simulations were conducted. Power was defined as the fraction of non-missing p values $\leq 8.2 \times 10^{-4}$. Expected number of replications was estimated as the average power across assessed signals multiplied by the number of assessed signals.

Extended phenotypic assessment

To assess patients' disease status, ICD-10 codes were used (#41270). Self-reported high alcohol consumption (#1558) and γ-glutamyl transferase (GGT)-increasing drug usage (#20003) were evaluated as potential lifestyle confounders of the 22q11.23-GGT association. Six socio-economic factors and life history traits were additionally considered in the burden analysis. Traits were inverse normal transformed prior to correction for sex, age (#21003), age^2, genotyping batch, and PCs 1–40, except for "age at recruitment", which was not corrected for age and age^2. Exact definitions are found in supplemental material and methods, note 5.

RHD and hematological traits

Transcriptome-wide Mendelian randomization

Using univariable transcriptome-wide Mendelian randomization[33] (TWMR), we estimated the causal effects of differential *RHD* and *RSRP1* expression on reticulocyte count, platelet count, and glycated hemoglobin (HbA1c; supplemental material and methods, note 6). Robustness was ascertained by excluding rs55794721, which had an extreme effect on both exposures and outcomes.

Association between Rh blood group and hematological traits

Impact of Rh$^-$ blood group on platelet count, reticulocyte count, and HbA1c levels was assessed in the CHUV maternity cohort through multivariate linear regression that incorporates the covariates: age at measurement, gestational week at measurement, whether the woman was pregnant at measurement, and whether the woman had a child prior to the measurement (supplemental material and methods, note 6). One-sided p values were calculated as $p_{new} = \frac{p_{old}}{2}$ in case of directional agreement with the UKBB effect.

CNV burden analyses in the UK Biobank

CNV burden calculation

An individual's CNV burden was defined as the number of Mb or genes affected by high-confidence autosomal CNVs ($|QS| \geq 0.5$). For the latter, we retained CNVs overlapping exons, splice sites, non-coding RNA, 3′UTR, and 5′UTR (CNV region definition and annotation) to assess number of disrupted genes. Duplication and deletion burdens were calculated similarly, and correlation between the six metrices was assessed with Pearson's coefficient of correlation. We used two-sided unpaired Wilcoxon rank-sum test to assess differences in CNV burden between males and females.

CNV burden analysis

Linear regressions were performed between burden metrices and the same 57 normalized, covariate-corrected traits investigated by GWAS. For sex-specific traits, samples from the opposite sex were excluded. We set the significance threshold at $p \leq 0.05/63 = 7.9 \times 10^{-4}$ to account for six additional life history traits (supplemental material and methods, note 5). Linear regressions were computed between non-normalized, covariate-corrected "mother's and father's age at death" and the burden to get effects on the years/[Mb or gene] scale. We meta-analyzed results with GWAMA to assess impact on parental lifespan.

Burden analysis correction for modifier CNVRs

To assess the impact of the CNV burden on a trait, we collected CNVRs associating with that trait under the mirror model into a *sample* × *CNVR* matrix G. G Takes a value of −1 or 1 if the sample carries a CNVR-overlapping (≥1 bp) deletion or duplication, respectively, and 0 otherwise. G was regressed out of the trait and burdens were adjusted by subtracting the number of Mb or genes affected by CNVR-overlapping CNVs before performing associations anew. For the duplication and deletion burdens, CNVRs found through the duplication-only and deletion-only models, respectively, were considered and CNVR-overlapping deletions and duplications, respectively, were set to 0 in G.

Fraction of inherited CNVs

Rate of CNV inheritance was estimated by examining the fraction of shared CNVs among siblings pairs defined by kinship coefficient 0.2–0.3 and fraction of SNPs with identity by state at $0 \geq 0.0012$.[22] We retained 16,179 pairs with one individual among samples selected for the main CNV-GWASs (Sample selection). Shared CNVs were defined as high-confidence duplications ($QS \geq 0.5$) or deletions ($QS \leq -0.5$) on the same chromosome with ≥25 kb overlap. For each pair, we calculated the fraction of CNVs the individual in the main analysis shared with his/her sibling (number of shared CNVs/total number of CNVs in that individual) and averaged the results over all pairs to obtain the mean fraction of shared CNVs. As a control, the analysis was repeated by pairing the 16,179 individuals from the main analysis with random individuals sampled without replacement from the main pool of individuals.

Results

The CNV landscape of the UK Biobank

We used PennCNV[24] to call autosomal, pseudoautosomal, and chromosome X CNVs in 332,935 unrelated White British UKBB participants with no reported blood malignancy. Calls were processed by a pipeline that excluded 1,413 CNV outlier samples and attributed a probabilistic QS to each CNV.[25] Out of 1,329,290 identified CNVs, 176,870 high-confidence CNVs with $|QS| \geq 0.5$ were retained for follow-up analyses (Figure S1A). As the fraction of homozygous CNVs (CN 0 or 4) was negligeable (1.1%; Figure S1B), we define deletions and duplications as having a CN smaller or larger than two, respectively, for the remainder of this study. Duplication length varied between 366 bp and the upper boundary, set at 10 Mb (17–3,968 probes), with a median of 297 kb (133 probes), and deletion length between 217 bp and 10 Mb (8–4,017 probes), with a median of 137 kb (60 probes) (Figures S1C and S1D). Overall, 129,263 (39%) participants carried at least one high-confidence CNV and 34,804 (10%) carried more than one (Figure S1E). In samples with ≥1 CNV, the total length of affected bases ranged between 217 bp and 14.2 Mb, with a median of 292 kb (Figure S1F). Analyzing the global CNV burden of the cohort, 70% was caused by duplications, which were both more numerous (54%) and 213 kb longer, on average, than deletions (Figures S1B–S1D). No differences in CNV burden, measured as the number of Mb or genes affected by CNVs, was detected across sexes (two-sided, unpaired Wilcoxon rank-sum test: $p_{Mb} = 0.793$; $p_{Genes} = 0.748$). This contrasts

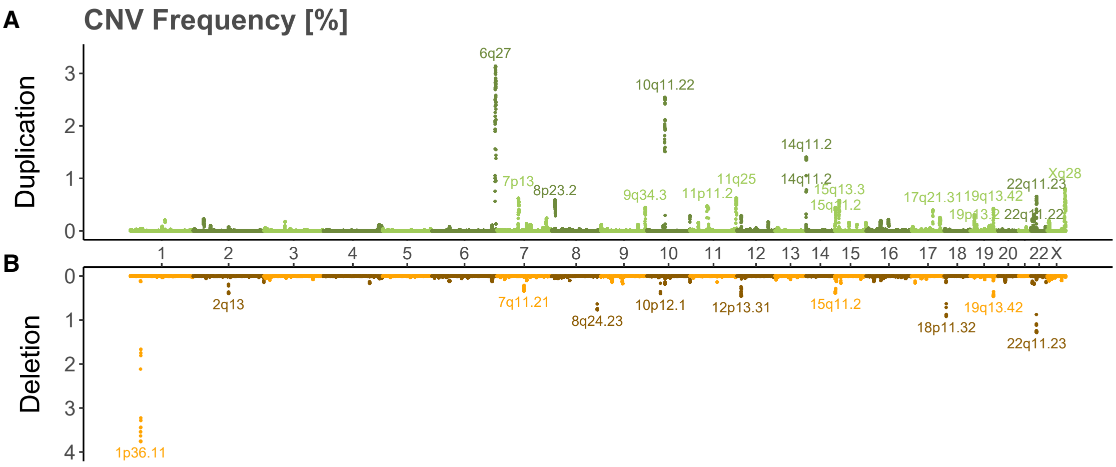

Figure 1. CNV frequency landscape in the UK Biobank
(A and B) Miami plot of high-confidence probe-level duplication (A) and deletion (B) frequencies [%] in the UKBB. Consecutive probes with identical duplication and deletion frequencies were clustered so that each dot represents one probe group. Loci with duplication frequency $\geq$ 0.3% or deletion frequency $\geq$ 0.2% are labeled with cytogenic bands.

with the excess of deleterious CNVs reported in females with neuro-psychiatric/developmental disorders,[34–37] suggesting that this observation is trait dependent.

To bypass issues related to inter-individual variability in recurrent CNV break points, we transformed CNV calls to the probe level for frequency calculation.[17] A large fraction of the genome was subjected to CNVs as 662,247 probes (82%) were found in a CN-altered state in at least one participant, even if 81% of these had a CNV frequency $\leq$ 0.005% ($n \leq 16$). The fraction of never-deleted probes (43%) was 1.7× higher than the fraction of never-duplicated probes (26%), and with some notable exceptions, deletion frequencies tended to be lower than duplication frequencies (Figure 1). For most loci with high CNV frequency, duplication and deletion frequencies did not mirror each other (Figure 1). Overall, these results are in line with the common paradigm that CNVs are individually rare but collectively common.[18,38,39]

The pleiotropic impact of recurrent CNVs

To assess the phenotypic impact of the UKBB CNV landscape, we selected 57 medically relevant phenotypes—including anthropometric traits, cardio-pulmonary assessments, hematological measurements, blood biomarkers, neuronal functions, and sex-specific attributes—with presumed high heritability (Table S1; Figure S2A). GWASs were performed between the CN of pruned ($r^2 > 0.9999$) CNV-proxy probes with a CNV, duplication, and deletion frequency $\geq$ 0.005% and aforementioned traits according to a mirror (28,257 probes; Figure 2A), duplication-only (14,070 probes; Figure 2B), and deletion-only (9,936 probes; Figure 2C) association model, respectively. As the number of statistical tests is much lower than for classical SNP-GWASs and retained probes remain highly correlated due to the recurrent nature and large size of assessed CNVs, we calculated the number of effective (i.e., independent) tests, setting the GW threshold for significance at $p \leq 0.05/11{,}804 = 4.2 \times 10^{-6}$ (material and methods). Stepwise conditional analysis narrowed signals down to 86, 50, and 68 GW-significant associations for the mirror, duplication-only, and deletion-only models, respectively, of which 45, 22, and 32 reached the conventional SNP-GWAS threshold of $p \leq 5 \times 10^{-8}$. These signals were combined into 131 independent associations spanning 47 phenotypes (Figure 2D; Table S2; 62 signals across 32 phenotypes at $p \leq 5 \times 10^{-8}$). Following previous works,[17,18,21] we omitted accounting for the number of assessed traits, but even with a stringent experiment-wide threshold for significance ($p \leq 0.05/(11{,}804 \times 57) = 7.4 \times 10^{-8}$), 68 out of 131 (52%) CNV-GWAS signals remained significantly associated. All summary statistics are made available (data and code availability).

Among signals identified through the mirror model, 63 (73%) replicated with either type-specific model, often reflecting the most common CNV type (Figure 2D, top). Five (6%) signals replicated with both type-specific models, providing examples of "true mirror" effects (i.e., opposite impact of duplications and deletions), such as the association between height and the CN of a Xp22.33 pseudoautosomal CNVR (chrX: 285,850–1,720,422; $\beta_{mirror} = 2.33$ cm; $p = 7.2 \times 10^{-36}$; Figure 2E) encompassing the short-stature homeobox gene *SHOX* (MIM: 312865). This association aligns with the short stature observed in individuals suffering from Turner syndrome (i.e., females with partial or complete loss of one chromosome X) and *SHOX* deficiency disorders (Leri-Weill dyschondrosteosis [MIM: 127300]; Langer mesomelic dysplasia [MIM: 249700]; idiopathic short stature [X-linked] [MIM: 300582]).[40,41] Less established is the impact of increased CN of *SHOX* and/or its regulatory regions,[42] which we found to be associated with tall stature. CN and deletion of overlapping CNVRs further associated with waist-to-hip ratio (WHR) adjusted for BMI (chrX: 514,930–618,611;

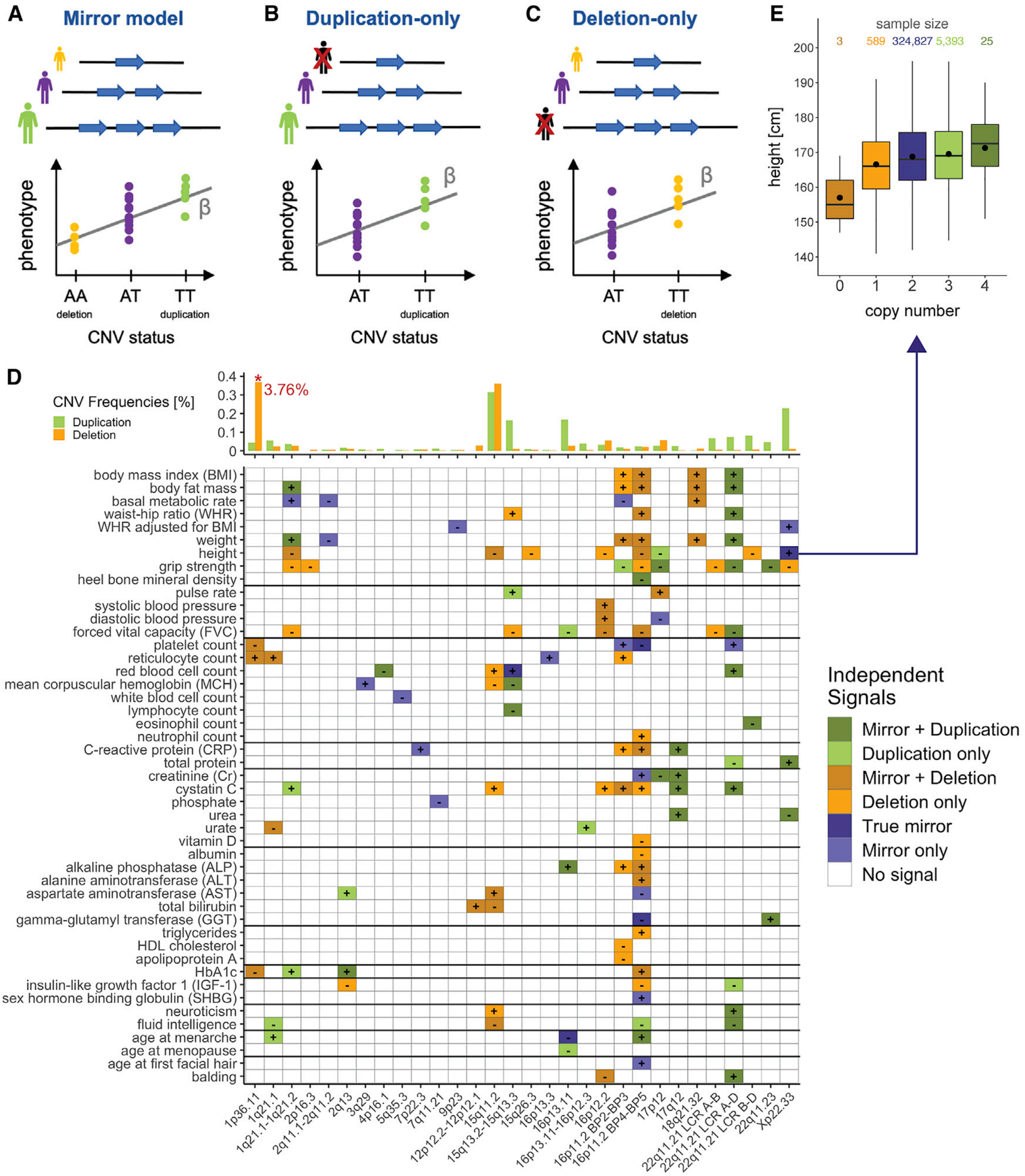

Figure 2. CNV-GWAS roadmap of the UK Biobank

(A–C) CNV-GWAS association models with PLINK encoding: the mirror model assumes equal-sized but opposite-direction effect of deletion and duplication and estimates the impact of each additional copy (A); the duplication-only model disregards deletion carriers and estimates the effect of duplications (B); the deletion-only model disregards duplication carriers and estimates the effect of deletions (C).
(D) Independent genome-wide significant associations ($p \leq 0.05/11{,}804 = 4.2 \times 10^{-6}$) between CNV regions (x axis; as cytogenic bands) and traits (y axis). Color tiles represent the model(s) through which the association was detected—dark green, mirror and duplication-only; light green, duplication-only; dark orange, mirror and deletion-only; light orange, deletion-only; dark purple, mirror, duplication-only, and deletion-only; light purple, mirror; white: none—and signs show directionality, so that the duplication (greens), deletion (oranges), or copy number (purples) of a CNV region associated with a phenotypic increase (+) or decrease (–). 16p11.2 (16p11.2 BP2-BP3 and 16p11.2 BP4-BP5) and 22q11.21 recurrent CNVs (LCR B at chr22: 20,400,000) are assessed separately. For each CNV region, average duplication (green) and deletion (orange) frequencies [%] of the lead probe (according to the most significant model) are depicted at the top. Deletion frequency of 1p36.11 was truncated from 3.76%.
(E) Boxplot representing height in individuals with CNVs overlapping the Xp22.33 pseudoautosomal region (chrX: 285,850–1,720,422). Sample size is reported for each copy-number category at the top; boxes show the first (Q1), second (median, thick line), and third (Q3) quartiles; lower and upper whiskers show the most extreme value within Q1 minus and Q3 plus 1.5× the interquartile range, respectively; dots show the mean; outliers are not shown.

$\beta_{mirror} = 0.12$ SD; $p = 2.3 \times 10^{-6}$) and hand grip strength (chrX: 762,346–2,219,659; $\beta_{del} = -4.73$ kg; $p = 3.7 \times 10^{-7}$), respectively. While skeletal muscle hypertrophy has been reported in patients with Leri-Weill dyschondrosteosis,[43] we hypothesize that the reduced grip strength in deletion carriers might result from the Madelung deformity characterizing the disorder, which is known to cause wrist pain and decreased grip strength,[44] and/or the correlation between grip strength and height (Figure S2A). Unlike mirror effects, partially overlapping signals between decreased forced vital capacity or grip strength and the 22q11.21 low copy repeat (LCR) A-B (chr22: 19,024,651–20,311,646; deletion-only) and 22q11.21 LCR A-D (chr22: 19,024,651–21,407,523; mirror and duplication-only) hinted at U-shaped effects (i.e., deletion and duplication shift the phenotype in the same direction) (MIM: 188400 and 192430), demonstrating the existence of different mechanisms of gene dosage (Figure 2D).

Most signals involved large recurrent CNVRs (mean = 901 kb; median = 612 kb) and we confirm multiple well-established associations, such as the negative impact of the 1q21.1–1q21.2 deletion (MIM: 612474) on height[45–47] (chr1: 146,478,785–147,832,715; $\beta_{del} = -6.67$ cm; $p = 2.5 \times 10^{-21}$), the negative correlation between BMI and the CN of 16p11.2 BP4-BP5 (MIM: 611913 and 614671) (chr16: 29,596,230–30,208,637; $\beta_{del} = 6.11$ kg/m^2; $p = 3.6 \times 10^{-29}$)[48–50] and 16p11.2 BP2-BP3 (MIM: 613444) (chr16: 28,818,541–29,043,450; $\beta_{del} = 4.25$ kg/m^2; $p = 5.3 \times 10^{-8}$),[49,51,52] or the more recently reported positive association between 16p11.2 BP4-BP5's CN and age at menarche (chr16: 29,596,230–30,208,637; $\beta_{mirror} = 1.16$ years; $p = 1.2 \times 10^{-10}$).[53] In addition, our results revealed the broad pleiotropic impact of these loci: 26, 16, and 12 traits associated with the 16p11.2 BP4-BP5, 22q11.21, or 16p11.2 BP2-BP3 regions, respectively. Some of these previously poorly described associations might help shed light on the molecular mechanisms linking involved loci to phenotypes, as exemplified with the association between the 16p11.2 BP4-BP5 deletion (chr16: 29,596,230–30,208,637) and reduced levels of insulin-like growth factor 1 (IGF-1; $\beta_{del} = -3.26$ nmol/L; $p = 2.9 \times 10^{-7}$). In children, diseases characterized by low levels of IGF-1 (e.g., IGF-1 deficiency [MIM: 608747], Laron syndrome [MIM: 262500], or growth hormone [GH] deficiencies [MIM: 262400, 612781, 173100, 307200, 618157, and 615925]) typically result in short stature (proxied by height), while symptoms of adult GH deficiency include increased adipose mass (proxied by BMI, body fat mass, weight, and WHR), decreased muscle mass and strength (proxied by grip strength), altered lipid profile (proxied by triglycerides), and insulin resistance (proxied by HbA1c),[54] all of which are affected in a directionally concordant fashion by the 16p11.2 BP4-BP5 deletion. Conversely, some regions only associated with a single trait, e.g., the CN of a 3q29 region (chr3: 195,725,157–196,035,229) associated with increased mean corpuscular hemoglobin (MCH; $\beta_{mirror} = 1.92$ pg; $p = 1.1 \times 10^{-9}$), whose levels indirectly reflect iron load in erythrocytes.[55] The CNVR harbors the transferrin receptor gene, *TFRC* (MIM: 190010), which is involved in cellular iron uptake and was shown to associate with MCH through SNP-GWAS.[56] Together, these results emphasize the potent role of CNVs as phenotypic modifiers.

Replication in the Estonian Biobank

We next assessed our ability to detect CNVs and sought to replicate identified signals in an independent cohort, the EstBB.[23] Taking advantage of 966 unrelated samples with both microarray-based (PennCNV) and WGS-based (STRiP) CNV calls, we calculated the correlation between the CNV profiles obtained with these two methods for 709,358 quality-controlled, autosomal probes (Figure S3A). Due to small sample size, most probes (630,819 probes; 89%) were monomorphic. Among the 20,963 probes detected in a CNV state in at least one sample by both methods, 71% (14,976 probes; 2.1% of all probes) showed high ($r \geq 0.75$) agreement in calling profiles. We detected 39,847 (5.6%) apparent false positives (i.e., probes only detected in a CNV state by PennCNV). Forty percent of these were in linkage disequilibrium (± 250 kb and $r \geq 0.5$) with probes showing high microarray-WGS concordance (Figure S3B), suggesting that they are true positives mislabeled as false positives due to fragmentation of STRiP CNV calls. We also observed 17,717 (2.5%) false negatives (i.e., probes only detected in a CNV state by STRiP). Size distribution—both in number of base pairs (Figure S3C) and probes (Figure S3D)—of consecutive stretches of false negative probes was smaller than for the other assessed categories, confirming the poor ability to detect small CNVs with microarray data.[10] If false negatives hinder discovery, they do not affect validity of detected associations. We next repeated the analysis on 5,566 probes overlapping UKBB trait-associated CNVRs (Figure S3E) and observed (1) an increased fraction of highly correlated probes (1,431 probes; 71% → 85%), (2) an increased fraction of apparently mislabeled false positives in linkage disequilibrium with highly correlated probes (1,061 probes; 40% → 92%; Figure S3F), and (3) a decreased proportion of false negatives among non-monomorphic probes (215 probes; 23% → 7%), indicating good sensitivity and specificity to detect CNVs at trait-associated genomic loci.

To replicate association signals, microarray-based CNV data were available for 89,516 unrelated individuals. Phenotypic measurements, originating from national health registries, were only available for a limited subset of participants, ranging from ~60,000 for anthropometric measurements, to <1,000 for specialized biomarkers (Table S1). Restricting ourselves to autosomal signals with sample size ≥2,000 and ≥1 CNV carrier, data were available for 61 (47%) CNVR-trait pairs (Table S2; Figure 3A). Six signals replicated with Bonferroni correction for multiple testing ($p \leq 0.05/61 = 8.2 \times 10^{-4}$; Figure 3B) and we observed 7.2× more nominally significant signals than expected by chance (22 signals; two-sided binomial test: $p = 7.8 \times 10^{-14}$; Figure S3G). Effect size estimates

A

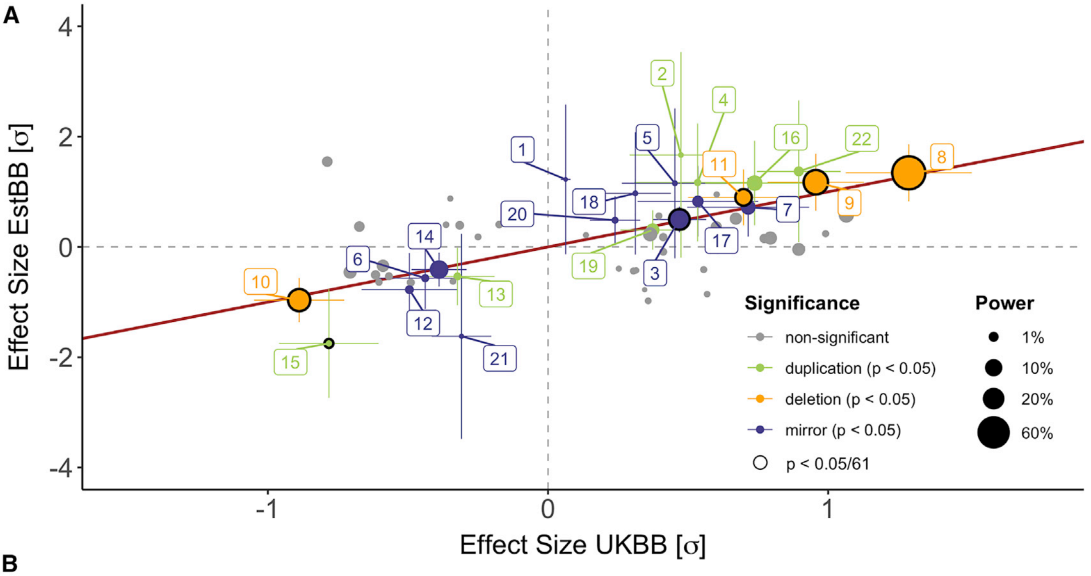

B

	Cytogenic Band (CNV region)	Trait	Effect	UK Biobank β	UK Biobank p	UK Biobank Del – Neutral –Dup	Estonian Biobank β	Estonian Biobank p	Estonian Biobank Del – Neutral –Dup
3	**1q21.1-1q21.2** (1:146,478,785-147,832,715)	height	cm/CN	4.3	$7.8x10^{-22}$	93 – 330,619 – 125	4.6	$3.3x10^{-5}$	11 – 67,844 – 78
8	**16p11.2 BP4-BP5** (16:29,596,230-30,208,637)	BMI	kg/m²/del	6.1	$3.6x10^{-29}$	75 – 330,321 – 101	7.0	$3.4x10^{-7}$	12 – 67,877 – 21
9		WHR	unit/del	0.1	$1.5x10^{-27}$	75 – 330,750 – 101	0.1	$7.6x10^{-6}$	9 – 59,381 – 16
10		height	cm/del	-8.2	$3.3x10^{-27}$	76 – 330,660 – 101	-9.0	$2.5x10^{-6}$	12 – 67,900 – 21
11		weight	kg/del	11.1	$5.0x10^{-12}$	75 – 330,431 – 101	15.2	$5.2x10^{-4}$	12 – 67,891 – 21
15	**17p12** (17:14,098,277-15,468,444)	Cr	μmol/L/dup	-13.8	$6.5x10^{-18}$	196 – 316,488 – 102	-94.1	$5.2x10^{-4}$	4 – 16,247 – 5

Figure 3. Replication of CNV-GWAS signals in the Estonian Biobank
(A) Estonian Biobank (EstBB; y axis) versus UK Biobank (UKBB; x axis) standardized effect sizes. The identity line is in red; size reflects power at $\alpha = 0.05/61$; non-significant signals ($p > 0.05$) are in gray; nominally significant signals ($p \leq 0.05$) with 95% confidence intervals are colored according to replication models: mirror (purple), duplication-only (green), or deletion-only (orange); multiple-testing correction surviving signals ($p \leq 8.2 \times 10^{-4}$) are circled in black and listed in (B) with the first column's color corresponding to the association model and numbers matching labels in (A).
(B) Effect sizes (β; unit in the effect column) and p values (p) for the UKBB and EstBB GWAS, along with the number of individuals with available phenotypic data carrying a deletion, no CNV, or a duplication overlapping the CNV region. Labels indicate: (1) platelet count—1p36.11 (chr1: 25,599,041–25,648,747); (2) glycated hemoglobin (HbA1c)—1q21.1–1q21.2 (chr1: 146,478,785–147,832,715); (3) height—1q21.1–1q21.2 (chr1: 146,478,785–147,832,715); (4) age at menarche—1q21.1 (chr1: 145,368,664–145,738,611); (5) platelet count—16p11.2 BP2-BP3 (chr16: 28,818,541–29,043,450); (6) weight—16p11.2 BP2-BP3 (chr16: 28,818,541–29,043,450); (7) age at menarche—16p11.2 BP4-BP5 (chr16: 29,596,230–30,208,637); (8) body mass index (BMI)—16p11.2 BP4-BP5 (chr16: 29,596,230–30,208,637); (9) waist-to-hip ratio (WHR)—16p11.2 BP4-BP5 (chr16: 29,596,230–30,208,637); (10) height—16p11.2 BP4-BP5 (chr16: 29,596,230–30,208,637); (11) weight—16p11.2 BP4-BP5 (chr16: 29,596,230–30,208,637); (12) alanine aminotransferase (ALT)—16p11.2 BP4-BP5 (chr16: 29,624,931–30,208,637); (13) age at menopause—16p13.11 (chr16: 15,151,451–16,308,285); (14) age at menarche—16p13.11 (chr16: 15,120,501–16,308,285); (15) serum creatinine (SCr)—17p12 (chr17: 14,098,277–15,468,444); (16) SCr—17q12 (chr17: 34,797,651–36,249,489); (17) C-reactive protein (CRP)—17q12 (chr17: 34,797,651–36,249,489); (18) platelet count—22q11.21 LCR A-D (chr22: 19,024,651–21,174,444); (19) BMI—22q11.21 LCR A-D (chr22: 19,024,651–21,463,515); (20) weight—22q11.21 LCR A-D (chr22: 19,024,651–21,463,545); (21) eosinophil count—22q11.21 LCR B-D (chr22: 20,457,855–21,463,545); (22) γ-glutamyl transferase (GGT)—22q11.23 (chr22: 23,688,345–24,990,213).

followed closely the ones detected in the UKBB (Figure 3). Given the low sample sizes, we conducted simulations to assess the power of the replication study. Assuming effect sizes matching those observed in the UKBB, the average replication power was estimated at 5.5% ($\alpha = 0.05/61$; Figure S3H). This corresponds to an expected number of replicated signals of 3.4, slightly below the six observed, and argues in favor of the robustness of the original UKBB CNV-GWAS findings.

CNVs as modifiers of complex traits

To assess whether CNV-GWAS signals mapped to regions previously identified by SNP-GWASs for the same trait, we annotated CNVRs with associations reported by the NHGRI-EBI GWAS Catalog.[31] From the 126 autosomal CNV associations considered, 48 (38%) harbored a SNP signal for the same trait (Table S2). A similar fraction (31%) of CNV-GWAS signals with $4.2 \times 10^{-6} \geq p \geq 5 \times 10^{-8}$ is supported by SNP-GWAS signal, backing the

reliability of intermediate-significant associations. We further tested whether SNP-GWAS signal distribution was denser within trait-associated CNVRs, as compared to the rest of the genome. While this was the case for nine traits (two-sided binomial test: $p \leq 0.05/56 = 8.9 \times 10^{-4}$; Table S3), enrichment did not seem to depend on the type of trait, total number of SNP-GWAS signals (Figure S2B), or length of trait-associated CNVRs (Figure S2B, insert). Nevertheless, colocalization of SNP and CNV signals reinforces confidence that involved loci play a role in shaping associated traits, as illustrated with four examples.

The first example relates to a 1.7 Mb 2q13 CNV (chr2: 111,398,266–113,115,598). Deletion of the region associated with decreased IGF-1 ($\beta_{del} = -5.67$ nmol/L; $p = 6.3 \times 10^{-10}$), an important regulator of glucose and insulin metabolism,[57] and duplication associated with increased HbA1c ($\beta_{dup} = 3.47$ mmol/mol; $p = 1.4 \times 10^{-7}$). The interval encompassed an IGF-1-associated intronic *ACOXL* SNP[20] upstream of *BCL2L11* (MIM: 603827) and two HbA1c-associated SNPs[20,58] downstream of *BCL2L11*. These SNP signals were reported in 2021, indicating that with increased statistical power, signal colocalization will increase. Both traits have not been thoroughly assessed in carriers of the recurrent reciprocal 2q13 CNV, who present with neuro-developmental/psychiatric disorders, dysmorphisms, congenital heart disorder, hypotonia, seizures, micro-/macrocephaly, and microphallus at variable penetrance and expressivity;[59–63] the two latter features are reminiscent of growth defects potentially mediated by dysregulation of the GH/IGF-1/insulin axis. Multiple genes overlapping the CNVR play a role in cell cycle (*BUB1* [MIM: 602452], *ANAPC1* [MIM: 608473]), cell survival (*MERTK* [MIM: 604705]), and apoptosis (*BCL2L11*) regulation; *BCL2L11* is negatively regulated by IGF-1.[64] Our data support the variable penetrance and expressivity of this CNV—not listed as a DECIPHER CNV syndrome—and prompts follow-up studies to confirm and refine understanding of the genetic mechanisms linking the locus to the associated phenotypes.

The second example links the 382 kb 1q21.1 deletion (MIM: 274000) to decreased serum urate levels (chr1: 145,383,239–145,765,206; $\beta_{del} = -48.32$ μmol/L; $p = 5.8 \times 10^{-13}$; Figure 4A). The rearranged interval encompasses 15 genes (Figure S4), including *PDZK1* (MIM: 603831), which encodes a urate transporter scaffold protein[65] and was associated with serum urate levels by SNP-GWASs.[66–69] Recently, *in vitro* experiments elucidated the mechanism through which the urate-increasing T allele of rs1967017 leads to increased *PDZK1* expression,[70] while the *PDZK1* protein-truncating variant rs191362962 was found to associate with decreased serum urate,[20] both suggesting that decreased *PDZK1* expression—an expected outcome of *PDZK1* deletion—decreases serum urate levels. Dividing deletion carriers into groups harboring a full (start < 145.6 Mb) or a partial (start ≥ 145.6 Mb) deletion revealed that the small deletion, encompassing *PDZK1* and three other genes (Figure S4), was sufficient to alter serum urate levels (two-sided t test: $p = 0.92$; Figure 4A).

The third example involves a 1.3 Mb long 22q11.23 duplication and increased GGT (chr22: 23,688,345–24,990,213; $\beta_{dup} = 37.2$ U/L; $p = 9.3 \times 10^{-32}$; Figure 4B). The region harbors several independent GGT SNP-GWAS signals[20,71–76] (MIM: 612365) and five genes involved in glutathione metabolism (KEGG pathway hsa00480), including *GGT1* (MIM: 612346) and *GGT5* (MIM: 137168) (Figure S5A), suggesting that an additional copy of these genes associates with increased levels of the encoded protein. As multiple factors can elevate GGT levels,[77] we used binomial tests to verify that the 180 duplication carriers were not enriched for GGT-altering drug usage ($p = 0.55$), high alcohol consumption ($p = 0.85$), heart failure ($p = 0.23$), or cancer ($p = 1$) and other diseases ($p = 0.64$) of the liver, gallbladder, and bile ducts, as compared to control individuals. Visualization of GGT levels in individuals with two or three copies of the CNVR showed that the 22q11.23 duplication increased serum GGT independently of and additively to other GGT-increasing factors (Figures S5B–S5F).

Finally, we focused on the most frequent CNV in our cohort (frequency = 3.76%; Figure 1A), the 50 kb 1p36.11 deletion (chr1: 25,599,041–25,648,747), which encompasses *RHD* (Rhesus [Rh] blood group D antigen [MIM: 111680]) and *RSRP1* and associated with increased reticulocyte count ($\beta_{del} = 2.7 \times 10^9$ cells/L; $p = 7.8 \times 10^{-14}$), decreased platelet count ($\beta_{del} = -3.7 \times 10^9$ cells/L; $p = 1.4 \times 10^{-12}$), and decreased HbA1c ($\beta_{del} = -0.3$ mmol/mol; $p = 9.3 \times 10^{-8}$) (Figure 4C). Overlap with SNP-GWAS signals for various hematological traits[78,79] combined with subsequent replication of the reticulocyte count association based on whole-exome sequencing CNV calls[80] prompted the investigation of the expression of these genes in whole blood. Tissue-specific transcriptomic data from the GTEx project v8[81] (web resources) revealed that *RHD*, a protein whose presence/absence on erythrocyte cell membranes is critical in determining an individual's Rh blood group,[82] was almost exclusively expressed in whole blood (Figure 4D), whereas *RSRP1* was ubiquitously expressed, with lower expression in whole blood (Figure 4E). Selecting *RHD*'s (ENST00000328664) and *RSRP1*'s (ENST00000243189; Figure S6A) most highly expressed isoforms in whole blood, we mapped exons to the association plot, showing that *RSRP1*'s isoform does not overlap the CNVR, in contrast to *RHD*'s, which is fully encompassed by it (Figure 4C). We next used transcriptome-wide Mendelian randomization[33] (TWMR; Table S4) to establish a directionally concordant causal link between *RHD* expression and reticulocyte count ($\alpha_{TWMR} = -0.013$, $p = 1.6 \times 10^{-4}$; Figure S6B), platelet count ($\alpha_{TWMR} = 0.031$, $p = 2.3 \times 10^{-9}$; Figure S6C), and HbA1c levels ($\alpha_{TWMR} = 0.017$, $p = 3.5 \times 10^{-7}$; Figure S6D). *RSRP1* TWMR resulted in directionally concordant and significant effects, but the gene had suboptimal number of instruments (three) for robust causal inference (Figures S6E–S6G). Furthermore, both genes' signals were driven by a strong upstream expression quantitative locus (rs55794721; Figures S6B–S6G).

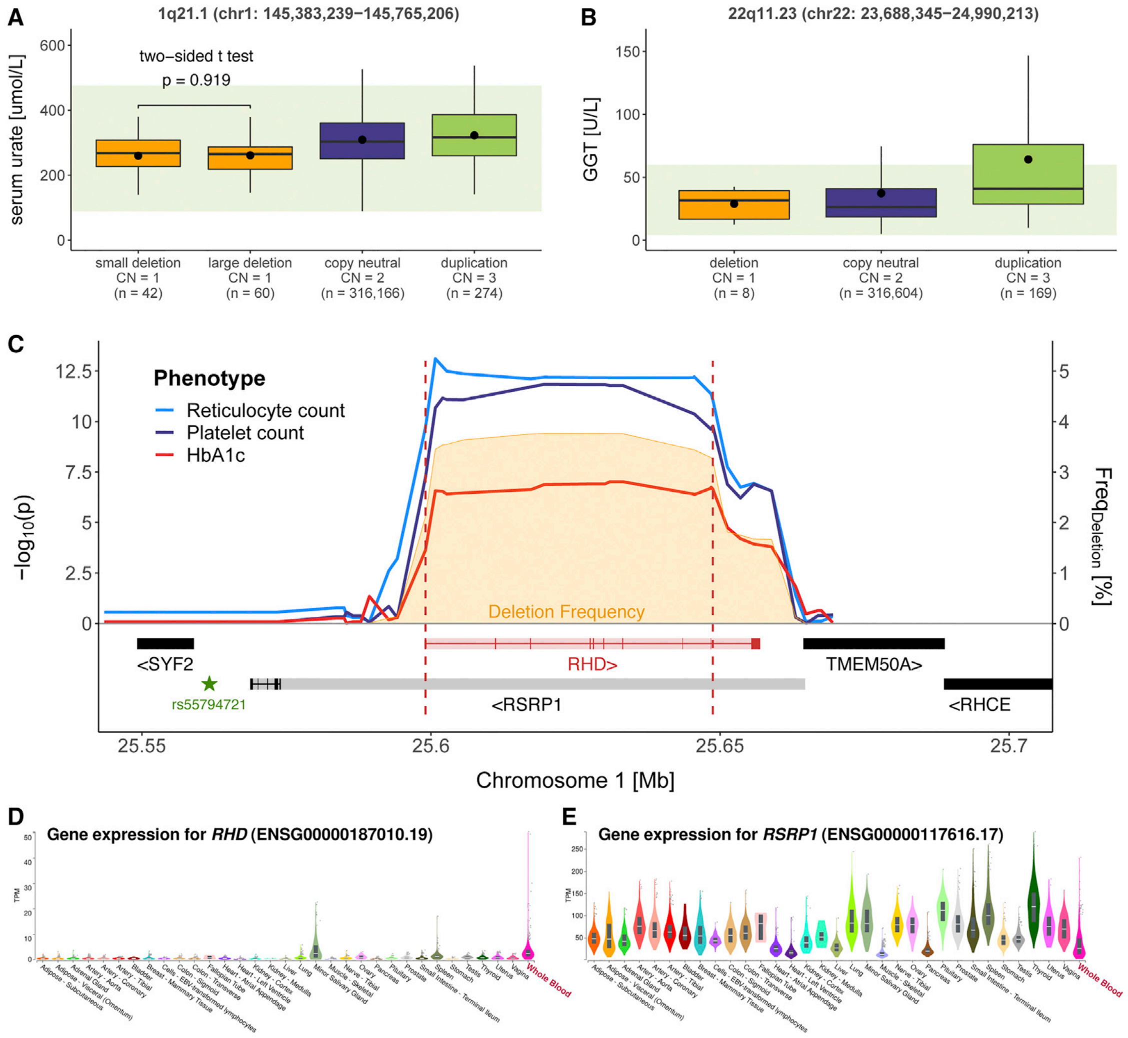

Figure 4. CNV-GWAS associations at SNP-GWAS loci

(A and B) Boxplots representing levels of (A) serum urate in individuals with a 1q21.1 (chr1: 145,383,239–145,765,206) overlapping small (start ≥ 145.6 Mb) or large (start < 145.6 Mb) deletion, copy-neutrality, or duplication and (B) γ-glutamyl transferase (GGT) in individuals with a 22q11.23 (chr22: 23,688,345–24,990,213) overlapping deletion, copy-neutrality, or duplication. Copy number (CN) and sample size (n) are reported for each category; boxes show the first (Q1), second (median, thick line), and third (Q3) quartiles; lower and upper whiskers show the most extreme value within Q1 minus and Q3 plus 1.5× the interquartile range, respectively; dots show the mean; outliers are not shown; light green backgrounds show normal clinical range for serum urate: 89–476 mmol/L (A) and GGT: 4–6 U/L (B). p value of a two-sided t test comparing serum urate levels of small and large 1q21.1 deletion carriers is shown.

(C) Association plot for the 1p36.11 deletion (chr1: 25,599,041–25,648,747). Red dashed lines delimit the deletion-only CNV region; left y axis shows the negative logarithm of association p value for reticulocyte count (blue), platelet count (purple), and glycated hemoglobin (HbA1c; red); right y axis shows deletion frequency [%] (orange); encompassed genes are schematically represented at the bottom; retained exons for the most strongly expressed isoform in whole blood are shown for *RHD* (ENST00000328664) and *RSRP1* (ENST00000243189), and shaded color represents the full gene sequence; star indicates the *RHD* and *RSRP1* expression quantitative locus rs55794721.

(D and E) GTEx v8 gene expression in 33 tissues for *RHD* (D) and *RSRP1* (E). Brain, cervix, esophagus, and skin are not shown for visibility. Whole blood is shown with a red label.

Strengthening the causal role of *RHD*'s CN, lack or strongly reduced expression of all Rh antigens, a rare condition named Rh deficiency or Rh_{null} syndrome [MIM: 617970 and 268150], is associated with increased erythrocyte osmotic fragility, resulting in hemolytic anemia.[83] Hemolytic anemia is characterized by increased reticulocyte count[84] and can falsely lower HbA1c levels because of decreased erythrocyte lifespan,[85] putting forward the hypothesis that heterozygous deletion of *RHD* leads to subclinical phenotypes akin to hemolytic anemia. To gauge the generalizability of these results, we looked for similar trends in individuals with Rh^{-} blood type, which can be caused by various polymorphisms.[82] Because Rhesus groups were unavailable for the UKBB, we turned to a maternity cohort from the Lausanne University Hospital. Despite low samples sizes, concordant trends of increased reticulocyte count ($\beta_{Rh-} = 1.07°/oo$; $p_{one\text{-}sided} = 0.134$; $n = 741$) and decreased platelet count ($\beta_{Rh-} = -2.8 \times 10^{9}$ cells/L; $p_{one\text{-}sided} = 0.126$;

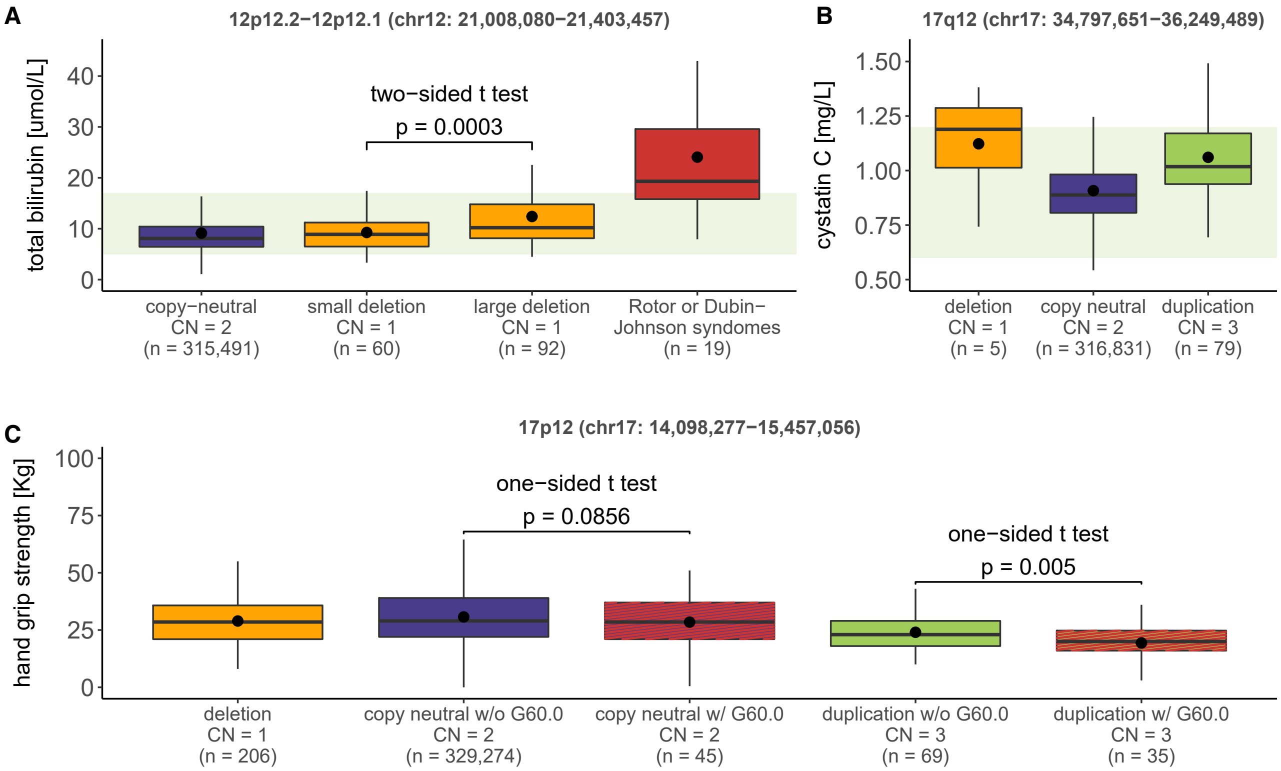

Figure 5. CNV-GWAS associations at Mendelian disorder loci
(A–C) Boxplots showing total bilirubin levels in copy-neutral individuals, small (start ≥ 21.1 Mb) or large (start < 21.1 Mb) 12p12.2-p12.1 (chr12: 21,008,080–21,403,457) overlapping deletion carriers, and Rotor or Dubin-Johnson syndrome-affected individuals (ICD-10 E80.6) (A), cystatin C levels in individuals with a 17q12 (chr17: 34,797,651–36,249,489) overlapping deletion, copy-neutrality, or duplication (B), and hand grip strength in individuals with a 17p12 (chr17: 14,098,277–15,457,056) overlapping deletion, copy-neutrality, or duplication, split according to the presence (w/) or absence (w/o) of a neuropathy (ICD-10 G60.0; red stripes) (C). Copy number (CN) and sample size (n) are reported for each category; boxes show the first (Q1), second (median, thick line), and third (Q3) quartiles; lower and upper whiskers show the most extreme value within Q1 minus and Q3 plus 1.5× the interquartile range, respectively; dots show the mean; outliers are not shown; light green backgrounds show normal clinical range for total bilirubin: 5–17 mmol/L (A) and cystatin C: 0.6–1.2 mg/L (B). p value of a two-sided t test comparing total bilirubin levels of small and large 12p12.2-p12.1 deletion carriers is shown. p values of one-sided t tests comparing hand grip strength of copy neutral and 17p12 duplication carriers with or without a neuropathy diagnosis are shown.

n = 5,034) and HbA1c levels ($\beta_{Rh-} = -0.22\%$; $p_{one\text{-}sided} = 0.050$; n = 418) were observed in Rh^- women (Table S5). Of note, reticulocyte and platelet counts have been reported to increase and decrease, respectively, along pregnancy,[86] and despite correcting for pregnancy status and gestational weeks, interaction between Rh^- blood group and pregnancy cannot be excluded. Impact of Rh blood type on hematological traits awaits validation but these examples illustrate how studying CNVs at SNP-GWAS loci can pinpoint causal genes and shared genetic mechanisms.

CNVs at Mendelian disorder loci

Despite the lower-than-average disease burden of UKBB participants,[87] several associations comprised loci involved in Mendelian disorders. The heterozygous 395 kb 12p12.2-p12.1 deletion, which associated with a non-pathological increase in total bilirubin (chr12: 21,008,080–21,403,457; $\beta_{del} = 3.1$ μmol/L, $p = 2.2 \times 10^{-13}$; Figure 5A) and harbors SNP-GWAS signals for bilirubin levels,[20,88–92] overlaps the Rotor syndrome locus (MIM: 237450), an extremely rare disorder whose main clinical manifestation is hyperbilirubinemia. Rotor syndrome[93] is caused by the homozygous disruption of *SLCO1B1* (MIM: 604843) and *SLCO1B3* (MIM: 605495) (Figure S7), which encode for the hepatic transporters OATP1B1 and OATP1B3, respectively, involved in the uptake of various drugs and metabolic compounds, including bilirubin.[94] Concordantly, UKBB participants diagnosed with Rotor syndrome or the related and more common Dubin-Johnson syndrome (MIM: 237500) presented above-normal levels of total bilirubin (Figure 5A). Interestingly, individuals carrying a partial deletion that only affects *SLCO1B1* (start ≥ 21.1 Mb; Figure S7) exhibited significantly milder increase in total bilirubin (two-sided t test: $p = 3.1 \times 10^{-4}$; Figure 5A), illustrating how mutations pathogenic in a digenic recessive framework can contribute to subtle changes in disease-associated phenotypes when present in an isolated heterozygous state.

A second example links the 1.5 Mb long 17q12 duplication (MIM: 614526) (chr17: 34,797,651–36,249,489) and

increased levels of kidney damage biomarkers, including cystatin C (β_{dup} = 0.15 mg/L, p = 4.2 × 10^{-17}; Figure 5B), serum creatinine (SCr; β_{dup} = 13.0 μmol/L, p = 2.7 × 10^{-16}; Figure S8A), and serum urea (β_{dup} = 0.93 mmol/L, p = 9.1 × 10^{-10}; Figure S8B), as well as the inflammation biomarker C-reactive protein (CRP; β_{mirror} = 2.3 mg/L, p = 1.1 × 10^{-6}; Figure S8C). Deletion of this interval (Figure S8D), as well as point mutations in overlapping *HNF1B* (MIM: 189907), cause the highly pathogenic and penetrant autosomal dominant renal cysts and diabetes syndrome (RCAD [MIM: 137920 and 614527]). RCAD is characterized by heterogenous structural and/or functional renal defects, neuro-developmental/psychiatric disorders, and maturity-onset diabetes of the young (see GeneReviews by Mitchel et al. in web resources). Because of the small number of deletion carriers (n = 6, regardless of phenotypic data availability), the deletion's effect was not assessed by CNV-GWASs, but elevated levels of cystatin C (Figure 5B), SCr (Figure S8A), and urea (Figure S8B) in these individuals align with RCAD's clinical description. Conversely, penetrance of the reciprocal duplication remains debated and only ~20% of diagnosed patients report renal abnormalities (see GeneReviews by Mefford in web resources). In line with a lower pathogenicity, we detected 16× more duplication than deletion carriers. Still, these individuals showed strong alterations in kidney biomarkers (Figure 5B; Figure S8), suggesting tight gene dosage control on *HNF1B*.

Third, we zoomed in on the 1.4 Mb long 17p12 duplication (Figure S9A) known as the main etiology of Charcot-Marie-Tooth (CMT) type 1A (MIM: 118220), a demyelinating neuropathy of the peripheral nervous system characterized by progressive muscle wasting.[95] Correspondingly, duplication carriers showed decreased hand grip strength (chr17: 14,098,277–15,457,056; β_{dup} = −9.8 kg, p = 4.1 × 10^{-39}; Figure 5C) and lower SCr (chr17: 14,098,277–15,468,444; β_{dup} = −13.8 μmol/L, p = 6.5 × 10^{-18}; Figure S9B; EstBB: β_{dup} = −94.1 μmol/L, p = 5.2 × 10^{-4}; Figure 3), indicating decreased muscle mass.[96] We next assessed the proportion of duplication carriers (regardless of phenotypic data availability) diagnosed with CMT or related hereditary motor and sensory neuropathies and detected 48 and 38 diagnoses among the 331,206 copy-neutral individuals and 107 duplication carriers, respectively. While there is a clear enrichment for CMT diagnoses among duplication carriers (Fisher's exact test: odds ratio = 3,668, p < 2.2 × 10^{-16}), only 36% of duplication carriers were clinically identified. To test whether these individuals presented with more extreme clinical manifestations, we compared grip strength and SCr levels in duplication carriers with or without a neuropathy diagnosis. The former group exhibited lower grip strength (one-sided t test: p = 0.005; Figure 5C) but no difference was detected in SCr levels (one-sided t test: p = 0.384; Figure S9B). Importantly, there was no age difference between diagnosed (mean = 55.5 years) and undiagnosed (mean = 56.2 years) duplication carriers (two-sided t test: p = 0.650), indicating that results do not reflect biases regarding age of disease onset.

These examples show that well-established pathogenic CNVs can modulate disease-associated phenotypes in the general population without necessarily causing clinically diagnosable disorders, supporting a model of variable expressivity for the involved loci.[97–100]

CNV-GWAS signals suggest gene functionalities

CNV-GWAS signals can corroborate or generate hypotheses regarding the function of encompassed genes, as shown with the association between the CN of a 1.2 Mb 16p13.11 interval and female reproductive traits. Specifically, duplication of the region correlated with decreased age at menarche (chr16: 15,120,501–16,308,285; β_{dup} = −0.6 years, p = 2.0 × 10^{-10}) and menopause (chr16: 15,151,451–16,308,285; β_{dup} = −1.8 years, p = 1.7 × 10^{-6}), whereas its deletion correlated with increased age at menarche (chr16: 15,120,501–16,308,285; β_{del} = 1.1 years, p = 3.6 × 10^{-7}), suggesting a shift in reproductive timing associated with the region's CN (Figures 6A and 6B) that aligns with a low, albeit positive, genetic correlation between the two traits (Neale Lab UKBB genetic correlation; web resources). Duplication effect on age at menarche (β_{dup} = −0.6 years, p = 1.8 × 10^{-2}) and menopause (β_{dup} = −2.6 years, p = 4.5 × 10^{-2}) were confirmed with nominal significance in the EstBB (Figure 3A) and a SNP-GWAS signal for age at menarche (rs153793) colocalized with the CNVR[101] (Figure 6C). Literature supports the role of *MARF1* (MIM: 614593) in this association. First, *MARF1* (observed/expected ratio [o/e] = 0.05 [0.03–0.12]; probability of loss-of-function intolerance [pLI] = 1) and *MYH11* (o/e = 0.22 [0.16–0.30]; pLI = 0.77; [MIM: 160745]) are the only encompassed genes under evolutionary constraint according to gnomAD[102] (upper bound of o/e <0.35; Figure 6C; Table S6; web resources). Second, *MARF1* was shown to play an essential role in murine oogenesis by fostering successful completion of meiosis and cytoplasmic maturation and protecting germline genomic integrity.[103] The gene's function is supported by studies in fly[104] and goat,[105] as well as two human case reports of females with *MARF1* mutations and reproduction phenotypes.[106,107] The female-specific role of *MARF1*[103–108] aligns with the absence of association with our proxies for male sexual maturation (i.e., age at first facial hair and balding). Although further investigations are warranted to characterize the function of *MARF1* in human female reproduction and assess the contribution of nearby genes and regulatory elements, it illustrates how CNV-GWASs can be leveraged to generate plausible hypotheses regarding gene functionalities.

The deleterious impact of a high CNV burden

Moving beyond single CNVs, the impact of an individual's total CNV burden on complex traits was estimated. Each participant's autosomal CNV, duplication, and deletion burden was calculated in number of affected Mb or genes. Both Mb

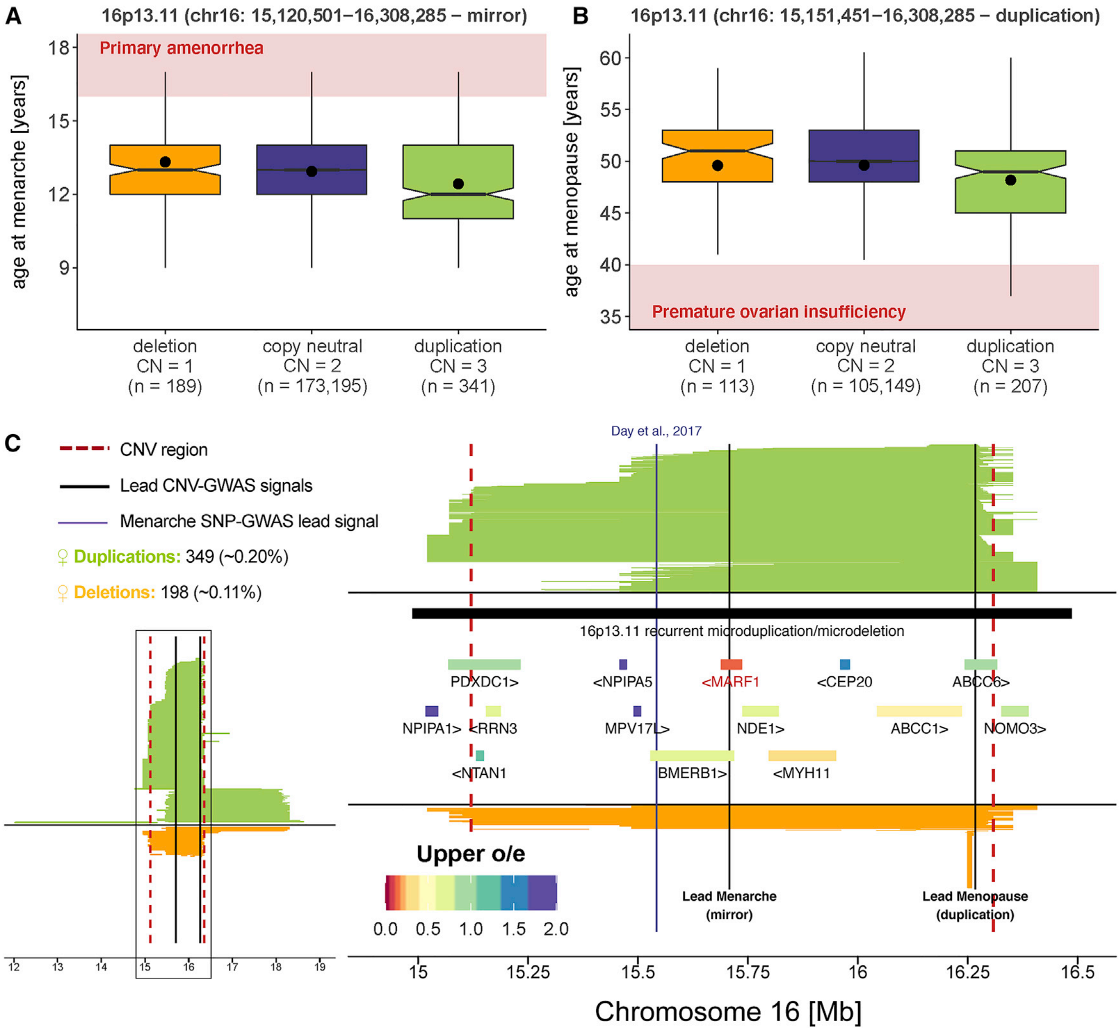

Figure 6. ***MARF1*** **as a putative gene involved in human female reproduction**

(A and B) Boxplots representing age at menarche (A) and menopause (B) in individuals with a 16p13.11 (A, chr16: 15,120,501–16,308,285; B, chr16: 15,151,451–16,308,285) overlapping deletion, copy-neutrality, or duplication. Copy number (CN) and sample size (n) are reported for each category; dots show the mean; boxes show the first (Q1), second (median, thick line), and third (Q3) quartiles; lower and upper whiskers show the most extreme value within Q1 minus and Q3 plus 1.5× the interquartile range, respectively; notches represent median $\pm$ 1.58 × IQR/$\sqrt{n}$; outliers are not shown; light red backgrounds indicate pathogenic values corresponding to primary amenorrhea (age at menarche > 16 years) (A)[109] and premature ovarian insufficiency (age at menopause < 40 years), respectively (B).[110]

(C) Mapping of CNVs overlapping the 16p13.11 CNV region (chr16: 15,120,501–16,308,285). Number and frequency of duplications and deletions are at the top left; left plot shows all overlapping CNVs; right plot focuses on the associated CNV region delineated with red dashed lines; duplications are in green, deletions in orange; black lines indicate the lead signal for age at menarche (mirror) and menopause (duplication-only); purple line indicates age at menarche-associated SNP;[101] overlapping recurrent DECIPHER CNV is shown in black and protein-coding genes are colored according to the upper bound of the confidence interval for the observed/expected (o/e) mutation ratio in gnomAD.

and gene burden metrices correlated well (ρ: 0.71–0.74) and while we observed high correlations (ρ: 0.40–0.92) between the CNV and duplication/deletion burdens, the two latter were uncorrelated (Figure 7A). From the 57 traits analyzed by CNV-GWASs, 35 (61%) significantly associated with at least one burden metric ($p \leq 0.05/63 = 7.9 \times 10^{-4}$, material and methods), showcasing negative health consequences such as increased levels of adiposity, liver/kidney damage biomarkers, leukocytes, glycemic values, or anxiety and decreased global physical capacity or intelligence (Figure 7B; Table S7). Harmful phenotypic consequences were often best captured by the number of deleted genes, in line with a higher sensitivity to decreased (i.e., haploinsufficiency) rather than increased (i.e., triplosensitivity) gene dosage.[111]

We then corrected each individual's phenotype and burden for the presence of trait-associated CNVs and performed the burden analysis anew to ensure that signals were not solely driven by significantly trait-associated CNVs (Figure 7C; Table S7). Whereas the association was lost for albumin, balding, body fat mass, GGT, triglycerides, and weight, indicating a mono- or oligogenic CNV

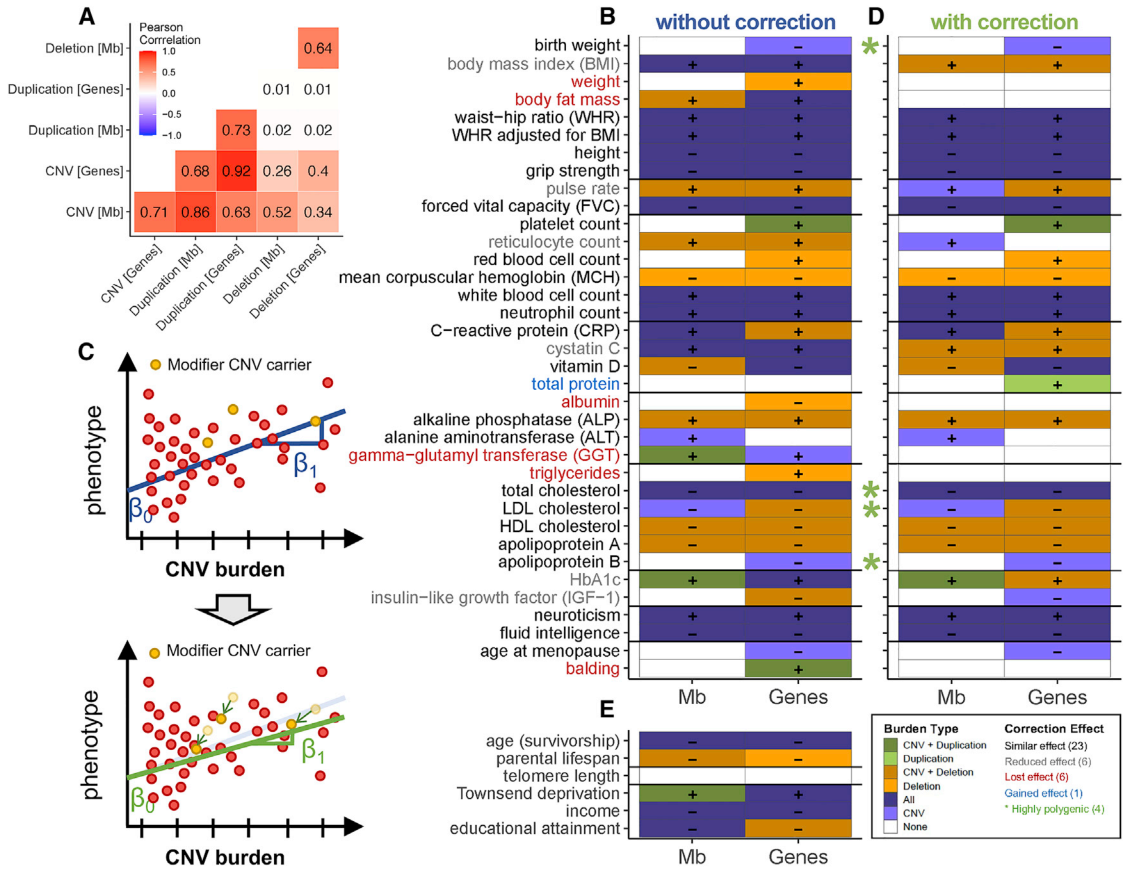

Figure 7. The negative impact of the CNV burden on complex traits

(A) Pearson correlation across six burden metrices.

(B) Significant associations ($p \leq 0.05/63 = 7.9 \times 10^{-4}$) between the CNV burden, expressed as the number of Mb or genes affected by CNVs (x axis), and traits assessed through CNV-GWASs (y axis). Color represents the type of burden—dark green, CNV and duplication-only; light green, duplication-only; dark orange, CNV and deletion-only; light orange, deletion-only; dark purple, CNV, duplication-only, and deletion-only; light purple, CNV; white, none—found to increase (+) or decrease (−) the considered phenotype.

(C) Schematic representation of the correction for modifier CNVs. Top: individuals carrying a CNV overlapping a CNV-GWAS region were identified (i.e., modifier CNV carrier; yellow). Bottom: Phenotype and burden were corrected (green arrows) and a new linear regression was fitted.

(D) Significant associations ($p \leq 0.05/63 = 7.9 \times 10^{-4}$) between the CNV burden after correction for modifier CNVs. Phenotype label color indicates whether the number of associated metrices between the CNV burden and the trait was fully lost (0 associations; red), decreased (gray), identical (black), or increased (blue) after the correction. Green stars mark highly polygenic traits associating with the CNV burden without having any significant CNV-GWAS signals.

(E) Significant associations ($p \leq 0.05/63 = 7.9 \times 10^{-4}$) between the CNV burden and life history traits (y axis). (D and E) follow the legend in (B).

architecture, 30 traits remained associated. Among these, birth weight, total cholesterol, low-density lipoprotein (LDL) cholesterol, and apolipoprotein B (ApoB) were significantly associated with the burden (Figure 7D) but lacked CNVR associations (Figure 2D). This indicates that, as established for SNPs,[1–3] the CNV architecture underlying most complex traits is polygenic, suggesting the presence of additional associations that we currently lack the power to detect.

The CNV burden extended its impact to global aspects of an individual's life, as illustrated by the negative correlation with several socio-economic factors, including decreased educational attainment (EA; $\beta_{burden} = -0.07$ years/Mb, $p = 4.4 \times 10^{-11}$) and income ($\beta_{burden} = -1{,}593$ £/year/Mb, $p = 2.9 \times 10^{-60}$), and increased Townsend deprivation index ($\beta_{burden} = 0.04$ SD/Mb, $p = 3.6 \times 10^{-7}$) (Figure 7E; Table S8). While we did not observe any effect of the CNV burden on age- and sex-corrected telomere length, the trait specifically associated with the *BRCA1* cancer locus[112] (MIM: 113705) (chr17: 41,197,733–41,258,551, $\beta_{dup} = 0.45$ SD, $p = 1.9 \times 10^{-8}$), paralleling findings that long telomere-associated SNPs also associate with increased cancer risk.[113] Because of the low number of deceased UKBB participants, we used proxies to assess the impact of the CNV burden on lifespan; we observed a negative association between an individual's CNV burden and both parental lifespan ($\beta_{burden} = -0.21$ years/Mb, $p = 1.4 \times 10^{-5}$) and age (survivorship proxy; $\beta_{burden} = -0.18$ years/Mb, $p = 1.1 \times 10^{-7}$), suggesting that the deleterious impact of CNVs contributes to decreased longevity (Figure 7E; Table S8). Given this, we

questioned whether the CNV burden was transmitted at a Mendelian rate. Taking advantage of the presence of a UKBB sibling for 16,179 individuals assessed in our previous analyses, we calculated that the average fraction of shared CNVs among siblings was 27%. Whereas substantially higher than for random pairs (0.7%), it only represents 54% of the expected fraction of shared additive genetic variance among siblings (50%).[114] Together, these results describe the broadly deleterious impact of CNVs on a wide range of complex traits in the general population and suggests that most traits are influenced by a polygenic CNV architecture.

Discussion

By coupling CNV calls to the phenotypic data available in the UKBB, we generated a roadmap of clinically relevant CNV-trait associations that allowed us to gain deeper insights into specific biological pathways and put forward general patterns describing the role of CNVs in shaping complex human traits in the general population.

Our UKBB CNV landscape matched previous reports,[18] and while some of the 131 CNV-GWAS signals overlapped known associations,[12,17,18,20] our analyses shed light on others that have not been studied extensively. Combined use of three association models revealed general patterns through which CNVs modulate phenotypes, and while geared toward the discovery of mirror effects, we also witnessed U-shape effects, illustrating different mechanisms through which altered dosage influences phenotypes. We further provide evidence for a broad and nuanced role of CNVs in shaping complex traits, as both common (frequency $\geq$ 1%) and rare (frequency $<$ 1%) CNVs mapping to regions involved by SNP-GWAS contribute to phenotypic variability in the general population, and rare CNVs have larger effects sizes than common ones. Other signals mapped to regions involved in Mendelian disorders. Studying pathogenic CNVs in the general population, as opposed to clinical cohorts selected on the basis of phenotypic criteria or family history, makes it possible to re-assess their frequency, penetrance, expressivity, pleiotropy, and inheritance pattern. Matching the increasing awareness around variable penetrance and expressivity,[97–100,115,116] we show that pathogenic dominant CNVs can impact disease-associated traits without causing clinically diagnosable disorders, whereas recessive CNVs can impact disease-related biomarkers at the heterozygous state. Together, these results provide a more complex and nuanced—but also broader—understanding of the phenotypic impact of CNVs at odds with the classical dichotomy between common complex diseases and rare Mendelian disorders.

Confirming the deleterious influence of a high CNV load on anthropometric traits[17,117,118] and EA[11,119,120] in a non-clinical cohort, we extended this observation to over 30 global health biomarkers. We show how the CNV burden—limited to large and rare CNVs detectable by microarrays—shapes intermediate molecular phenotypes that predate or are consequences of disease processes in a population-based cohort, consistent with its known contribution to a wide range of disorders.[121–125] Our data further show that the CNV load negatively impacts socio-economic factors and longevity proxies. The lower CNV burden observed in individuals with advanced age matches the depletion of life-shortening alleles in older UKBB participants,[126] suggesting improved health/decreased mortality in individuals with a low CNV load. Parental lifespan negatively correlated with the CNV burden. While lower than expected, a substantial fraction of CNVs (27%) was shared among siblings and thus inherited from either parent. As inclusion of haplotype sharing information in CNV calling mainly increases the detection of small ($<$10 kb) but not that of large CNVs,[21] we hypothesize that large events recurrently appear *de novo* on multiple backgrounds and are rapidly eliminated from the population through transmission bias or from the cohort through ascertainment bias (i.e., increased participation of healthier siblings) because of their deleteriousness. Our analysis of CNV call quality in the EstBB suggests marginal contribution of false CNV calls but confounders–such as CNV length, which affects both detection capacity and pathogenicity–prevent the assessment of these factors separately. Nevertheless, the lower-than-expected CNV inheritance allow speculating that an even stronger association with lifespan would be obtained providing access to parental CNV genotypes. If further studies are required to confirm the life-shortening effect of a high CNV load, our data clearly illustrate the deleterious impact of CNVs on an individual's global health.

Both CNV-GWASs and burden analyses results improve the understanding of the CNV architecture underlying studied traits. Many CNV-GWAS loci involve rare but recurrent CNVRs. Due to the difficulty of gathering large cohorts of carriers, complete phenotypic characterization of these loci is still missing and limited to easily assessed anthropometric traits or severely debilitating neuro-developmental/psychiatric disorders. Our results provide a map of the pleiotropic consequences of these CNVRs on over 50 medically relevant traits. Some traits are not typically assessed/reported in patient cohorts and targeted study of their distribution among cases might refine diagnostic criteria and help clinicians identify and follow-up on patients with mild and/or atypical presentation. Mechanistically, most assessed CNVRs are large, potentially harboring several causal genes. One of the next challenges will be to narrow down causal region(s) in pleiotropic multi-genic CNVRs to untangle primary from secondary associated traits, as some, such as obesity, are known to causally alter multiple biomarkers.[127–129] The substantial overlap between CNV- and SNP-GWAS signals speaks for the presence of shared genetic mechanisms, so that both mutational classes can be exploited synergistically to pinpoint causal genes and elucidate their biological function. In parallel, we observed a high degree of CNV-polygenicity, as 30 out

of 35 traits remained associated with the CNV burden after correction for modifier CNVRs. For six traits, CNV-GWAS signals captured the bulk of phenotypic variability caused by CNVs, while ApoB, birth weight, LDL cholesterol, and total cholesterol were solely associated with the CNV burden. This indicates a polygenic CNV architecture that might arise from rare high impact CNVs that were not assessed by CNV-GWASs (frequency $\leq$ 0.005%) and/or more frequent CNVs with mild effects; indeed, most high frequency CNVRs do not overlap CNV-GWAS signals (Figures 1 and 2D). Among these, decreased birth weight, which associated with a high CNV load, has been linked to increased risk for metabolic syndrome, obesity, and various other diseases in adulthood,[130,131] opening the question as to whether some of the deleterious effects of the CNV burden are rooted in early development. Strikingly, the three other traits are plasma lipids with few CNV-GWAS signals. Speaking for their high polygenicity, a GWAS on 35 blood biomarkers in the UKBB found an average of 87 versus 478 associations per trait for non-lipid compared to lipid traits.[20] Collectively, these results illustrate a more complex than expected contribution of CNVs in shaping the genetic architecture of complex human traits.

It is important to keep in mind limitations of the current study. First, CNVs were called on the basis of microarray data with PennCNV. In addition to high false positive rates associated to array-based CNV calls, this renders the study blind to variants in regions not covered by the array, limits resolution—both in length and exact break point location—and hinders the detection of high copy-number states (CN $\geq$ 4) and deviations thereof. To mitigate these issues, we stringently filtered CNVs and transformed calls to the probe level,[17,25] which at risk of missing true associations guarantees the identification of trustworthy CNV-trait pairs. Few cohorts have sufficiently large genetic and phenotypic coverage to replicate UKBB findings at adequate power, so that we relied on literature evidence to gauge the validity of our results, highlighting the need for large-sized biobanks for studying (rare) CNVs. Future release of large sequencing datasets combined to progress in CNV detection tools could resolve these issues and lead to novel discoveries.[21,80,132,133] Second, despite substantial evidence of CNV- and SNP-GWAS signal colocalization, we did not perform robust enrichment analyses, as the non-random genomic distribution and complex nature of CNVs renders simulating the null scenario beyond the scope of this paper. Signal colocalization is likely to be underestimated, as manual literature searches revealed overlaps missed by our annotation pipeline (e.g., 16p13.11 age at menarche signal[101]) and we obtained a 7% increase in signal colocalization by using GWAS Catalog annotation 6 months apart (31% April 2021 → 38% October 2021). Third, our study is limited to individuals of White British ancestry. As CNV frequencies vary across populations,[5,134–136] assessing diverse ancestral groups is likely to unravel new associations, even though smaller sample sizes represent a limiting factor. Finally, the UKBB suffers from a "healthy cohort" bias.[87] Focusing on the impact of CNVs in healthy populations, we used this bias to our advantage through the inclusion of CNV carriers with sub-clinical phenotypes, providing lower bounds for effect size estimates.[99,100,137] However, this means that the cohort is depleted for severely affected cases and extremely rare (frequency $\leq$ 0.005%) but highly pathogenic CNVs were not tested for associations. Extending the analysis to low frequency/high impact CNVRs would allow for better distinguishing of mechanisms of action—with the remaining caveat that effects will be underestimated because of selection bias—and will be the focus of future work.

In conclusion, our study provides a map of high-confidence CNV-trait associations. While we explored some of the reported signals, collective efforts will be required to validate and interpret these discoveries and we hope that this resource will be useful for researchers and clinicians aiming at improving the characterization of recurrent CNVs. Our study revealed the nuanced role of CNVs along the rare versus common disease spectrum, their shared mechanisms with SNPs, as well as a widespread polygenic CNV architecture, consolidating the growing body of evidence implicating CNVs in the shaping of complex human traits.

Data and code availability

Code is available on GitHub at https://github.com/cauwerx/CNV_GWAS_continuous_traits. Probe-level CNV frequencies (UKBB and EstBB) and CNV-GWAS summary statistics (UKBB) are deposited on Mendeley Data at https://dx.doi.org/10.17632/z54dc3b6jz.1. The accession numbers for UKBB CNV-GWASs summary statistics reported in this paper are GWAS Catalog: GCST90027274–GCST90027444.

Supplemental information

Supplemental information can be found online at https://doi.org/10.1016/j.ajhg.2022.02.010.

Consortia

The members of the Estonian Biobank Research Team are Tõnu Esko, Andres Metspalu, Lili Milani, Reedik Mägi, and Mari Nelis.

Acknowledgments

This research has been conducted with the UK Biobank Resource under application number 16389; we thank all biobank participants for sharing their data. UKBB and EstBB computations were carried out on the JURA server (University of Lausanne) and the High-Performance Computing Center (University of Tartu), respectively. This work was supported by funding from the Department of Computational Biology (Z.K.) and the Center for Integrative Genomics (A.R.) from the University of Lausanne, as well as grants from the Swiss National Science Foundation (31003A_182632, A.R.), Horizon2020 Twinning projects (ePerMed 692145, A.R.), and the Estonian Research Council (PRG687, M.L.

and R.M.). Critical reading of the draft by Johan Auwerx and Matthew Robinson was appreciated.

Declaration of interests

The authors declare no competing interests.

Received: October 5, 2021
Accepted: February 9, 2022
Published: March 2, 2022

Web resources

DECIPHER CNV Syndromes list, https://www.deciphergenomics.org/disorders/syndromes/list

GeneReviews, Mefford, H. (2021). 17q12 Recurrent Duplication, https://www.ncbi.nlm.nih.gov/books/NBK344340/

GeneReviews, Mitchel, M.W., Moreno-De-Luca, D., Myers, S.M., Levy, R.V., Turner, S., Ledbetter, D.H., and Martin, C.L. (2020). 17q12 Recurrent Deletion Syndrome, https://www.ncbi.nlm.nih.gov/books/NBK401562/

Genome Reference Consortium GRCh37, https://www.ncbi.nlm.nih.gov/grc/human/data

gnomAD (v2.1.1), https://gnomad.broadinstitute.org/

GTEx project (v8) portal, https://gtexportal.org/home/

Neale Lab UKBB genetic correlation, https://ukbb-rg.hail.is/

NHGRI-EBI GWAS Catalog, https://www.ebi.ac.uk/gwas/

OMIM, https://www.omim.org/

ScyMed MediCalc, http://www.scymed.com/en/smnxfd/smnxfdad.htm

References

1. Visscher, P.M., Wray, N.R., Zhang, Q., Sklar, P., McCarthy, M.I., Brown, M.A., and Yang, J. (2017). 10 Years of GWAS Discovery: Biology, Function, and Translation. Am. J. Hum. Genet. *101*, 5–22.
2. Watanabe, K., Stringer, S., Frei, O., Umićević Mirkov, M., de Leeuw, C., Polderman, T.J.C., van der Sluis, S., Andreassen, O.A., Neale, B.M., and Posthuma, D. (2019). A global overview of pleiotropy and genetic architecture in complex traits. Nat. Genet. *51*, 1339–1348.
3. Canela-Xandri, O., Rawlik, K., and Tenesa, A. (2018). An atlas of genetic associations in UK Biobank. Nat. Genet. *50*, 1593–1599.
4. Manolio, T.A., Collins, F.S., Cox, N.J., Goldstein, D.B., Hindorff, L.A., Hunter, D.J., McCarthy, M.I., Ramos, E.M., Cardon, L.R., Chakravarti, A., et al. (2009). Finding the missing heritability of complex diseases. Nature *461*, 747–753.
5. Sudmant, P.H., Rausch, T., Gardner, E.J., Handsaker, R.E., Abyzov, A., Huddleston, J., Zhang, Y., Ye, K., Jun, G., Fritz, M.H.Y., et al. (2015). An integrated map of structural variation in 2,504 human genomes. Nature *526*, 75–81.
6. Shaikh, T.H. (2017). Copy Number Variation Disorders. Curr. Genet. Med. Rep. *5*, 183–190.
7. Firth, H.V., Richards, S.M., Bevan, A.P., Clayton, S., Corpas, M., Rajan, D., Van Vooren, S., Moreau, Y., Pettett, R.M., and Carter, N.P. (2009). DECIPHER: Database of Chromosomal Imbalance and Phenotype in Humans Using Ensembl Resources. Am. J. Hum. Genet. *84*, 524–533.
8. Kathiresan, S., Voight, B.F., Purcell, S., Musunuru, K., Ardissino, D., Mannucci, P.M., Anand, S., Engert, J.C., Samani, N.J., Schunkert, H., et al. (2009). Genome-wide association of early-onset myocardial infarction with single nucleotide polymorphisms and copy number variants. Nat. Genet. *41*, 334–341.
9. Craddock, N., Hurles, M.E., Cardin, N., Pearson, R.D., Plagnol, V., Robson, S., Vukcevic, D., Barnes, C., Conrad, D.F., Giannoulatou, E., et al. (2010). Genome-wide association study of CNVs in 16,000 cases of eight common diseases and 3,000 shared controls. Nature *464*, 713–720.
10. Valsesia, A., Macé, A., Jacquemont, S., Beckmann, J.S., and Kutalik, Z. (2013). The growing importance of CNVs: New insights for detection and clinical interpretation. Front. Genet. *4*, 92.
11. Kendall, K.M., Rees, E., Escott-Price, V., Einon, M., Thomas, R., Hewitt, J., O'Donovan, M.C., Owen, M.J., Walters, J.T.R., and Kirov, G. (2017). Cognitive Performance Among Carriers of Pathogenic Copy Number Variants: Analysis of 152,000 UK Biobank Subjects. Biol. Psychiatry *82*, 103–110.
12. Owen, D., Bracher-Smith, M., Kendall, K.M., Rees, E., Einon, M., Escott-Price, V., Owen, M.J., O'Donovan, M.C., and Kirov, G. (2018). Effects of pathogenic CNVs on physical traits in participants of the UK Biobank 11 Medical and Health Sciences 1103 Clinical Sciences. BMC Genomics *19*, 1–9.
13. Warland, A., Kendall, K.M., Rees, E., Kirov, G., and Caseras, X. (2020). Schizophrenia-associated genomic copy number variants and subcortical brain volumes in the UK Biobank. Mol. Psychiatry *25*, 854–862.
14. Crawford, K., Bracher-Smith, M., Owen, D., Kendall, K.M., Rees, E., Pardiñas, A.F., Einon, M., Escott-Price, V., Walters, J.T.R., O'Donovan, M.C., et al. (2019). Medical consequences of pathogenic CNVs in adults: analysis of the UK Biobank. J. Med. Genet. *56*, 131–138.
15. Kendall, K.M., Rees, E., Bracher-Smith, M., Legge, S., Riglin, L., Zammit, S., O'Donovan, M.C., Owen, M.J., Jones, I., Kirov, G., and Walters, J.T.R. (2019). Association of Rare Copy Number Variants With Risk of Depression. JAMA Psychiatry *76*, 818–825.
16. Bracher-Smith, M., Kendall, K., Rees, E., Einon, M., O'Donovan, M., Owen, M., and Kirov, G. (2019). Effects of pathogenic CNVs on biochemical markers: a study on the UK Biobank. Preprint at bioRxiv. https://doi.org/10.1101/723270.
17. Macé, A., Tuke, M.A., Deelen, P., Kristiansson, K., Mattsson, H., Nõukas, M., Sapkota, Y., Schick, U., Porcu, E., Rüeger, S., et al. (2017). CNV-association meta-analysis in 191,161 European adults reveals new loci associated with anthropometric traits. Nat. Commun. *8*, 744.
18. Aguirre, M., Rivas, M.A., and Priest, J. (2019). Phenome-wide Burden of Copy-Number Variation in the UK Biobank. Am. J. Hum. Genet. *105*, 373–383.
19. Li, Y.R., Glessner, J.T., Coe, B.P., Li, J., Mohebnasab, M., Chang, X., Connolly, J., Kao, C., Wei, Z., Bradfield, J., et al. (2020). Rare copy number variants in over 100,000 European ancestry subjects reveal multiple disease associations. Nat. Commun. *11*, 255.
20. Sinnott-Armstrong, N., Tanigawa, Y., Amar, D., Mars, N., Benner, C., Aguirre, M., Venkataraman, G.R., Wainberg, M., Ollila, H.M., Kiiskinen, T., et al. (2021). Genetics of 35 blood and urine biomarkers in the UK Biobank. Nat. Genet. *53*, 185–194.

21. Hujoel, M.L.A., Sherman, M.A., Barton, A.R., Mukamel, R.E., Vijay, S.G., and Loh, P.-R. (2021). Influences of rare copy number variation on human complex traits. Preprint at bioRxiv. https://doi.org/10.1101/2021.10.21.465308.
22. Bycroft, C., Freeman, C., Petkova, D., Band, G., Elliott, L.T., Sharp, K., Motyer, A., Vukcevic, D., Delaneau, O., O'Connell, J., et al. (2018). The UK Biobank resource with deep phenotyping and genomic data. Nature *562*, 203–209.
23. Leitsalu, L., Haller, T., Esko, T., Tammesoo, M.L., Alavere, H., Snieder, H., Perola, M., Ng, P.C., Mägi, R., Milani, L., et al. (2015). Cohort profile: Estonian biobank of the Estonian genome center, university of Tartu. Int. J. Epidemiol. *44*, 1137–1147.
24. Wang, K., Li, M., Hadley, D., Liu, R., Glessner, J., Grant, S.F.A., Hakonarson, H., and Bucan, M. (2007). PennCNV: an integrated hidden Markov model designed for high-resolution copy number variation detection in whole-genome SNP genotyping data. Genome Res. *17*, 1665–1674.
25. Macé, A., Tuke, M.A., Beckmann, J.S., Lin, L., Jacquemont, S., Weedon, M.N., Reymond, A., and Kutalik, Z. (2016). New quality measure for SNP array based CNV detection. Bioinformatics *32*, 3298–3305.
26. Chang, C.C., Chow, C.C., Tellier, L.C.A.M., Vattikuti, S., Purcell, S.M., and Lee, J.J. (2015). Second-generation PLINK: rising to the challenge of larger and richer datasets. Gigascience *4*, 7.
27. Wang, K., Li, M., and Hakonarson, H. (2010). ANNOVAR: functional annotation of genetic variants from high-throughput sequencing data. Nucleic Acids Res. *38*, e164.
28. Mägi, R., and Morris, A.P. (2010). GWAMA: software for genome-wide association meta-analysis. BMC Bioinformatics *11*, 288.
29. Wu, P., Gifford, A., Meng, X., Li, X., Campbell, H., Varley, T., Zhao, J., Carroll, R., Bastarache, L., Denny, J.C., et al. (2019). Mapping ICD-10 and ICD-10-CM Codes to Phecodes: Workflow Development and Initial Evaluation. JMIR Med. Inform. *7*, e14325.
30. Gao, X., Starmer, J., and Martin, E.R. (2008). A multiple testing correction method for genetic association studies using correlated single nucleotide polymorphisms. Genet. Epidemiol. *32*, 361–369.
31. Buniello, A., MacArthur, J.A.L., Cerezo, M., Harris, L.W., Hayhurst, J., Malangone, C., McMahon, A., Morales, J., Mountjoy, E., Sollis, E., et al. (2019). The NHGRI-EBI GWAS Catalog of published genome-wide association studies, targeted arrays and summary statistics 2019. Nucleic Acids Res. *47* (D1), D1005–D1012.
32. Handsaker, R.E., Van Doren, V., Berman, J.R., Genovese, G., Kashin, S., Boettger, L.M., and McCarroll, S.A. (2015). Large multiallelic copy number variations in humans. Nat. Genet. *47*, 296–303.
33. Porcu, E., Rüeger, S., Lepik, K., Santoni, F.A., Reymond, A., Kutalik, Z.; eQTLGen Consortium; and BIOS Consortium (2019). Mendelian randomization integrating GWAS and eQTL data reveals genetic determinants of complex and clinical traits. Nat. Commun. *10*, 3300.
34. Gilman, S.R., Iossifov, I., Levy, D., Ronemus, M., Wigler, M., and Vitkup, D. (2011). Rare de novo variants associated with autism implicate a large functional network of genes involved in formation and function of synapses. Neuron *70*, 898–907.
35. Levy, D., Ronemus, M., Yamrom, B., Lee, Y.H., Leotta, A., Kendall, J., Marks, S., Lakshmi, B., Pai, D., Ye, K., et al. (2011). Rare de novo and transmitted copy-number variation in autistic spectrum disorders. Neuron *70*, 886–897.
36. Jacquemont, S., Coe, B.P., Hersch, M., Duyzend, M.H., Krumm, N., Bergmann, S., Beckmann, J.S., Rosenfeld, J.A., and Eichler, E.E. (2014). A higher mutational burden in females supports a "female protective model" in neurodevelopmental disorders. Am. J. Hum. Genet. *94*, 415–425.
37. Sanders, S.J., He, X., Willsey, A.J., Ercan-Sencicek, A.G., Samocha, K.E., Cicek, A.E., Murtha, M.T., Bal, V.H., Bishop, S.L., Dong, S., et al. (2015). Insights into Autism Spectrum Disorder Genomic Architecture and Biology from 71 Risk Loci. Neuron *87*, 1215–1233.
38. Collins, R.L., Brand, H., Karczewski, K.J., Zhao, X., Alföldi, J., Francioli, L.C., Khera, A.V., Lowther, C., Gauthier, L.D., Wang, H., et al. (2020). A structural variation reference for medical and population genetics. Nature *581*, 444–451.
39. Abel, H.J., Larson, D.E., Regier, A.A., Chiang, C., Das, I., Kanchi, K.L., Layer, R.M., Neale, B.M., Salerno, W.J., Reeves, C., et al. (2020). Mapping and characterization of structural variation in 17,795 human genomes. Nature *583*, 83–89.
40. Rao, E., Weiss, B., Fukami, M., Rump, A., Niesler, B., Mertz, A., Muroya, K., Binder, G., Kirsch, S., Winkelmann, M., et al. (1997). Pseudoautosomal deletions encompassing a novel homeobox gene cause growth failure in idiopathic short stature and Turner syndrome. Nat. Genet. *16*, 54–63.
41. Ellison, J.W., Wardak, Z., Young, M.F., Gehron Robey, P., Laig-Webster, M., and Chiong, W. (1997). PHOG, a candidate gene for involvement in the short stature of Turner syndrome. Hum. Mol. Genet. *6*, 1341–1347.
42. Fukami, M., Seki, A., and Ogata, T. (2016). SHOX Haploinsufficiency as a Cause of Syndromic and Nonsyndromic Short Stature. Mol. Syndromol. *7*, 3–11.
43. Schiller, S., Spranger, S., Schechinger, B., Fukami, M., Merker, S., Drop, S.L.S., Tröger, J., Knoblauch, H., Kunze, J., Seidel, J., and Rappold, G.A. (2000). Phenotypic variation and genetic heterogeneity in Léri-Weill syndrome. Eur. J. Hum. Genet. *8*, 54–62.
44. Frank, Gaillard, and Mostafa, El-Feky (2021). Madelung deformity. https://radiopaedia.org/articles/7582.
45. Mefford, H.C., Sharp, A.J., Baker, C., Itsara, A., Jiang, Z., Buysse, K., Huang, S., Maloney, V.K., Crolla, J.A., Baralle, D., et al. (2008). Recurrent rearrangements of chromosome 1q21.1 and variable pediatric phenotypes. N. Engl. J. Med. *359*, 1685–1699.
46. Bernier, R., Steinman, K.J., Reilly, B., Wallace, A.S., Sherr, E.H., Pojman, N., Mefford, H.C., Gerdts, J., Earl, R., Hanson, E., et al. (2016). Clinical phenotype of the recurrent 1q21.1 copy-number variant. Genet. Med. *18*, 341–349.
47. Brunetti-Pierri, N., Berg, J.S., Scaglia, F., Belmont, J., Bacino, C.A., Sahoo, T., Lalani, S.R., Graham, B., Lee, B., Shinawi, M., et al. (2008). Recurrent reciprocal 1q21.1 deletions and duplications associated with microcephaly or macrocephaly and developmental and behavioral abnormalities. Nat. Genet. *40*, 1466–1471.
48. Conrad, D.F., Pinto, D., Redon, R., Feuk, L., Gokcumen, O., Zhang, Y., Aerts, J., Andrews, T.D., Barnes, C., Campbell, P., et al. (2010). Origins and functional impact of copy number variation in the human genome. Nature *464*, 704–712.
49. Bochukova, E.G., Huang, N., Keogh, J., Henning, E., Purmann, C., Blaszczyk, K., Saeed, S., Hamilton-Shield, J.,

Clayton-Smith, J., O'Rahilly, S., et al. (2010). Large, rare chromosomal deletions associated with severe early-onset obesity. Nature *463*, 666–670.

50. Jacquemont, S., Reymond, A., Zufferey, F., Harewood, L., Walters, R.G., Kutalik, Z., Martinet, D., Shen, Y., Valsesia, A., Beckmann, N.D., et al. (2011). Mirror extreme BMI phenotypes associated with gene dosage at the chromosome 16p11.2 locus. Nature *478*, 97–102.
51. Bachmann-Gagescu, R., Mefford, H.C., Cowan, C., Glew, G.M., Hing, A.V., Wallace, S., Bader, P.I., Hamati, A., Reitnauer, P.J., Smith, R., et al. (2010). Recurrent 200-kb deletions of 16p11.2 that include the SH2B1 gene are associated with developmental delay and obesity. Genet. Med. *12*, 641–647.
52. Loviglio, M.N., Leleu, M., Männik, K., Passeggeri, M., Giannuzzi, G., van der Werf, I., Waszak, S.M., Zazhytska, M., Roberts-Caldeira, I., Gheldof, N., et al. (2017). Chromosomal contacts connect loci associated with autism, BMI and head circumference phenotypes. Mol. Psychiatry *22*, 836–849.
53. Männik, K., Arbogast, T., Lepamets, M., Lepik, K., Pellaz, A., Ademi, H., Kupchinsky, Z.A., Ellegood, J., Attanasio, C., Messina, A., et al. (2019). Leveraging biobank-scale rare and common variant analyses to identify. Preprint at bioRxiv. https://doi.org/10.1101/716415.
54. Kargi, A.Y., and Merriam, G.R. (2013). Diagnosis and treatment of growth hormone deficiency in adults. Nat. Rev. Endocrinol. *9*, 335–345.
55. Andrews, N.C. (2009). Genes determining blood cell traits. Nat. Genet. *41*, 1161–1162.
56. Ganesh, S.K., Zakai, N.A., van Rooij, F.J.A., Soranzo, N., Smith, A.V., Nalls, M.A., Chen, M.H., Kottgen, A., Glazer, N.L., Dehghan, A., et al. (2009). Multiple loci influence erythrocyte phenotypes in the CHARGE Consortium. Nat. Genet. *41*, 1191–1198.
57. Aguirre, G.A., De Ita, J.R., de la Garza, R.G., and Castilla-Cortazar, I. (2016). Insulin-like growth factor-1 deficiency and metabolic syndrome. J. Transl. Med. *14*, 3.
58. Sakaue, S., Kanai, M., Tanigawa, Y., Karjalainen, J., Kurki, M., Koshiba, S., Narita, A., Konuma, T., Yamamoto, K., Akiyama, M., et al. (2021). A cross-population atlas of genetic associations for 220 human phenotypes. Nat. Genet. *53*, 1415–1424.
59. Rudd, M.K., Keene, J., Bunke, B., Kaminsky, E.B., Adam, M.P., Mulle, J.G., Ledbetter, D.H., and Martin, C.L. (2009). Segmental duplications mediate novel, clinically relevant chromosome rearrangements. Hum. Mol. Genet. *18*, 2957–2962.
60. Cooper, G.M., Coe, B.P., Girirajan, S., Rosenfeld, J.A., Vu, T.H., Baker, C., Williams, C., Stalker, H., Hamid, R., Hannig, V., et al. (2011). A copy number variation morbidity map of developmental delay. Nat. Genet. *43*, 838–846.
61. Yu, H.E., Hawash, K., Picker, J., Stoler, J., Urion, D., Wu, B.L., and Shen, Y. (2012). A recurrent 1.71 Mb genomic imbalance at 2q13 increases the risk of developmental delay and dysmorphism. Clin. Genet. *81*, 257–264.
62. Riley, K.N., Catalano, L.M., Bernat, J.A., Adams, S.D., Martin, D.M., Lalani, S.R., Patel, A., Burnside, R.D., Innis, J.W., and Rudd, M.K. (2015). Recurrent deletions and duplications of chromosome 2q11.2 and 2q13 are associated with variable outcomes. Am. J. Med. Genet. A. *167A*, 2664–2673.
63. Wolfe, K., McQuillin, A., Alesi, V., Boudry Labis, E., Cutajar, P., Dallapiccola, B., Dentici, M.L., Dieux-Coeslier, A., Duban-Bedu, B., Duelund Hjortshøj, T., et al. (2018). Delineating the psychiatric and behavioral phenotype of recurrent 2q13 deletions and duplications. Am. J. Med. Genet. B. Neuropsychiatr. Genet. *177*, 397–405.
64. De Bruyne, E., Bos, T.J., Schuit, F., Van Valckenborgh, E., Menu, E., Thorrez, L., Atadja, P., Jernberg-Wiklund, H., and Vanderkerken, K. (2010). IGF-1 suppresses Bim expression in multiple myeloma via epigenetic and posttranslational mechanisms. Blood *115*, 2430–2440.
65. Anzai, N., Miyazaki, H., Noshiro, R., Khamdang, S., Chairoungdua, A., Shin, H.-J., Enomoto, A., Sakamoto, S., Hirata, T., Tomita, K., et al. (2004). The multivalent PDZ domain-containing protein PDZK1 regulates transport activity of renal urate-anion exchanger URAT1 via its C terminus. J. Biol. Chem. *279*, 45942–45950.
66. Kolz, M., Johnson, T., Sanna, S., Teumer, A., Vitart, V., Perola, M., Mangino, M., Albrecht, E., Wallace, C., Farrall, M., et al. (2009). Meta-analysis of 28,141 individuals identifies common variants within five new loci that influence uric acid concentrations. PLoS Genet. *5*, e1000504.
67. Köttgen, A., Albrecht, E., Teumer, A., Vitart, V., Krumsiek, J., Hundertmark, C., Pistis, G., Ruggiero, D., O'Seaghdha, C.M., Haller, T., et al. (2013). Genome-wide association analyses identify 18 new loci associated with serum urate concentrations. Nat. Genet. *45*, 145–154.
68. Yang, Q., Köttgen, A., Dehghan, A., Smith, A.V., Glazer, N.L., Chen, M.H., Chasman, D.I., Aspelund, T., Eiriksdottir, G., Harris, T.B., et al. (2010). Multiple genetic loci influence serum urate levels and their relationship with gout and cardiovascular disease risk factors. Circ Cardiovasc Genet *3*, 523–530.
69. Sulem, P., Gudbjartsson, D.F., Walters, G.B., Helgadottir, H.T., Helgason, A., Gudjonsson, S.A., Zanon, C., Besenbacher, S., Bjornsdottir, G., Magnusson, O.T., et al. (2011). Identification of low-frequency variants associated with gout and serum uric acid levels. Nat. Genet. *43*, 1127–1130.
70. Ketharnathan, S., Leask, M., Boocock, J., Phipps-Green, A.J., Antony, J., O'Sullivan, J.M., Merriman, T.R., and Horsfield, J.A. (2018). A non-coding genetic variant maximally associated with serum urate levels is functionally linked to HNF4A-dependent PDZK1 expression. Hum. Mol. Genet. *27*, 3964–3973.
71. Yuan, X., Waterworth, D., Perry, J.R.B., Lim, N., Song, K., Chambers, J.C., Zhang, W., Vollenweider, P., Stirnadel, H., Johnson, T., et al. (2008). Population-based genome-wide association studies reveal six loci influencing plasma levels of liver enzymes. Am. J. Hum. Genet. *83*, 520–528.
72. Kamatani, Y., Matsuda, K., Okada, Y., Kubo, M., Hosono, N., Daigo, Y., Nakamura, Y., and Kamatani, N. (2010). Genome-wide association study of hematological and biochemical traits in a Japanese population. Nat. Genet. *42*, 210–215.
73. Chambers, J.C., Zhang, W., Sehmi, J., Li, X., Wass, M.N., Van der Harst, P., Holm, H., Sanna, S., Kavousi, M., Baumeister, S.E., et al. (2011). Genome-wide association study identifies loci influencing concentrations of liver enzymes in plasma. Nat. Genet. *43*, 1131–1138.
74. Gurdasani, D., Carstensen, T., Fatumo, S., Chen, G., Franklin, C.S., Prado-Martinez, J., Bouman, H., Abascal, F., Haber, M., Tachmazidou, I., et al. (2019). Uganda Genome Resource

Enables Insights into Population History and Genomic Discovery in Africa. Cell *179*, 984–1002.e36.
75. Seo, J.Y., Lee, J.E., Chung, G.E., Shin, E., Kwak, M.S., Yang, J.I., and Yim, J.Y. (2020). A genome-wide association study on liver enzymes in Korean population. PLoS ONE *15*, e0229374.
76. Pazoki, R., Vujkovic, M., Elliott, J., Evangelou, E., Gill, D., Ghanbari, M., van der Most, P.J., Pinto, R.C., Wielscher, M., Farlik, M., et al. (2021). Genetic analysis in European ancestry individuals identifies 517 loci associated with liver enzymes. Nat. Commun. *12*, 2579.
77. Dufour, D.R., Lott, J.A., Nolte, F.S., Gretch, D.R., Koff, R.S., and Seeff, L.B. (2000). Diagnosis and monitoring of hepatic injury. II. Recommendations for use of laboratory tests in screening, diagnosis, and monitoring. Clin. Chem. *46*, 2050–2068.
78. Astle, W.J., Elding, H., Jiang, T., Allen, D., Ruklisa, D., Mann, A.L., Mead, D., Bouman, H., Riveros-Mckay, F., Kostadima, M.A., et al. (2016). The Allelic Landscape of Human Blood Cell Trait Variation and Links to Common Complex Disease. Cell *167*, 1415–1429.e19.
79. Vuckovic, D., Bao, E.L., Akbari, P., Lareau, C.A., Mousas, A., Jiang, T., Chen, M.H., Raffield, L.M., Tardaguila, M., Huffman, J.E., et al. (2020). The Polygenic and Monogenic Basis of Blood Traits and Diseases. Cell *182*, 1214–1231.e11.
80. Fitzgerald, T., and Birney, E. (2021). CNest : A Novel Copy Number Association Discovery Method Uncovers 862 New Associations from 200,629 Whole Exome Sequence Datasets in the UK Biobank. Preprint at BioRxiv. https://doi.org/10.1101/2021.08.19.456963.
81. GTEx Consortium (2020). The GTEx Consortium atlas of genetic regulatory effects across human tissues. Science *369*, 1318–1330.
82. Avent, N.D., and Reid, M.E. (2000). The Rh blood group system: a review. Blood *95*, 375–387.
83. Nash, R., and Shojania, A.M. (1987). Hematological aspect of Rh deficiency syndrome: a case report and a review of the literature. Am. J. Hematol. *24*, 267–275.
84. Rai, D., Wilson, A.M., and Moosavi, L. (2021). Histology. Reticulocytes - StatPearls - NCBI Bookshelf. https://www.ncbi.nlm.nih.gov/books/NBK542172/.
85. Goldstein, D.E., Little, R.R., Lorenz, R.A., Malone, J.I., Nathan, D., and Peterson, C.M. (1995). Tests of glycemia in diabetes. Diabetes Care *18*, 896–909.
86. Akinlaja, O. (2016). Hematological Changes in Pregnancy - The Preparation for Intrapartum Blood Loss. Obstet. Gynecol. Int. J. *4*, 1–4.
87. Fry, A., Littlejohns, T.J., Sudlow, C., Doherty, N., Adamska, L., Sprosen, T., Collins, R., and Allen, N.E. (2017). Comparison of Sociodemographic and Health-Related Characteristics of UK Biobank Participants With Those of the General Population. Am. J. Epidemiol. *186*, 1026–1034.
88. Sanna, S., Busonero, F., Maschio, A., McArdle, P.F., Usala, G., Dei, M., Lai, S., Mulas, A., Piras, M.G., Perseu, L., et al. (2009). Common variants in the SLCO1B3 locus are associated with bilirubin levels and unconjugated hyperbilirubinemia. Hum. Mol. Genet. *18*, 2711–2718.
89. Johnson, A.D., Kavousi, M., Smith, A.V., Chen, M.H., Dehghan, A., Aspelund, T., Lin, J.P., van Duijn, C.M., Harris, T.B., Cupples, L.A., et al. (2009). Genome-wide association meta-analysis for total serum bilirubin levels. Hum. Mol. Genet. *18*, 2700–2710.
90. Kang, T.W., Kim, H.J., Ju, H., Kim, J.H., Jeon, Y.J., Lee, H.C., Kim, K.K., Kim, J.W., Lee, S., Kim, J.Y., et al. (2010). Genome-wide association of serum bilirubin levels in Korean population. Hum. Mol. Genet. *19*, 3672–3678.
91. Bielinski, S.J., Chai, H.S., Pathak, J., Talwalkar, J.A., Limburg, P.J., Gullerud, R.E., Sicotte, H., Klee, E.W., Ross, J.L., Kocher, J.P.A., et al. (2011). Mayo Genome Consortia: a genotype-phenotype resource for genome-wide association studies with an application to the analysis of circulating bilirubin levels. Mayo Clin. Proc. *86*, 606–614.
92. Dai, X., Wu, C., He, Y., Gui, L., Zhou, L., Guo, H., Yuan, J., Yang, B., Li, J., Deng, Q., et al. (2013). A genome-wide association study for serum bilirubin levels and gene-environment interaction in a Chinese population. Genet. Epidemiol. *37*, 293–300.
93. van de Steeg, E., Stránecký, V., Hartmannová, H., Nosková, L., Hřebíček, M., Wagenaar, E., van Esch, A., de Waart, D.R., Oude Elferink, R.P.J., Kenworthy, K.E., et al. (2012). Complete OATP1B1 and OATP1B3 deficiency causes human Rotor syndrome by interrupting conjugated bilirubin reuptake into the liver. J. Clin. Invest. *122*, 519–528.
94. Smith, N.F., Figg, W.D., and Sparreboom, A. (2005). Role of the liver-specific transporters OATP1B1 and OATP1B3 in governing drug elimination. Expert Opin. Drug Metab. Toxicol. *1*, 429–445.
95. van Paassen, B.W., van der Kooi, A.J., van Spaendonck-Zwarts, K.Y., Verhamme, C., Baas, F., and de Visser, M. (2014). PMP22 related neuropathies: Charcot-Marie-Tooth disease type 1A and Hereditary Neuropathy with liability to Pressure Palsies. Orphanet J. Rare Dis. *9*, 38.
96. Horowitz, G.L., and Staros, E.B. (2019). Creatinine: Reference Range (Interpretation, Collection and Panels). https://emedicine.medscape.com/article/2054342-overview#a2.
97. Cooper, D.N., Krawczak, M., Polychronakos, C., Tyler-Smith, C., and Kehrer-Sawatzki, H. (2013). Where genotype is not predictive of phenotype: towards an understanding of the molecular basis of reduced penetrance in human inherited disease. Hum. Genet. *132*, 1077–1130.
98. Chen, R., Shi, L., Hakenberg, J., Naughton, B., Sklar, P., Zhang, J., Zhou, H., Tian, L., Prakash, O., Lemire, M., et al. (2016). Analysis of 589,306 genomes identifies individuals resilient to severe Mendelian childhood diseases. Nat. Biotechnol. *34*, 531–538.
99. Wright, C.F., West, B., Tuke, M., Jones, S.E., Patel, K., Laver, T.W., Beaumont, R.N., Tyrrell, J., Wood, A.R., Frayling, T.M., et al. (2019). Assessing the Pathogenicity, Penetrance, and Expressivity of Putative Disease-Causing Variants in a Population Setting. Am. J. Hum. Genet. *104*, 275–286.
100. Goodrich, J.K., Singer-Berk, M., Son, R., Sveden, A., Wood, J., England, E., Cole, J.B., Weisburd, B., Watts, N., Caulkins, L., et al. (2021). Determinants of penetrance and variable expressivity in monogenic metabolic conditions across 77,184 exomes. Nat. Commun. *12*, 3505.
101. Day, F.R., Thompson, D.J., Helgason, H., Chasman, D.I., Finucane, H., Sulem, P., Ruth, K.S., Whalen, S., Sarkar, A.K., Albrecht, E., et al. (2017). Genomic analyses identify hundreds of variants associated with age at menarche and support a role for puberty timing in cancer risk. Nat. Genet. *49*, 834–841.
102. Karczewski, K.J., Francioli, L.C., Tiao, G., Cummings, B.B., Alföldi, J., Wang, Q., Collins, R.L., Laricchia, K.M., Ganna, A.,

Birnbaum, D.P., et al. (2020). The mutational constraint spectrum quantified from variation in 141,456 humans. Nature *581*, 434–443.
103. Su, Y.Q., Sugiura, K., Sun, F., Pendola, J.K., Cox, G.A., Handel, M.A., Schimenti, J.C., and Eppig, J.J. (2012). MARF1 regulates essential oogenic processes in mice. Science *335*, 1496–1499.
104. Kawaguchi, S., Ueki, M., and Kai, T. (2020). Drosophila MARF1 ensures proper oocyte maturation by regulating nanos expression. PLoS ONE *15*, e0231114.
105. Islam, R., Li, Y., Liu, X., Berihulay, H., Abied, A., Gebreselassie, G., Ma, Q., and Ma, Y. (2019). Genome-wide runs of homozygosity, effective population size, and detection of positive selection signatures in six chinese goat breeds. Genes (Basel) *10*, 1–24.
106. Katari, S., Aarabi, M., Kintigh, A., Mann, S., Yatsenko, S.A., Sanfilippo, J.S., Zeleznik, A.J., and Rajkovic, A. (2018). Chromosomal instability in women with primary ovarian insufficiency. Hum. Reprod. *33*, 531–538.
107. Yang, X., Touraine, P., Desai, S., Humphreys, G., Jiang, H., Yatsenko, A., and Rajkovic, A. (2019). Gene variants identified by whole-exome sequencing in 33 French women with premature ovarian insufficiency. J. Assist. Reprod. Genet. *36*, 39–45.
108. Su, Y.Q., Sun, F., Handel, M.A., Schimenti, J.C., and Eppig, J.J. (2012). Meiosis arrest female 1 (MARF1) has nuage-like function in mammalian oocytes. Proc. Natl. Acad. Sci. USA *109*, 18653–18660.
109. Gasner, A., and Rehman, A. (2021). Primary Amenorrhea - StatPearls - NCBI Bookshelf. https://www.ncbi.nlm.nih.gov/books/NBK554469/.
110. Walker, M.H., and Tobler, K.J. (2021). Female Infertility - StatPearls - NCBI Bookshelf. https://www.ncbi.nlm.nih.gov/books/NBK556033/.
111. Collins, R.L., Glessner, J.T., Porcu, E., Niestroj, L., Ulirsch, J., Kellaris, G., Howrigan, D.P., Everett, S., Mohajeri, K., Nuttle, X., et al. (2021). A cross-disorder dosage sensitivity map of the human genome. Preprint at MedRxiv. https://doi.org/10.1101/2021.01.26.21250098.
112. Miki, Y., Swensen, J., Shattuck-Eidens, D., Futreal, P.A., Harshman, K., Tavtigian, S., Liu, Q., Cochran, C., Bennett, L.M., Ding, W., et al. (1994). A strong candidate for the breast and ovarian cancer susceptibility gene BRCA1. Science *266*, 66–71.
113. McNally, E.J., Luncsford, P.J., and Armanios, M. (2019). Long telomeres and cancer risk: the price of cellular immortality. J. Clin. Invest. *129*, 3474–3481.
114. Fisher, R.A. (1918). The Correlation Between Relatives on the Supposition of Mendelian Inheritance. Trans. R. Soc. Edinb. *52*, 399–433.
115. Oetjens, M.T., Kelly, M.A., Sturm, A.C., Martin, C.L., and Ledbetter, D.H. (2019). Quantifying the polygenic contribution to variable expressivity in eleven rare genetic disorders. Nat. Commun. *10*, 4897.
116. Fahed, A.C., Wang, M., Homburger, J.R., Patel, A.P., Bick, A.G., Neben, C.L., Lai, C., Brockman, D., Philippakis, A., Ellinor, P.T., et al. (2020). Polygenic background modifies penetrance of monogenic variants for tier 1 genomic conditions. Nat. Commun. *11*, 3635.
117. Dauber, A., Yu, Y., Turchin, M.C., Chiang, C.W., Meng, Y.A., Demerath, E.W., Patel, S.R., Rich, S.S., Rotter, J.I., Schreiner, P.J., et al. (2011). Genome-wide association of copy-number variation reveals an association between short stature and the presence of low-frequency genomic deletions. Am. J. Hum. Genet. *89*, 751–759.
118. Wheeler, E., Huang, N., Bochukova, E.G., Keogh, J.M., Lindsay, S., Garg, S., Henning, E., Blackburn, H., Loos, R.J., Wareham, N.J., et al. (2013). Genome-wide SNP and CNV analysis identifies common and low-frequency variants associated with severe early-onset obesity. Nat. Genet. *45*, 513–517.
119. Männik, K., Mägi, R., Macé, A., Cole, B., Guyatt, A.L., Shihab, H.A., Maillard, A.M., Alavere, H., Kolk, A., Reigo, A., et al. (2015). Copy number variations and cognitive phenotypes in unselected populations. JAMA *313*, 2044–2054.
120. Saarentaus, E.C., Havulinna, A.S., Mars, N., Ahola-Olli, A., Kiiskinen, T.T.J., Partanen, J., Ruotsalainen, S., Kurki, M., Urpa, L.M., Chen, L., et al. (2021). Polygenic burden has broader impact on health, cognition, and socioeconomic outcomes than most rare and high-risk copy number variants. Mol. Psychiatry *26*, 4884–4895.
121. Sebat, J., Lakshmi, B., Malhotra, D., Troge, J., Lese-Martin, C., Walsh, T., Yamrom, B., Yoon, S., Krasnitz, A., Kendall, J., et al. (2007). Strong association of de novo copy number mutations with autism. Science *316*, 445–449.
122. Walsh, T., McClellan, J.M., McCarthy, S.E., Addington, A.M., Pierce, S.B., Cooper, G.M., Nord, A.S., Kusenda, M., Malhotra, D., Bhandari, A., et al. (2008). Rare structural variants disrupt multiple genes in neurodevelopmental pathways in schizophrenia. Science *320*, 539–543.
123. Stone, J.L., O'Donovan, M.C., Gurling, H., Kirov, G.K., Blackwood, D.H.R., Corvin, A., Craddock, N.J., Gill, M., Hultman, C.M., Lichtenstein, P., et al. (2008). Rare chromosomal deletions and duplications increase risk of schizophrenia. Nature *455*, 237–241.
124. Mefford, H.C., Muhle, H., Ostertag, P., von Spiczak, S., Buysse, K., Baker, C., Franke, A., Malafosse, A., Genton, P., Thomas, P., et al. (2010). Genome-wide copy number variation in epilepsy: novel susceptibility loci in idiopathic generalized and focal epilepsies. PLoS Genet. *6*, e1000962.
125. Mefford, H.C., Yendle, S.C., Hsu, C., Cook, J., Geraghty, E., McMahon, J.M., Eeg-Olofsson, O., Sadleir, L.G., Gill, D., Ben-Zeev, B., et al. (2011). Rare copy number variants are an important cause of epileptic encephalopathies. Ann. Neurol. *70*, 974–985.
126. McDaid, A.F., Joshi, P.K., Porcu, E., Komljenovic, A., Li, H., Sorrentino, V., Litovchenko, M., Bevers, R.P.J., Rüeger, S., Reymond, A., et al. (2017). Bayesian association scan reveals loci associated with human lifespan and linked biomarkers. Nat. Commun. *8*, 15842.
127. Freathy, R.M., Timpson, N.J., Lawlor, D.A., Pouta, A., Ben-Shlomo, Y., Ruokonen, A., Ebrahim, S., Shields, B., Zeggini, E., Weedon, M.N., et al. (2008). Common variation in the FTO gene alters diabetes-related metabolic traits to the extent expected given its effect on BMI. Diabetes *57*, 1419–1426.
128. Fall, T., Hägg, S., Mägi, R., Ploner, A., Fischer, K., Horikoshi, M., Sarin, A.P., Thorleifsson, G., Ladenvall, C., Kals, M., et al. (2013). The role of adiposity in cardiometabolic traits: a Mendelian randomization analysis. PLoS Med. *10*, e1001474.
129. Würtz, P., Wang, Q., Kangas, A.J., Richmond, R.C., Skarp, J., Tiainen, M., Tynkkynen, T., Soininen, P., Havulinna, A.S., Kaakinen, M., et al. (2014). Metabolic signatures of adiposity

in young adults: Mendelian randomization analysis and effects of weight change. PLoS Med. *11*, e1001765.

130. Barker, D.J.P., Hales, C.N., Fall, C.H.D., Osmond, C., Phipps, K., and Clark, P.M.S. (1993). Type 2 (non-insulin-dependent) diabetes mellitus, hypertension and hyperlipidaemia (syndrome X): relation to reduced fetal growth. Diabetologia *36*, 62–67.
131. Armengaud, J.B., Yzydorczyk, C., Siddeek, B., Peyter, A.C., and Simeoni, U. (2021). Intrauterine growth restriction: Clinical consequences on health and disease at adulthood. Reprod. Toxicol. *99*, 168–176.
132. Halvorsen, M., Huh, R., Oskolkov, N., Wen, J., Netotea, S., Giusti-Rodriguez, P., Karlsson, R., Bryois, J., Nystedt, B., Ameur, A., et al. (2020). Increased burden of ultra-rare structural variants localizing to boundaries of topologically associated domains in schizophrenia. Nat. Commun. *11*, 1842.
133. Chen, L., Abel, H.J., Das, I., Larson, D.E., Ganel, L., Kanchi, K.L., Regier, A.A., Young, E.P., Kang, C.J., Scott, A.J., et al. (2021). Association of structural variation with cardiometabolic traits in Finns. Am. J. Hum. Genet. *108*, 583–596.
134. Li, J., Yang, T., Wang, L., Yan, H., Zhang, Y., Guo, Y., Pan, F., Zhang, Z., Peng, Y., Zhou, Q., et al. (2009). Whole genome distribution and ethnic differentiation of copy number variation in Caucasian and Asian populations. PLoS ONE *4*, e7958.
135. Campbell, C.D., Sampas, N., Tsalenko, A., Sudmant, P.H., Kidd, J.M., Malig, M., Vu, T.H., Vives, L., Tsang, P., Bruhn, L., and Eichler, E.E. (2011). Population-genetic properties of differentiated human copy-number polymorphisms. Am. J. Hum. Genet. *88*, 317–332.
136. Chen, W., Hayward, C., Wright, A.F., Hicks, A.A., Vitart, V., Knott, S., Wild, S.H., Pramstaller, P.P., Wilson, J.F., Rudan, I., and Porteous, D.J. (2011). Copy number variation across European populations. PLoS ONE *6*, e23087.
137. Martin, C.L., Wain, K.E., Oetjens, M.T., Tolwinski, K., Palen, E., Hare-Harris, A., Habegger, L., Maxwell, E.K., Reid, J.G., Walsh, L.K., et al. (2020). Identification of Neuropsychiatric Copy Number Variants in a Health Care System Population. JAMA Psychiatry *77*, 1276–1285.

The American Journal of Human Genetics, Volume *109*

Supplemental information

The individual and global impact of copy-number variants on complex human traits

Chiara Auwerx, Maarja Lepamets, Marie C. Sadler, Marion Patxot, Miloš Stojanov, David Baud, Reedik Mägi, Estonian Biobank Research Team, Eleonora Porcu, Alexandre Reymond, and Zoltán Kutalik

SUPPLEMENTAL FIGURES

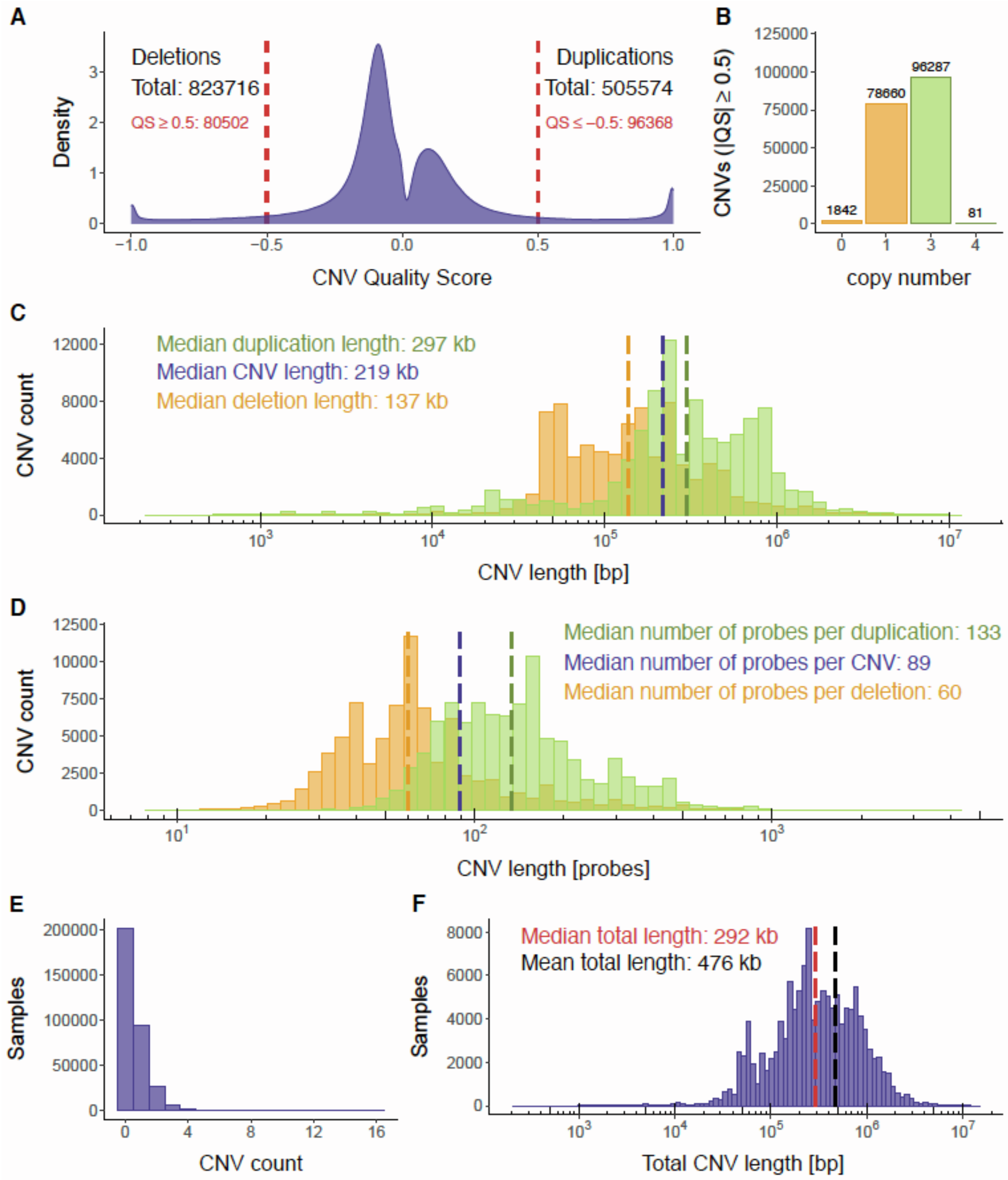

Figure S1. Distribution of high confidence CNVs in the UK Biobank

(A) Density plot of quality scores (QSs) for the 1’329’290 called CNVs. High confidence duplications (QS ≥ 0.5) and deletions (QS ≤ -0.5), indicated per the red dashed lines, were retained for downstream analyses. (B) Distribution of the copy number state of high confidence CNVs. Deletions (copy number 0 or 1) are in orange, duplications (copy number 3 or 4) are in green. Number of CNVs in each category is indicated on top of the bars. Distribution of high confidence duplications (green) and deletions (orange) length in base pairs (C) and number of probes (D) on a logarithmic scale. Dashed lines show the median duplication (green), CNV (purple), and deletion (orange) length. (E) Distribution of high confidence CNV counts per individual. (F) Distribution of the total amount of bases affected by high confidence CNVs per individual on a logarithmic scale; red and black dashed lines show the median and mean number of bases affected by CNVs among individuals with at least 1 CNV, respectively.

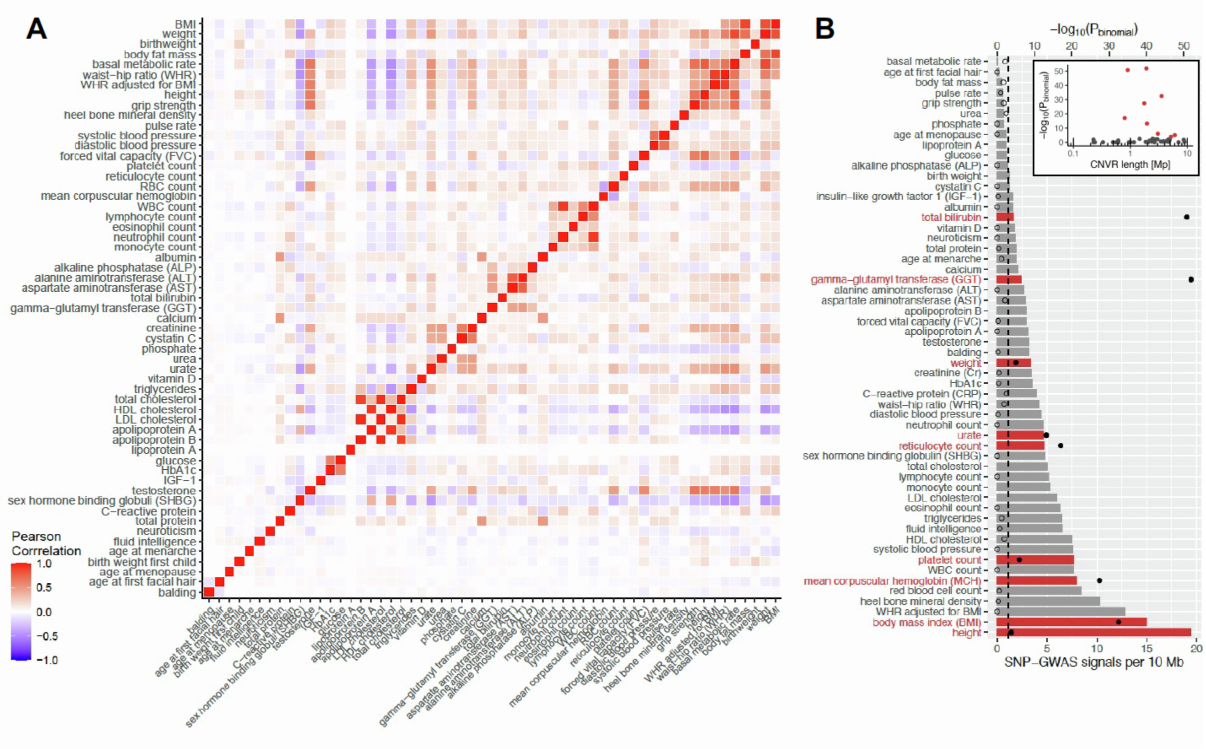

Figure S2. Genome-wide association studies of complex traits

(A) Pearson correlation across the 57 continuous traits assessed by CNV-GWAS. (B) Number of autosomal NHGRI-EBI GWAS Catalog (Web Resources)[1] SNP-GWAS signals per 10 Mb of autosomal genome (bottom x-axis; as horizontal bars) for 56 of the assessed traits and their synonyms (y-axis; Table S1). The top x-axis indicates the negative logarithm of the *p*-value (as a dot) for the binomial test that assesses the SNP-GWAS signal distribution within CNV regions, as compared to the entire autosomal genome. Non-significant traits are in grey with an empty dot; significant traits ($p \leq 0.05/56$; above the black dashed line) are in red with full dots. The inset shows the negative logarithm of the binomial test's *p*-value against the total length of trait-associated CNVRs in Mb on a logarithmic scale for each trait. Significant traits ($p \leq 0.05/56$) are in red, non-significant ones in grey.

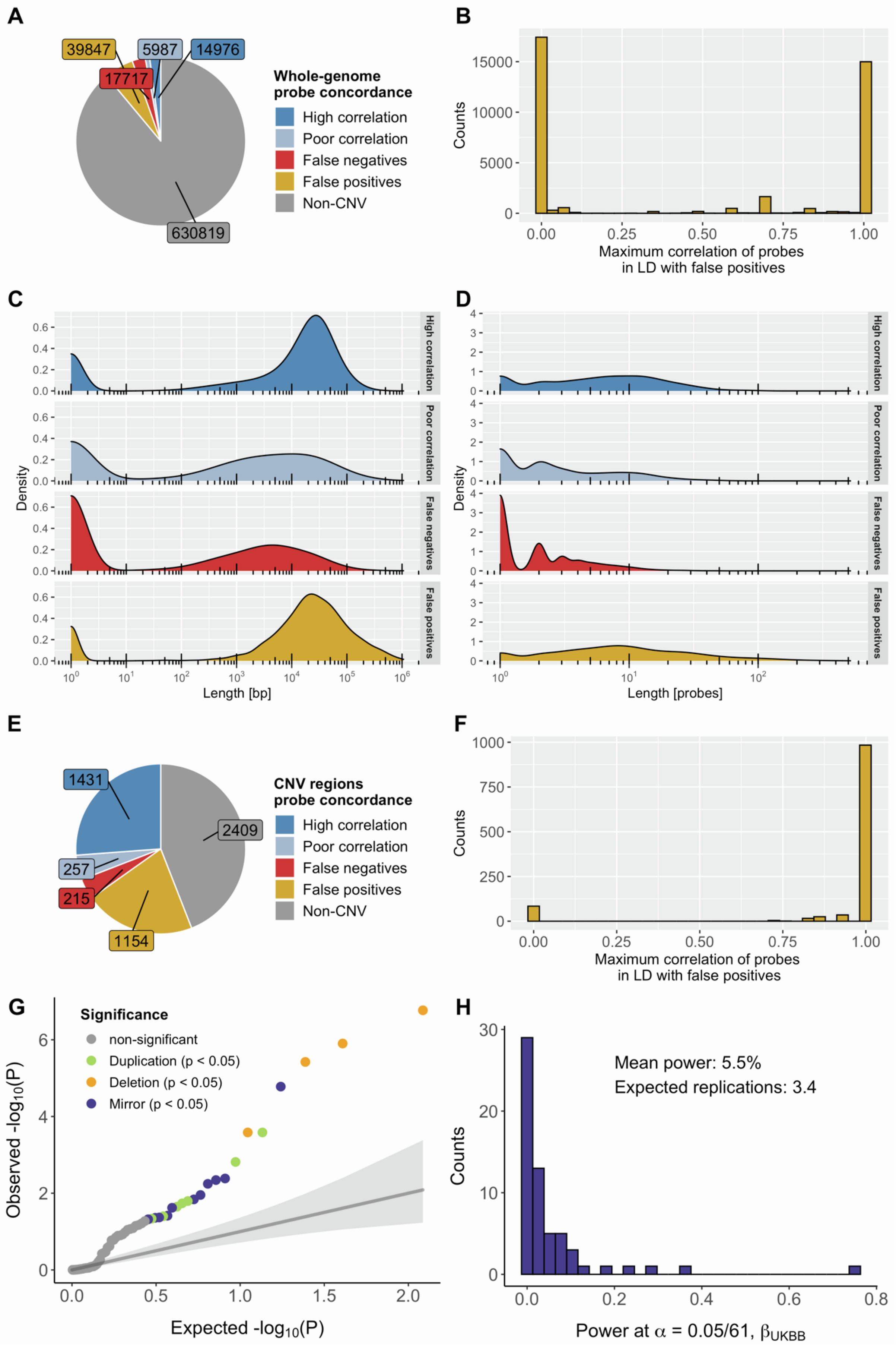

Auwerx et al., 2022

Figure S3. Replication in the Estonian Biobank

(A) Concordance between PennCNV (microarray) and STRiP (whole-genome sequencing) CNV calls for 709’358 quality-controlled, autosomal probes from the OmniExpress-24 genotyping array in the Estonian Biobank (EstBB). Probes with high ($r \geq 0.75$) and low ($r < 0.75$) correlation between PennCNV and STRiP CNV profiles are in dark and light blue, respectively; false negatives (*i.e.* probes only detected in a CNV state by STRiP) are in red; false positives (*i.e.* probes only detected in a CNV state by PennCNV) are in gold; monomorphic probes are in grey. (B) Distribution of the maximal PennCNV-STRiP correlation among probes in linkage disequilibrium (± 250 kb and $r \geq 0.5$) with false positive probes from (A). Size distribution in base pairs (C) and probes (D) of consecutive stretches of probes mapping to non-monomorphic categories in (A). (E) EstBB concordance between PennCNV and STRiP CNV calls at 5’566 probes overlapping UK Biobank trait-associated CNV regions; identical color scheme to (A). (F) Distribution of the maximal PennCNV-STRiP correlation among probes in linkage disequilibrium (± 250 kb and $r \geq 0.5$) with false positive probes from (E). (G) Expected versus observed negative logarithm of *p*-values for the 61 CNV-trait pairs assessed in the EstBB. Directionally concordant and nominally significant ($p \leq 0.05$) signals replicated with the duplication-only, deletion-only, or mirror association model are in green, orange, or purple, respectively. Non-significant signals ($p > 0.05$) are in grey. Shade represents the 95% confidence interval around expected values. (H) Distribution of replication power at $\alpha = 0.05/61 = 8.2x10^{-4}$, assuming similar effect sizes to the one observed in the UKBB, for the 61 signals assessed in (G). The mean power is 5.5%, corresponding to an expected number of multiple testing correction surviving replications of 0.055*61 = 3.4.

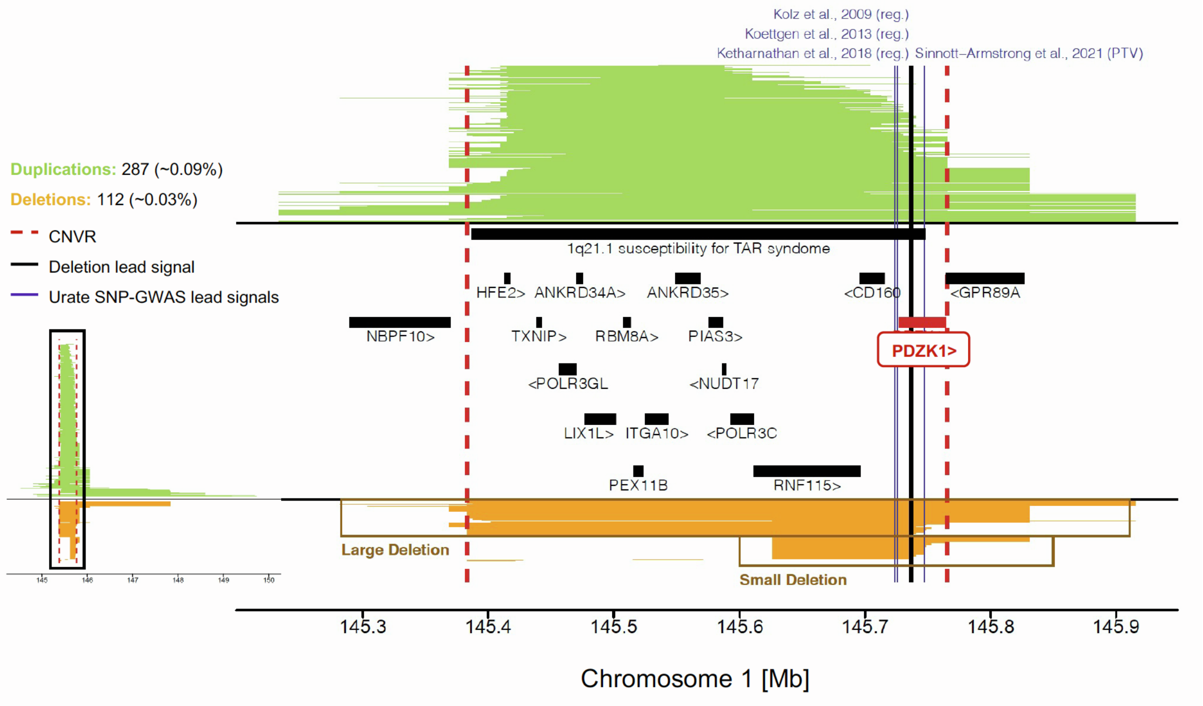

Figure S4. 1q21.1 deletion and decreased serum urate levels

Mapping of CNVs overlapping the 1q21.1 (chr1:145'383'239-145'765'206) deletion region associated with decreased serum urate levels. Number and frequency of duplications and deletions are at the top left; left plot shows all overlapping CNVs; right plot focuses on the central CNV region represented by red dashed lines. Duplications are in green, deletions in orange; black line indicates the lead signal for serum urate (deletion-only); purple lines indicate serum urate-associated SNPs[2–5] (reg. = regulatory variant; PTV = protein-truncating variant). DECIPHER recurrent CNV and overlapping protein-coding genes are in black, except for *PDZK1* in red. Brown boxes separate large (start < 145.6 Mb) versus small (start ≥ 145.6 Mb) deletion carriers.

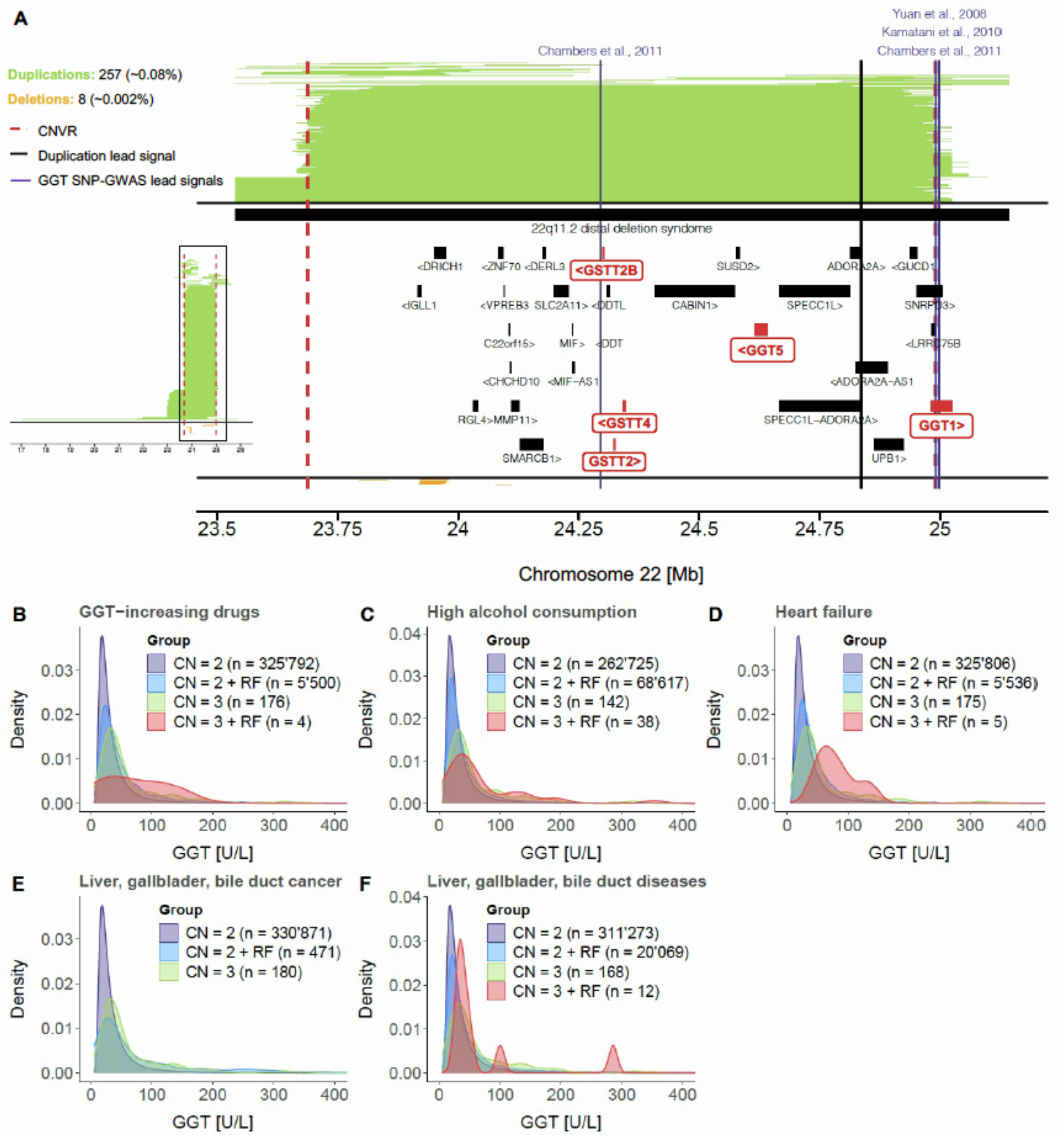

Figure S5. 22q11.23 duplication and increased serum γ-glutamyl transferase levels

(A) Mapping of CNVs overlapping the 22q11.23 (chr22:23'688'345-24'990'213) duplication associated with increased γ-glutamyl transferase (GGT) levels. Number and frequency of duplications and deletions are at the top left; left plot shows all overlapping CNVs; right plot focuses on the central CNV region represented by red dashed lines. Duplications are in green, deletions in orange; black line indicates the lead signal for GGT (duplication-only); purple lines indicate GGT-associated SNPs[6–8]. DECIPHER recurrent CNV (truncated) and overlapping protein-coding genes are in black, except for genes involved in glutathione metabolism in red. (B-F) Density plots showing the distribution of GGT levels in copy-neutral (CN = 2) and 22q11.23 (chr22:23'688'345-24'990'213) overlapping duplication carriers (CN = 3) with or without various risk factors (RF) for increased GGT: (B) GGT-increasing drugs, (C) high alcohol consumption, (D) heart failure, and (E) cancer or (F) other diseases of the liver, gallbladder, or bile ducts. The sample size (n) for each category is indicated. Plots were truncated to 400 U/L (maximum value: 1167 U/L).

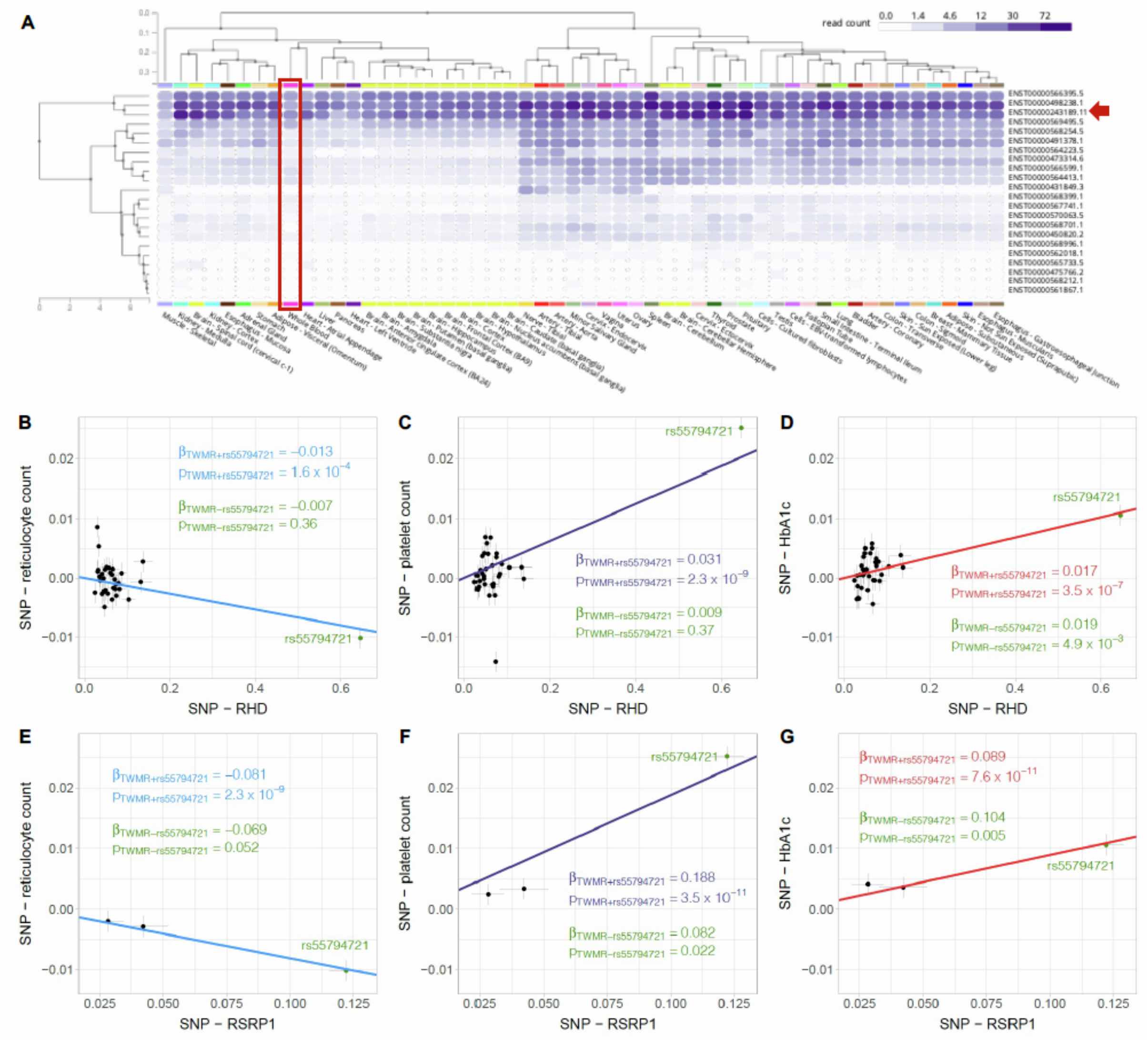

Figure S6. 1p36.11 deletion and altered hematological traits

(A) GTEx v8 (Web Resources)[9] isoform expression for *RSRP1* in 54 tissues. Whole blood is circled in red. The red arrow points at the isoform with the highest expression in whole blood, ENST00000243189. (B-G) SNP-exposure (x-axis) versus SNP-outcome (y-axis) plots for transcriptome-wide Mendelian randomization (TWMR) analyses estimating the causal effect of (B) *RHD* → reticulocyte count, (C) *RHD* → platelet count, (D) *RHD* → glycated hemoglobin (HbA1c), (E) *RSRP1* → reticulocyte count, (F) *RSRP1* → platelet count, and (G) *RSRP1* → HbA1c. Grey horizontal and vertical lines represent the standard errors around the SNP-exposure and SNP-outcome estimates, respectively. Causal effect estimates (β) and associated *p*-values are reported for each analysis. The outlier SNP rs55794721 is in green. Causal effects were re-estimated omitting rs55794721 and are reported in green.

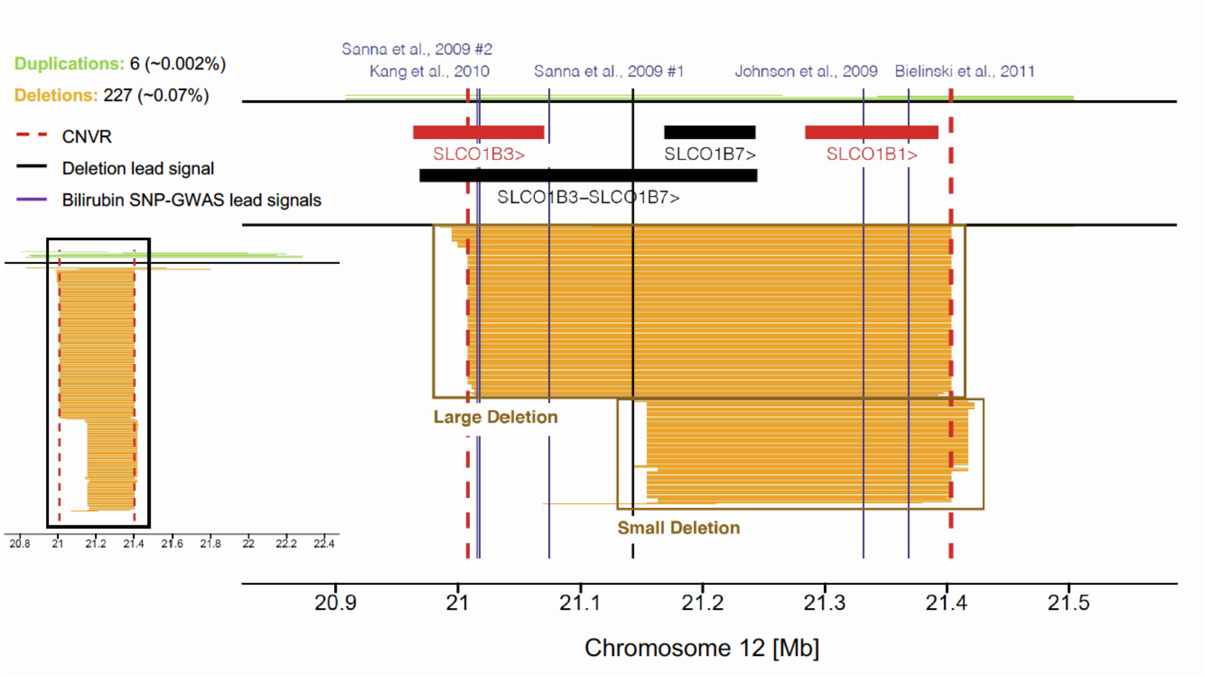

Figure S7. 12p12.2-p12.1 deletion and increased total bilirubin levels

Mapping of CNVs overlapping the 12p12.2-12p12.1 (chr12:21'008'080-21'403'457) deletion associated with increased total bilirubin levels. Number and frequency of duplications and deletions are at the top left; left plot shows all overlapping CNVs; right plot focuses on the central CNV region represented by red dashed lines. Duplications are in green, deletions in orange; black line indicates the lead signal for total bilirubin (deletion-only); purple lines indicate serum bilirubin-associated SNPs[10–13]; overlapping protein-coding genes are shown in black, except for Rotor syndrome-associated genes, *SLCO1B1* and *SLCO1B3*, shown in red. Brown boxes separate large (start < 21.1 Mb) versus small (start ≥ 21.1 Mb) deletion carriers.

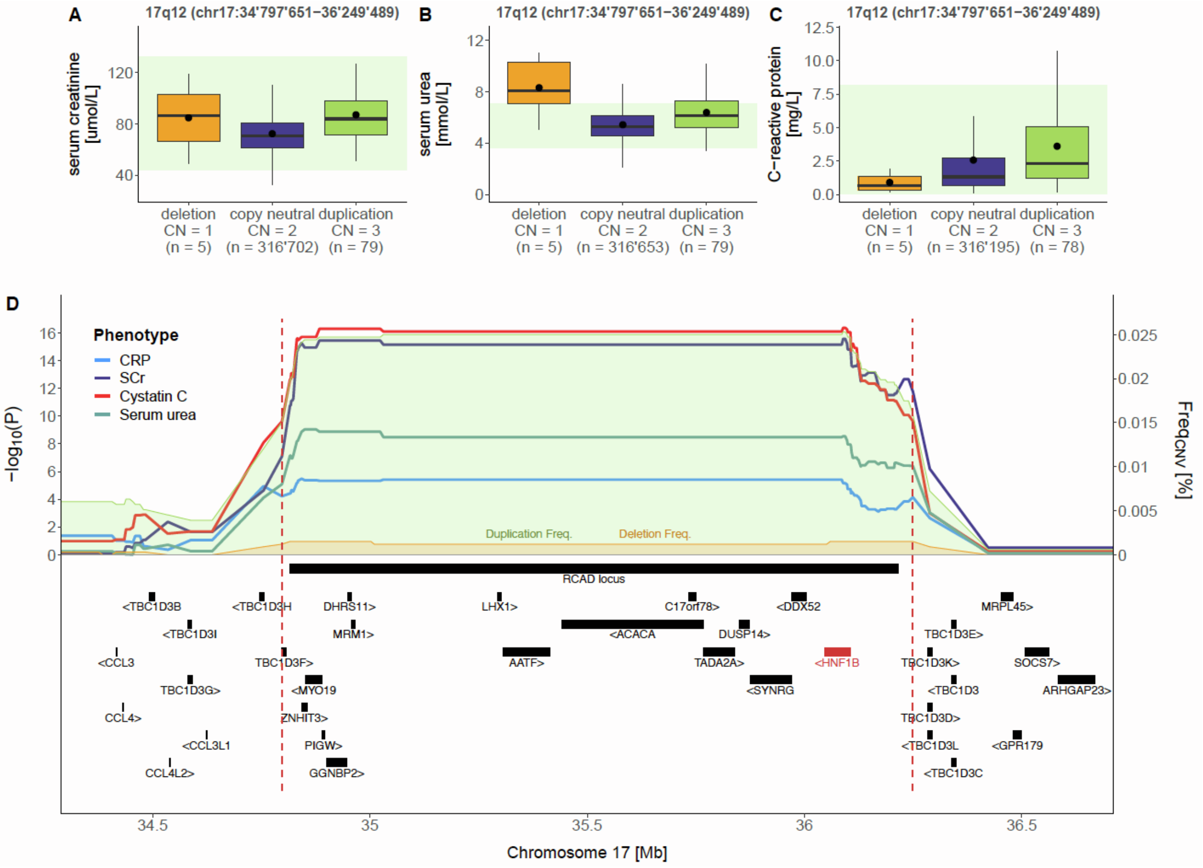

Figure S8. 17q12 CNVs and renal phenotypes

Boxplots representing levels of (A) serum creatinine (SCr), (B) serum urea, and (C) C-reactive protein (CRP) in individuals with a 17q12 (chr17:34'797'651-36'249'489) overlapping deletion, copy-neutrality, or duplication. Copy number (CN) and sample size (n) are reported for each category; dots show the mean; outliers are not shown; green bands show normal clinical range for (A) SCr: 44.2-132.6 µmol/L, (B) serum urea: 3.6-7.1 mg/L, and (C) CRP: 0.07-8.2 mg/L. (D) Association plot for the 17q12 (chr17:34'797'651-36'249'489) CNV region. Red dashed lines delimit the duplication-only associated CNV region; left y-axis shows the negative logarithm of association *p*-value for CRP (blue), SCr (purple), cystatin C (red), and serum urea (turquoise); right y-axis shows CNV frequency [%], with duplication frequency in green and deletion frequency in orange; overlapping DECIPHER recurrent CNV and genes are in black, except for the putative causal gene, *HNF1B*, in red.

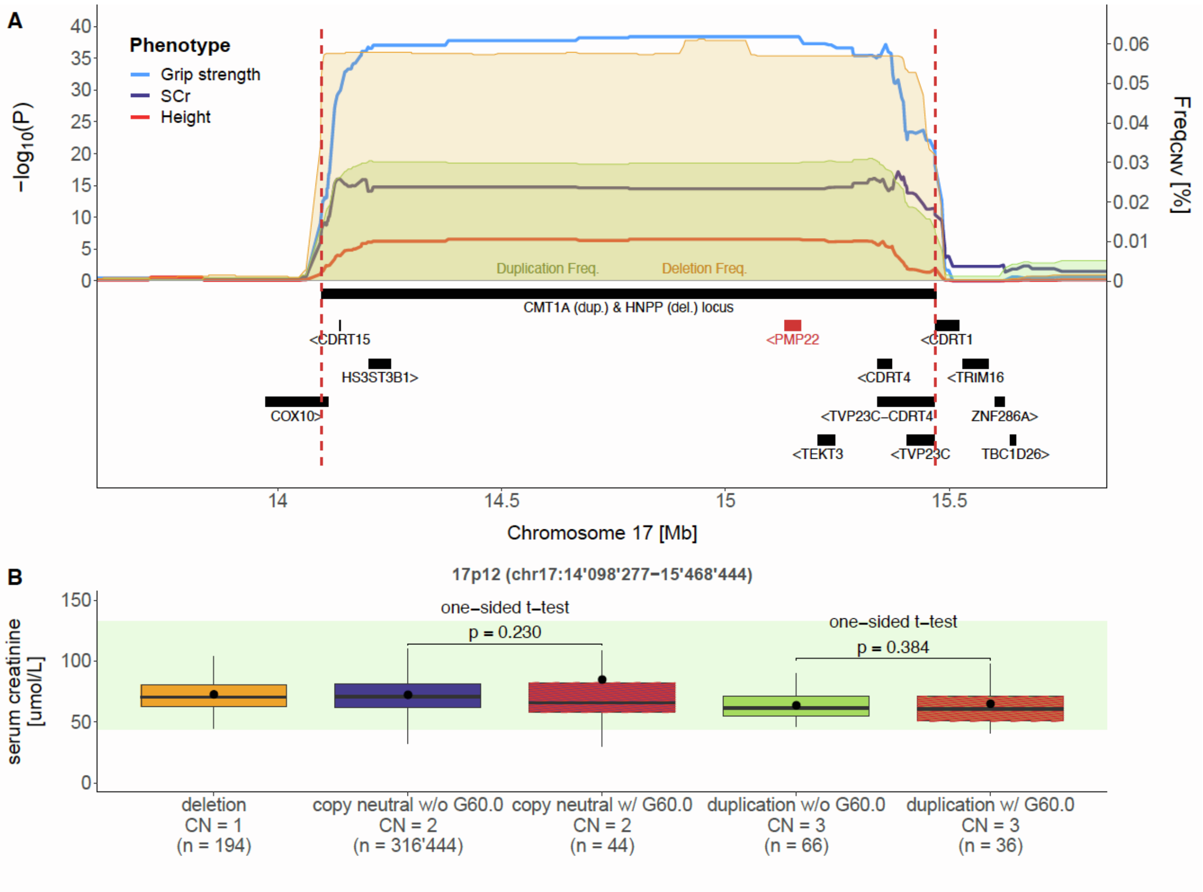

Figure S9. 17p12 duplication and muscle phenotypes

(A) Association plot for the 17p12 (chr17:14’098’277-15’468’444) overlapping CNV region. Red dashed lines delimit the duplication-only associated CNV region; left y-axis represents the negative logarithm of association *p*-values for hand grip strength (blue), serum creatinine (SCr; purple), and height C (red); right y-axis represents CNV frequency [%], with duplication frequency in green and deletion frequency in orange; overlapping DECIPHER recurrent CNV and genes are in black, except for the putative causal gene, *PMP22*, in red. (B) Boxplots representing SCr levels of individuals with a 17p12 (chr17:14’098’277-15’468’444) overlapping deletion, copy-neutrality, or duplication, the two latter being split according to the presence (w/) or absence (w/o) of a hereditary motor or sensory neuropathy diagnosis (ICD-10 G60.0; red stripes). Copy number (CN) and sample size (n) are reported for each category; dots show the mean; outliers are not shown; green bands show normal clinical range for SCr: 44.2-132.6 μmol/L.

SUPPLEMENTAL MATERIAL AND METHODS

Note 1: Cohort descriptions

The UK Biobank cohort: The UK Biobank (UKBB) is a volunteer-based cohort of ~500'000 individuals (54% females) from the general UK population[14]. Individuals were aged 40-69 years at recruitment and underwent microarray-based genotyping and extensive phenotyping, which is constantly extended and includes physical measurements, blood biomarker analyses, socio-demographic and health-related questionaries, as well as linkage to medical health records. Participants signed a broad informed consent form and data was accessed through the application number 16389.

The Estonian Biobank cohort: The Estonian Biobank (EstBB) is a population-based cohort encompassing ~20% of Estonia's adult population (~200'000 individuals; 66% females; Web Resources)[15]. Individuals underwent microarray-based genotyping at the Core Genotyping Lab of the Institute of Genomics, University of Tartu, and a subset of ~2'500 samples underwent whole-genome sequencing (WGS). General data, including body measurements, were collected at recruitment. Project-based questionnaires were sent later and filled on a voluntary basis. Health records are updated through linkage with the national Health Insurance Fond and other relevant databases, providing sporadic access to blood biomarker measurements and medical diagnoses. All participants signed a broad informed consent form and analyses were carried out under ethical approval 1.1-12/624 from the Estonian Committee on Bioethics and Human Research and data release N05 from the EstBB.

The CHUV maternity cohort: The Lausanne University Hospital (CHUV) maternity cohort was designed as a serological surveillance study of maternal toxoplasmosis infections and approval from the Ethics Committee of Vaud (CER-VD) was obtained for data reusage under the project ID 2019-00280 to investigate maternal and fetal outcomes. Rhesus (Rh) blood

groups were serologically determined for 5'164 women. Reticulocyte count, platelet count, glycated hemoglobin (HbA1c) levels, intrapartum reports, and International Classification of Diseases, 10th Revision (ICD-10) codes were sporadically collected between 2009-2014.

Note 2: CNV calling and quality score pipeline

CNV calling: Autosomal and pseudoautosomal copy number variant (CNV) calling was performed in parallel for the 106 genotyping batches using PennCNV[16]. Individual intensity files were generated from the B allele frequency (BAF) and Log R Ratio (LRR) files available on the UKBB portal. Missing values (-1) were set to NA. Batch-specific population frequency of the B allele (PFB) files were generated. Probes with missing PFB were removed in a batch-specific way. The hidden Markov Model file for Affymetrix genome-wide 6.0 array was downloaded as part of the PennCNV-Affy package and used without training. The GC model file was generated following instructions of `cal_gc_snp.pl`, using gc5Base downloaded from the UCSC Genome Browser (03/2020; Web Resources). Above-described files were used to call CNVs with confidence score using `detect_cnv.pl` with genomic wave adjustment. Adjacent CNVs (gap ≤ 20% of merged CNV length) were merged with `clean_cnv.pl`. Chromosome X CNVs were called separately with the PennCNV inbuilt arguments `-chrx` and `-sexfile` when running `detect_cnv.pl`. Copy-neutral losses of heterozygosity resulting from male chromosome X hemizygosity were excluded, and adjacent CNVs were merged.

Quality score pipeline: Hurdles linked to CNV analysis include high false positive rates and variability in break points. To mitigate these issues, a post-PennCNV processing pipeline was used to attribute a quality score (QS) to each CNV and transform calls to the probe level[17,18]. QSs range from -1 to 1 and reflect the probability of a CNV to be a true positive (-1 = likely deletion; 1 = likely duplication; ~0 = low confidence CNVs). Briefly, PennCNV `filter_cnv.pl` was run separately on autosomal/pseudoautosomal and chromosome X

CNVs (with `-chrx`), resulting in two sample-level quality control (QC) files. These were combined by adding the number of chromosome X CNVs to the autosomal/pseudoautosomal sample-level QC file. A single file containing all called CNVs, as well as associated CNV- and sample-level QC metadata, was generated and used to attribute a QS to each called CNV. Next, linear PennCNV coordinates were transformed into per-chromosome $probe\ x\ sample$ matrices, with entries reflecting the QS attributed to the CNV mapping to these probes. Copy neutral probes are indicated by 0 and individuals with no CNVs were added as all-0 columns.

Note 3: CNV association studies in the UKBB

Number of effective tests: As retained CNV-proxy probes remain highly correlated – much more so than SNPs would be due to classical linkage disequilibrium patterns – the number of effective tests, N_{eff}, was determined[18,19]. Per-chromosome $probe\ x\ sample$ genotype matrices G were generated, with genotypes taking values of -1 (deletion), 0 (copy-neutral), and 1 (duplication). Chromosome-wise N_{eff} were defined as the number of eigenvalues required to explain 99.5% of the variance in G and were summed up, resulting in a genome-wide (GW) N_{eff}. N_{eff} was estimated at 11'804, setting the GW threshold for significance at $p \leq 0.05/11'804 = 4.2 \times 10^{-6}$.

Phenotype selection: Fifty-seven continuous traits were selected based on data availability and presumed high heritability. Fifty-four were defined as the mean of measured instances. Three were composite traits: Grip strength, as the mean of *hand grip strength left* and *right* (#46, #47); waist-to-hip ratio (WHR), as the ratio between *waist* and *hip circumference* (#48, #49); WHR adjusted for body mass index (WHRadjBMI), by correcting WHR for BMI. Two were male-specific (*relative age of first facial hair* (#2375); *hair/balding pattern* (#2395)) and three were female-specific (*age when periods started (menarche)* (#2714); *age at menopause (last menstrual period)* (#3581); *birth weight of first child* (#2744)). Entries "do not know", "only had twins", "prefer not to answer" were set as missing.

Stepwise conditional analysis: Stepwise conditional analysis was performed on CNV-GWAS results to determine the number of independent signals per trait. For traits with ≥ 1 GW-significant signal, CNV information was extracted for the lead probe, with genotypes taking values of -1 (deletion), 0 (neutral), and 1 (duplication) for the mirror model, and setting deletions or duplications to NA when considering the duplication-only or deletion-only models, respectively. Lead CNV probe effect was regressed out of the phenotype and association studies were conducted anew. These steps were repeated until no GW-associated probes were identified.

Note 4: Replication in the Estonian Biobank

Comparative analysis of CNV quality

CNV detection in OmniExpress-24 genotype data: About 7’750 EstBB participants were genotyped with Illumina Infinium OmniExpress-24 genotyping array. Samples with genotype call rate < 98%, Hardy-Weinberg equilibrium test *p*-value < $1x10^{-4}$, or mismatched sex based on chromosome X heterozygosity were excluded. Intensity files (LRR and BAF) were created with Illumina GenomeStudio v2.0.4. A PFB file was generated from all samples. Only autosomal probes were carried over to the CNV detection step (709’358 probes), which was performed with PennCNV[16] analogously to what has been described for the UKBB. Samples with > 200 CNVs or a total length of CNV calls > 10 Mb were excluded. CNVs were attributed a QS, as previously described for the UKBB, and CNVs with |QS| ≥ 0.5 were retained. To harmonize data with WGS-based CNV calls, duplications < 2 kb and deletions < 1 kb were excluded.

CNV detection in WGS data: WGS data (30x) was available for ~2’500 EstBB samples. WGS-based autosomal CNVs were called in 5 batches using the Genome STRiP pipeline[20]. Eleven samples with $calls/sample > median + 3 * median\ absolute\ deviation$ were

removed. The union of the discovered sites was genotyped with Genome STRiP SVGenotyper module in all batches separately and merged. Duplicate calls were removed using standard Genome STRiP duplicate removal settings (overlap > 50% and duplicate score > 0). Low-quality CNVs and CNVs with call rate < 90% were excluded. Duplications < 2 kb and deletions < 1 kb were excluded. To harmonize data with microarray-based CNV calls, CNVs > 10 Mb were excluded and adjacent CNVs were merged (gap ≤ 20% of merged CNV length).

CNV calling and copy number association studies

Global Screening Array (GSA) genotype data: Twelve batches containing 202’282 EstBB participants were genotyped with Illumina GSAv1.0, GSAv2.0, GSAv2.0_ESTChip, and GSAv3.0_ESTChip2. Samples with genotype call rate < 98%, Hardy-Weinberg equilibrium test *p*-value < $1x10^{-4}$, or mismatched sex based on chromosome X heterozygosity were excluded and one of each duplicated sample was retained. Genotypes were re-clustered by manual realignment of cluster location. Intensity files (LRR and BAF) were created with Illumina GenomeStudio v2.0.4. A PFB file was generated from 1’000 randomly selected samples from batch 1. Only autosomal probes overlapping all GSA versions (excluding custom ESTChip probes) were carried over to the CNV detection step (671’035 probes; 242’091 probes overlap with UKBB).

Sample quality control: Samples originating from 2 batches with outlier genotyping intensity parameters, as well as genotyping plates with > 3 samples with either > 200 called CNV or a total length of CNV calls > 10 Mb were excluded. Individual samples meeting these criteria were removed. Among related pairs (KING kinship coefficient > 0.0884), the sample with most available phenotypes was retained.

Power analysis: Simulations were conducted to estimate the power of our replication study. We defined $\beta_{i,j}$ as the standardized effect of probe i on trait j observed in the UKBB, $q_{i,dup}$ and

$q_{i,del}$ the duplication and deletion frequencies of probe i in the EstBB, respectively, and N_j the sample size for trait j in the EstBB. When considering mirror signals, CNVs were simulated for N_j samples as:

$$CNV = \begin{cases} -1, & z \leq q_{i,del} \\ 0, & q_{i,del} < z < 1 - q_{i,dup} \\ 1, & z \geq 1 - q_{i,dup} \end{cases}$$

With $z \sim U(0,1)$. For duplication-only or deletion-only signals, duplications or deletions were simulated for N_j samples as $CNV \sim Bernoulli(q_{i,dup})$ or $CNV \sim Bernoulli(q_{i,del})$, respectively. Normally distributed error terms ε were simulated according to $\varepsilon \sim N(0, \sigma^2)$ for N_j samples. For mirror signals, the noise variance σ^2 was defined as $\sigma^2 = \sigma_j^2 - var(CNV) * \beta_{i,j}^2$, with σ_j^2 the observed standardized variance for trait j in the EstBB equaling 1. For duplication-only and deletion-only signals, σ^2 was defined as $\sigma^2 = \sigma_j^2 - q_{i,dup} * (1 - q_{i,dup}) * \beta_{i,j}^2$ and $\sigma^2 = \sigma_j^2 - q_{i,del} * (1 - q_{i,del}) * \beta_{i,j}^2$, respectively. Phenotypes Y were simulated for N_j samples as $Y = CNV * \beta_{i,j} + \varepsilon$. When simulated data contained ≥ 1 CNV carrier, the *p*-value for the estimated effect size from the linear regression $Y \sim CNV$ was computed and retained. Otherwise, the *p*-value was set as missing. For each signal, 10'000 simulations were conducted, and power was defined as the fraction of non-missing *p*-values ≤ 8.2×10^{-4}.

Note 5: Extended phenotypic assessment

Disease diagnoses: To assess patients' disease status, ICD-10 diagnoses were used (#41270):

- γ-glutamyl transferase (GGT)-altering diseases: *heart failure* (I50), *malignant neoplasm of liver and intrahepatic bile ducts* (C22), gallbladder (C23), and *other unspecified parts of biliary tract* (C24), as well as other *diseases of the liver* (K70-K77) and the *gallbladder, biliary tract, or pancreas* (K80-K87) were considered.

- Rotor syndrome: Rotor syndrome [MIM: 237450] is classified with Dubin-Johnson syndrome [MIM: 237500] under *other disorders of bilirubin metabolism* (E80.6).
- Charcot-Marie-Tooth type 1A: CMT1A [MIM:118220] is classified as *hereditary motor or sensory neuropathy* (G60.0), a diagnosis encompassing all forms of CMT and related neuropathies.

Lifestyle:

- Self-reported high alcohol consumption (#1558): *daily or almost daily*.
- GGT-increasing drug usage[21] (#20003): 2038459704 (carbamazepine), 1140865426 (cimetidine), 1140909708 (furosemide), 1140869848 (methotrexate), 1140910706 (phenobarbital), 2038460076 (phenytoin).

Socio-economic factors:

- *Townsend deprivation index at recruitment* (#189).
- *Average total household income before tax* (#738): averaged over measured instances.
- *Age completed full time education* (#845): proxy for educational attainment (EA); averaged over measured instances.

Life history traits:

- *Age at recruitment* (#21022)
- *Mother's* (#3526) and *father's* (#1807) *age at death:* averaged over measured instances; meta-analyzed as parental lifespan.
- *Relative leucocyte telomere length adjusted for the influence of technical parameters* (#22191): averaged over measured instances.

For socio-economic factors and life history traits, entries matching "do not know" or "prefer not to answer" were set as missing. Traits were inverse normal transformed prior correction for sex, age (#21003), age^2, genotyping batch, and principal components (PCs) 1-40, except for *age at recruitment*, which was not corrected for age and age^2.

Note 6: *RHD* and hematological traits

Transcriptome-wide Mendelian Randomization: Using univariable transcriptome-wide Mendelian randomization[22] (TWMR), the causal effect of differential *RHD* and *RSRP1* expression on reticulocyte count, platelet count, and HbA1c was estimated based on independent ($r^2 < 0.01$) genetic variants. Expression quantitative trait loci (eQTLs) were obtained from the eQTLGen consortium and included *cis*-eQTLs (FDR < 0.05, 2-cohort filter) for ~16,900 transcripts[23]. GWAS effect sizes originate from the Neale Lab UKBB summary statistics (Web Resources). Exposure and outcome datasets were harmonized, standardized effect size estimates were obtained by dividing z-scores by the square root of the sample size, and palindromic variants and variants with allele frequency difference > 5% between the two datasets were removed.

Association between Rhesus blood group and hematological traits: Impact of Rh^- blood group on platelet count, reticulocyte count, and HbA1c was assessed in the CHUV maternity cohort through multivariate linear regression that incorporates the covariates: age at measurement, gestational week at measurement, whether the woman was pregnant at measurement (57.5% for reticulocyte count, 35.6% for platelet count, 23.4% for HbA1c), and whether the women had a child prior to the measurement (78.9% for reticulocyte count, 72.7% for platelet count, 96.7% for HbA1c). For women with multiple measurements, one was randomly selected, giving preference to measurements taken outside of pregnancy and excluding measurements taken during a pregnancy that resulted in stillbirth or multiple births. For measurements taken during pregnancy, gestational week at measurement was calculated

from date and gestational age at delivery. When gestational age at delivery was missing (52.9%), mean gestational age at delivery of the cohort (39.13 weeks) was used. For measurements outside of pregnancy, gestational week at measurement was coded as 0. When age at measurement was missing (12.1% for reticulocyte count, 19.1% for platelet count, 22.2% for HbA1c), data was imputed with multivariate imputation by chain equations including covariates. Ten complete imputed sets were analyzed and estimates were combined with `pool()` (R package 'mice' v3.13.0)[24]. One-sided *p*-value were calculated as $p_{new} = \frac{p_{old}}{2}$ in case of directional agreement with the effect observed in the UKBB.

SUPPLEMENTAL WEB RESOURCES

- Estonian Biobank, https://genomics.ut.ee/en/research/estonian-biobank
- Neale Lab UKBB summary statistics, http://www.nealelab.is/uk-biobank/
- NHGRI-EBI GWAS Catalog, https://www.ebi.ac.uk/gwas/
- UCSC genome annotation (hg19) database, https://hgdownload.cse.ucsc.edu/goldenPath/hg19/database/

SUPPLEMENTAL REFERENCES

1. Buniello, A., Macarthur, J.A.L., Cerezo, M., Harris, L.W., Hayhurst, J., Malangone, C., McMahon, A., Morales, J., Mountjoy, E., Sollis, E., et al. (2019). The NHGRI-EBI GWAS Catalog of published genome-wide association studies, targeted arrays and summary statistics 2019. Nucleic Acids Res. *47*, D1005–D1012.
2. Kolz, M., Johnson, T., Sanna, S., Teumer, A., Vitart, V., Perola, M., Mangino, M., Albrecht, E., Wallace, C., Farrall, M., et al. (2009). Meta-analysis of 28,141 individuals identifies common variants within five new loci that influence uric acid concentrations. PLoS Genet. *5*, e1000504.
3. Köttgen, A., Albrecht, E., Teumer, A., Vitart, V., Krumsiek, J., Hundertmark, C., Pistis, G., Ruggiero, D., O'Seaghdha, C.M., Haller, T., et al. (2013). Genome-wide association analyses identify 18 new loci associated with serum urate concentrations. Nat. Genet. *45*, 145–154.
4. Ketharnathan, S., Leask, M., Boocock, J., Phipps-Green, A.J., Antony, J., O'Sullivan, J.M., Merriman, T.R., and Horsfield, J.A. (2018). A non-coding genetic variant maximally associated with serum urate levels is functionally linked to HNF4A-dependent PDZK1 expression. Hum. Mol. Genet. *27*, 3964–3973.
5. Sinnott-Armstrong, N., Tanigawa, Y., Amar, D., Mars, N., Benner, C., Aguirre, M., Venkataraman, G.R., Wainberg, M., Ollila, H.M., Kiiskinen, T., et al. (2021). Genetics of 35 blood and urine biomarkers in the UK Biobank. Nat. Genet. *53*, 185–194.
6. Yuan, X., Waterworth, D., Perry, J.R.B., Lim, N., Song, K., Chambers, J.C., Zhang, W., Vollenweider, P., Stirnadel, H., Johnson, T., et al. (2008). Population-Based Genome-wide Association Studies Reveal Six Loci Influencing Plasma Levels of Liver Enzymes. Am. J. Hum. Genet. *83*, 520–528.
7. Chambers, J.C., Zhang, W., Sehmi, J.S., Li, X., Wass, M.N., Van der Harst, P., Holm, H., Sanna, S., Kavousi, M., Baumeister, S.E., et al. (2011). Genome-wide association study identifies loci influencing concentrations of liver enzymes in plasma. Nat. Genet. *43*, 1131–1138.
8. Kamatani, Y., Matsuda, K., Okada, Y., Kubo, M., Hosono, N., Daigo, Y., Nakamura, Y., and Kamatani, N. (2010). Genome-wide association study of hematological and biochemical traits in a Japanese population. Nat. Genet. *42*, 210–215.
9. Aguet, F., Barbeira, A., Bonazzola, R., Brown, A., Castel, S., Jo, B., Kasela, S., Kim-Hellmuth, S., Liang, Y., Oliva, M., et al. (2020). The GTEx Consortium atlas of genetic regulatory effects across human tissues. Science (80-.). *369*, 1318–1330.
10. Johnson, A.D., Kavousi, M., Smith, A. V., Chen, M.H., Dehghan, A., Aspelund, T., Lin, J.P., van Duijn, C.M., Harris, T.B., Cupples, L.A., et al. (2009). Genome-wide association meta-analysis for total serum bilirubin levels. Hum. Mol. Genet. *18*, 2700–2710.
11. Sanna, S., Busonero, F., Maschio, A., McArdle, P.F., Usala, G., Dei, M., Lai, S., Mulas, A., Piras, M.G., Perseu, L., et al.

(2009). Common variants in the SLCO1B3 locus are associated with bilirubin levels and unconjugated hyperbilirubinemia. Hum. Mol. Genet. *18*, 2711–2718.
12. Kang, T.W., Kim, H.J., Ju, H., Kim, J.H., Jeon, Y.J., Lee, H.C., Kim, K.K., Kim, J.W., Lee, S., Kim, J.Y., et al. (2010). Genome-wide association of serum bilirubin levels in Korean population. Hum. Mol. Genet. *19*, 3672–3678.
13. Bielinski, S.J., Chai, H.S., Pathak, J., Talwalkar, J.A., Limburg, P.J., Gullerud, R.E., Sicotte, H., Klee, E.W., Ross, J.L., Kocher, J.P.A., et al. (2011). Mayo genome consortia: A genotype-phenotype resource for genome-wide association studies with an application to the analysis of circulating bilirubin levels. Mayo Clin. Proc. *86*, 606–614.
14. Bycroft, C., Freeman, C., Petkova, D., Band, G., Elliott, L.T., Sharp, K., Motyer, A., Vukcevic, D., Delaneau, O., O'Connell, J., et al. (2018). The UK Biobank resource with deep phenotyping and genomic data. Nature *562*, 203–209.
15. Leitsalu, L., Haller, T., Esko, T., Tammesoo, M.L., Alavere, H., Snieder, H., Perola, M., Ng, P.C., Mägi, R., Milani, L., et al. (2015). Cohort profile: Estonian biobank of the Estonian genome center, university of Tartu. Int. J. Epidemiol. *44*, 1137–1147.
16. Wang, K., Li, M., Hadley, D., Liu, R., Glessner, J., Grant, S.F.A., Hakonarson, H., and Bucan, M. (2007). PennCNV: An integrated hidden Markov model designed for high-resolution copy number variation detection in whole-genome SNP genotyping data. Genome Res. *17*, 1665–1674.
17. Macé, A., Tuke, M.A., Beckmann, J.S., Lin, L., Jacquemont, S., Weedon, M.N., Reymond, A., and Kutalik, Z. (2016). New quality measure for SNP array based CNV detection. Bioinformatics *32*, 3298–3305.
18. Macé, A., Tuke, M.A., Deelen, P., Kristiansson, K., Mattsson, H., Nõukas, M., Sapkota, Y., Schick, U., Porcu, E., Rüeger, S., et al. (2017). CNV-association meta-analysis in 191,161 European adults reveals new loci associated with anthropometric traits. Nat. Commun. *8*, 1–11.
19. Gao, X., Starmer, J., and Martin, E.R. (2008). A multiple testing correction method for genetic association studies using correlated single nucleotide polymorphisms. Genet. Epidemiol. *32*, 361–369.
20. Handsaker, R.E., Van Doren, V., Berman, J.R., Genovese, G., Kashin, S., Boettger, L.M., and Mccarroll, S.A. (2015). Large multiallelic copy number variations in humans. Nat. Genet. *47*, 296–303.
21. Dufour, D.R., Lott, J.A., Nolte, F.S., Gretch, D.R., Koff, R.S., and Seeff, L.B. (2000). Diagnosis and monitoring of hepatic injury. II. Recommendations for use of laboratory tests in screening, diagnosis, and monitoring. Clin. Chem. *46*, 2050–2068.
22. Porcu, E., Rüeger, S., Lepik, K., Agbessi, M., Ahsan, H., Alves, I., Andiappan, A., Arindrarto, W., Awadalla, P., Battle, A., et al. (2019). Mendelian randomization integrating GWAS and eQTL data reveals genetic determinants of complex and clinical traits. Nat. Commun. *10*, 1–12.
23. Võsa, U., Claringbould, A., Westra, H.J., Bonder, M.J., Deelen, P., Zeng, B., Kirsten, H., Saha, A., Kreuzhuber, R., Yazar, S., et al. (2021). Large-scale cis- and trans-eQTL analyses identify thousands of genetic loci and polygenic scores that regulate blood gene expression. Nat. Genet. *53*, 1300–1310.
24. van Buuren, S., and Groothuis-Oudshoorn, K. (2011). mice: Multivariate imputation by chained equations in R. J. Stat. Softw. *45*, 1–67.
